# Synthesis and Application of a New Cyclic Phosphoric Acid in Enantioselective Three-Component Mannich Reactions

**DOI:** 10.3390/molecules30142928

**Published:** 2025-07-10

**Authors:** Giovanni Ghigo, Alessio Robiolio Bose, Stefano Dughera

**Affiliations:** Department of Chemistry, University of Torino, Via Pietro Giuria 7, 10125 Torino, Italy; alessio.robioliobose@edu.unito.it

**Keywords:** cyclic phosphoric acid, asymmetric catalysis, Mannich reaction

## Abstract

A novel point-chiral six-membered cyclic phosphoric acid was synthesized starting from an enantiopure precursor via a concise three-step route. Its catalytic performance was evaluated in enantioselective three-component Mannich reactions. Under optimized conditions, the catalyst provided good yields and satisfactory enantiomeric excesses (up to 89%). The basic mechanism of the catalysis was also studied by the DFT method.

## 1. Introduction

Organocatalysis, defined as the use of relatively small organic molecules to catalyze chemical reactions, is one of the fastest-growing areas in organic chemistry [1,2]. This technology, due to its many advantages, has established itself as the third pillar of asymmetric synthesis, alongside organometallic catalysis and biocatalysis [3,4,5,6].

The main advantages of organocatalysis can be summarized as follows: (i) environmental friendliness and low toxicity, (ii) high availability of organic reagents, (iii) low sensitivity to oxygen and moisture, and (iv) a large chiral pool.

**Figure 1:**
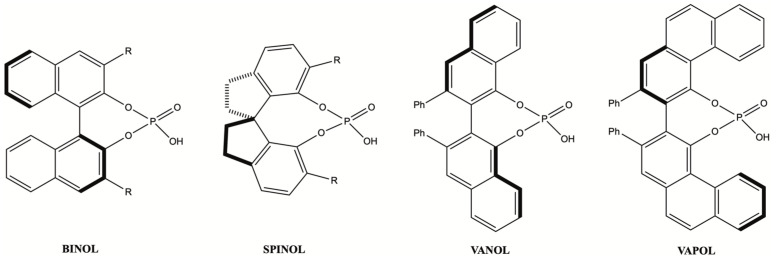
Various axially chiral CPAs.

According to List [7], organocatalysts can be classified into four main categories: Lewis acids, Lewis bases, Brønsted bases, and Brønsted acids. Among chiral Brønsted acids, chiral phosphoric acids (CPAs; Figure 1) have gained a prominent role and have been applied to a growing number of useful asymmetric protocols [8,9]. Most CPAs are based on axially chiral diols such as BINOL [10,11,12], SPINOL [13], VANOL [14] and VAPOL [15] and are characterized by a seven- or eight-membered cyclic phosphodiester core (in SPINOL derivatives, eight-membered).

**Figure 2:**
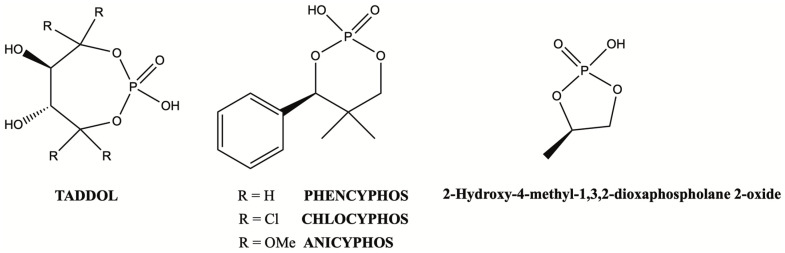
Various point-chiral cyclic CPAs.

On the other hand, there are only a few examples of point-chiral CPAs in the literature (Figure 2). Most of these are seven-membered rings derived from TADDOL [16]. Moreover, a small number of point-chiral six-membered cyclic phosphoric acids (e.g., phencyphos, chlocyphos, anicyphos) are known and used as resolving agents for chiral amines [17], but no applications in asymmetric synthesis have been reported.

Even rarer are examples of chiral five-membered cyclic phosphoric acids; essentially, only 2-hydroxy-4-methyl-1,3,2-dioxaphospholane 2-oxide is reported (Figure 2) [18].

**Figure 3:**
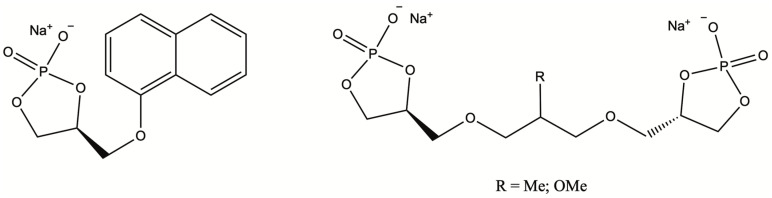
Five-membered cycloglycerophospates.

In light of this, in our previous research (Figure 3), we synthesized three chiral five-membered cycloglycerophospates [19,20,21]. The last two, which show C2 symmetry, proved to be excellent chiral catalysts. Unfortunately, we were unable to convert them into the corresponding acids, as they decomposed in acidic environments.

**Scheme 1:**
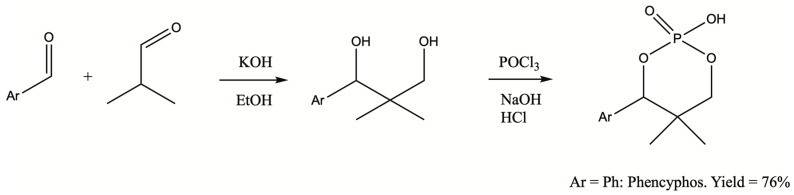
Synthesis of phencyphos.

Phencyphos and similar compounds were generally synthesized following Wynberg’s procedure [17], as shown in Scheme 1. The overall yields were quite good; in the best case (phencyphos), a yield of 76% was achieved. It should be noted that all the target compounds were obtained as racemic mixtures. To resolve these, various optically active bases were used (e.g., (−)-ephedrine, (+)-2-amino-1-phenyl-1,3-propanediol).

From this perspective, in the present work, we describe the synthesis of a new chiral six-membered cyclic phosphoric acid, starting from a chiral precursor, and its use as a chiral catalyst in multicomponent Mannich reactions.

## 2. Results and Discussion

**Scheme 2:**
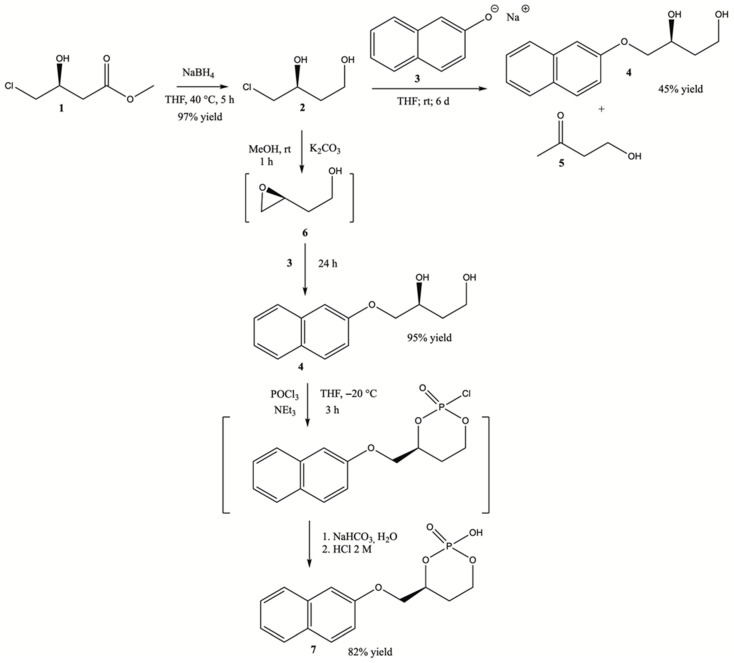
Synthesis of CPA **7**.

The point-chiral six-membered CPA **7** was synthesized via a three-step protocol (Scheme 2). We chose to start from chiral compound **1**, which is readily available and reasonably priced. In the first step, the ester group was reduced to an alcohol using NaBH_4_ in THF. Compound **2** was obtained in 97% yield.

In the second step, to eliminate a potentially reactive site (the chlorine atom), a nucleophilic substitution was carried out using sodium naphtholate (**3**). However, the results were unsatisfactory due to competition with the elimination reaction leading to ketone **5**.

To address this issue, epoxide **6** was first synthesized by treating **2** with potassium carbonate [22]. Once **6** was obtained—although it was not isolated—it was reacted with **3**, yielding the target adduct with an excellent yield. The introduction of a bulky group such as naphthol results in a structure with sufficient steric hindrance, which is useful for controlling enantioselectivity in subsequent reactions.

In the third and final step, adduct **4** was converted into chiral CPA **7** using POCl_3_ and triethylamine. Thereby, enantiomerically pure **7** was effectively obtained using a high-yield synthetic protocol (total yield calculated from **1** was 76%). The compound **7** was then tested as a chiral catalyst in a three-component Mannich reaction.

**Scheme 3:**
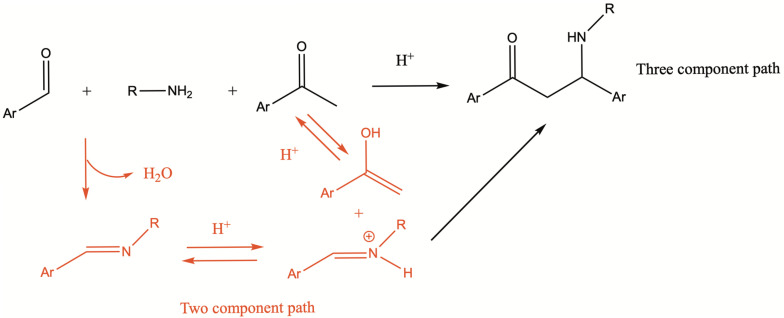
Two- or three-component Mannich reactions.

Mannich reactions are among the most important carbon–carbon bond-forming reactions in synthetic organic chemistry and are a standard method for synthesizing β-amino carbonyl compounds, which are key intermediates in the synthesis of many nitrogen-containing natural products and pharmaceuticals. These reactions can proceed via either a two- or a three-component protocol (Scheme 3; respectively, path red and path black) [23].

Stereoselective Mannich reactions catalyzed by chiral catalysts have received considerable attention, as they offer an efficient route for the enantioselective synthesis of β-amino carbonyl compounds [24,25]. Among these, chiral Brønsted acids have emerged as efficient catalysts, delivering high diastereo- and enantioselectivity. However, most chiral Brønsted acid-catalyzed Mannich reactions still involve imine catalysis in a two-component process [10,11,26].

Contrarily, the literature reports only a few examples of one-pot, three-component Mannich reactions catalyzed by chiral Brønsted acids [27,28,29,30,31].

With this in mind, we evaluated catalyst **7** in a three-component Mannich reaction. The model reaction involved benzaldehyde (**8a**), aniline (**9a**), and acetophenone (**10a**), using a catalytic amount of **7** under various conditions. As shown in Table 1, the best result was obtained under neat conditions at room temperature with 10 mol% of catalyst 1, which provided the target compound **11a** in good yield and satisfactory enantioselectivity (ee 90%; Table 1, entry 6). Interestingly, in the presence of commercial phencyphos as catalyst, both the yields and the enantiomeric excesses of **11a** were significantly lower (Table 1; entries 2–4).

To explore the scope and general applicability of this reaction, various substituted aromatic aldehydes **8**, aromatic or aliphatic amines **9** and ketones **10** were used. The results are summarized in Table 2.

In particular, reactions between benzaldehyde (**8a**), acetophenone (**10a**), and a number of substituted aromatic amines **9a**–**g** bearing either electron-withdrawing or electron-donating groups afforded satisfactory results in terms of both yield and enantioselectivity (Table 2; entries 1–7). Notably, the reaction outcomes were not significantly influenced by the electronic nature or the position of the substituents on the aromatic ring. Regarding aliphatic amines, the only appreciable results were obtained with benzylamine (**9h**; Table 2; entry 8).

Regarding the aromatic aldehydes **8**, the presence of the nitro group in the meta position (aldehyde **8c**) prevented the formation of the imine and consequently it was not possible to obtain adduct **11j** (Table 2; entry 10). With the nitro group in the para position (aldehyde **8f**), the intermediate imine was formed, even if slowly, and consequently it was possible to obtain adduct **11m** (Table 2; entry 13). Good results were obtained in the presence of electron-donating groups (aldehydes **8b**, **8d**), with a halogen (aldehyde **8e**; Table 2; entries 9,11,12) or with heteroaromatic aldehyde **8g** (Table 2; entry 14)**.** Electronic effects were also important for the ketones **10**. The presence of electron-withdrawing groups, such as nitrogroup (ketone **10c**), on the aromatic ring did not allow it to react with the imine intermediate (Table 2; entry 16). By contrast, good results were obtained in the precence of an electron donating group or a halogen (ketones **10b**, **10d**; Table 2; entries 15, 17). Moreover, by replacing **10a** with aliphatic **10e**, the main product of the reaction was 4-methylpent-3-en-2-one, which arose from the self-condensation of **10e** (Table 2; entry 18).

It must be stressed that satisfactory results in terms of enantioselectivity were always obtained, regardless of the electronic effects of the substituents.

**Figure 4:**
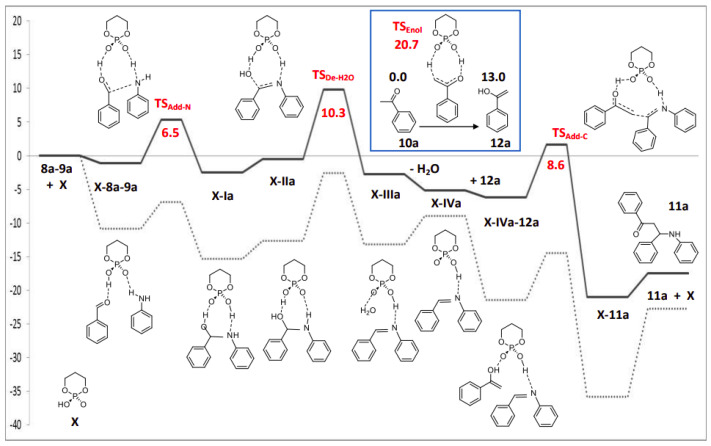
Energy profiles (in kcal mol^−1^) for the Mannich reaction among **8a**, **9a** and **10a** catalyzed by an achiral model **X** of the catalyst **7**. Dashed line, E+ZPE; thick line, free energy at 273.15 K (0 °C). Red values are free energy barriers with respect to the originating starting minima. The blue box reports the catalyzed enolization of **10a** to **12a** with the relative free energies.

**Figure 5:**
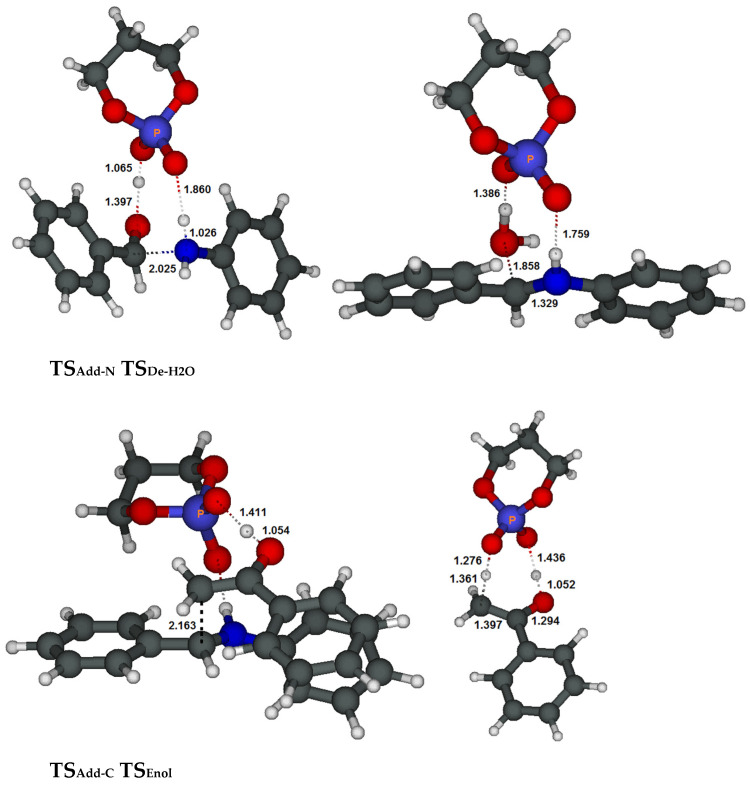
Transition structures for the Mannich reaction among **8a**, **9a** and **10a** catalyzed by an achiral model **X** of catalyst **7**.

The mechanism of activation of the reaction among **8a**, **9a** and **10a** by a non-chiral model of the catalyst **X** was studied by a computational DFT method (see the Supplementary Material SI § 1 for details on the method). The energy profiles and structures are illustrated in Figure 4. The pictures of the key transition structures, **TS_Add-N_**, **TS_De-H2O_**, **TS_Add-C_**, and **TS_Enol_**, are shown in Figure 5. The reaction followed the typical Mannich mechanism [23].

The first step was the nucleophilic addition of **9a** to **8a** through **TS_Add-N_**, where the catalyst allowed an indirect transfer of a proton from the amino group to the carbonyl, yielding the complex **X-Ia** between the adduct **Ia** and the catalyst. The second step, after reorientation of the catalyst in the complex **X-IIa**, consisted of dehydration through **TS_De-H2O_** yielding the complex **X-IIIa,** which then lost a water molecule, yielding the complex **X-IVa**. Again, the catalyst acted through indirect proton transfer from the amino group to the hydroxyl. Finally, the addition through **TS_Add-C_** of enol **12a** to the imine **IVa** yielded a complex between **X** and the product **11a**. Also, in this case the catalyst facilitated the proton transfer from the hydroxyl group of the enol to the imine/amine. The whole reaction was quite fast: the estimated rate constants at 273 K (0 °C) for the three steps (with [X] = 0.03 M) were *k*_Add-N_ = 9.0 • 10^7^ M^−1^ s^−1^; *k*_De-H2O_ = 7.8 • 10^2^ s^−1^; *k*_Add-C_ = 4.9 • 10^8^ M^−1^ s^−1^.

Apparently, the rate-determining state is the dehydration, which determines the formation of the imine, as we will see when analyzing the reaction for other reactants (below). However, the real limiting phase, external to the mechanism described above, is the enolization of **10a** to **12a** through **TS_Enol_** (blue box in Figure 4). Despite the role of indirect proton transfer by the catalyst, this process is very slow (*k*_Enol_ = 1.0 • 10^−4^ M^−1^ s^−1^) and thermodynamically disfavored (K_Enol_ = 4.3 • 10^−11^). Therefore, even if the imine is quickly formed, one will find a very small concentration of the enol **12a**. This explains why the reaction requires a long time (24 h or more).

In order to explain why in some selected cases (Table 2, entries 10, 13, 16) less or no product was obtained, we extended the computational study to some critical points of the reaction.

The formation of the imines **IV** is the first phase of the reaction and the rate-determining step is the dehydration (**TS_De-H2O_**). If we compare the free energies for this step (Table 3, bold numbers), for the reaction of **9a** with **8a** (Table 2, entry 1), with **8f** (Table 2, entry 13) or **8c** (Table 2, entry 10), we can observe that, for the latter, the free energy of **TS_De-H2O_** was 2.5 kcal mol^−1^ higher than that for **8a**.

This means that the formation of the imine is 95 times slower. This can explain why no product or imine was recovered (Table 2; entry 10 and note 4,). With **8f**, the free energy of **TS_De-H2O_** was 1.3 kcal mol^−1^ higher than that for **8a**. This means that the imine is formed more slowly (9 times slower), yielding **11m** in smaller yields (Table 2; entry 13).

In the reaction with **10c** (Table 2, entry 16), despite a slightly less unfavorable free energy (**12c** is 12.1 kcal mol^−1^ above **10c** compared to 13.0 kcal mol^−1^ for **12a** above **10a**), the free energy barrier for enolization was 1.3 kcal mol^−1^ higher (22.1 kcal mol^−1^). This led to a rate for the formation of the enol **12c** 12 times slower than that for **12a**. Therefore, once formed from **8a** and **9a**, the imine (Table 2; entry 16, note 5) could not find enough enol to complete the reaction.

In entry 18, the ketone is acetone (**10e**). In this case, only the product of the aldol condensation was identified (Table 2; entry 18, note 6). The comparison of the activation free energies (Table 4) for the additions of the enols **12a** or **12e** to the imine **IVa** (**TS_Add_**) or to the ketones **10a** and **10e** (**TS_AldC_**) shows that the former was preferred when the ketone was benzophenone **10a** (its enol **12a**), while when the ketone was acetone (**10e** and **12e**) the addition leading to enol condensation was preferred. This could be due to the higher reactivity of acetone, whose carbonylic carbon atom presents a partial charge of 0.61 e^−^ and a localized π*_CO_, while in benzophenone the partial charge on the carbonylic carbon is slightly smaller (0.59) and the π*_CO_ is partially delocalized on the aromatic ring.

Based on the mechanism described in Figure 4, it can be reasonably assumed that in **TS_Add-C_**, the nucleophilic attack of enol **12a** occurred predominantly from only one direction, leading to the formation of **11a** with good enantiomeric excesses. Therefore, further studies were carried out with a chiral model of catalyst **7**. However, in the optimization of **TS_Add-C_** leading to products R and S, we identified several conformations, all close in energy and free energy, that did not give clear indications (see Supplementary Material, Table on page S-8). While there was a difference of 1.3 kcal mol^−1^ in terms of energy in favour of the lowest transition structure yielding the R product, **TS(R)(a),** with respect to that yielding the S product, **TS(S)(a)**, the free energy values reduced the difference to less than 0.1 kcal mol^−1^, and more so between different TS, **TS(R)(e)** and **TS(R)(d)**.

This is possibly due to the fact that to reproduce the enantiomeric excesses requires an accuracy in the calculation of the transition structures (less than 1 kcal mol^−1^) that goes beyond that of available computational methods (ca. 2 kcal mol^−1^) [32].

## 3. Materials and Methods

### 3.1. General

All reagents and solvents were purchased from commercial sources (Merck, Milano, Italy; Thermo Fisher Scientific, Monza, Italy; Carlo Erba Reagents, Cornaredo (MI), Italy) at the highest available purity grade and used without further purification. All reactions were carried out in open air glassware and monitored by GC-FID and GC-MS. TLC were performed on Merck silica gel 60 (70-230 mesh ASTM) and GF 254. Mass spectra were recorded on an HP 5989B mass selective detector (Hewlett-Packard, Cernusco sul Naviglio (MI), Italy) connected to an HP 5890 GC (Hewlett-Packard, Cernusco sul Naviglio (MI), Italy) with a methyl silicone capillary column. GC FID analyses were performed on a Perkin Elmer AutoSystem XL GC (Perkin-Elmer, Milano, Italy) with a methylsilicone capillary column. ^1^H NMR and ^13^C NMR spectra were recorded on a Brucker spectrometer at 400 and 100 MHz (Brucker, Milano, Italy). Chiral analyses were performed on an Essential LC-16 series HPLC (Shimadzu, Milano, Italy) using a Daicel CHIRALPAK-IG (250 x 4.6 mm, 5 mm; Daicel Corporation, Japan, Tokyo) eluting with i-PrOH and n-hexane. For determination of the optical rotation, a Jasco P-2000 polarimeter (Jasco, Cremella (LC), Italy) was used. IR spectra were recorded on an IR Perkin-Elmer UATR-two spectrometer (Perkin Elmer, Milano, Italy). The structures and purity of all products obtained in this research were confirmed by spectroscopic data (NMR, GC-MS, IR) datathat are reported in the Supplementary Material. Satisfactory microanalyses were obtained for all new compounds.

### 3.2. (+)-2-Hydroxy-4-((naphthalen-2-yloxy)methyl)-1,3,2-dioxaphosphinane 2-oxide (***7***)

#### 3.2.1. Synthesis of (S) (−)-4-Chlorobutane-1,3-diol (**2**)

As reported in the literature [33], THF (15 mL) and, under stirring, NaBH_4_ (1.14 g, 30 mmol) were added in a 50 mL flask. The resulting suspension was stirred at 40 °C for 1 h. Then, to this suspension, (S) ethyl 4-chloro-3-hydroxybutirrate (**1**; 3.34 g, 20 mmol), dissolved in THF (10 mL) was added dropwise. The mixture was stirred at 40 °C for 5 h, until the TLC (eluent PE/ethyl acetate 3:2), GC and GC-MS analyses showed the complete disappearance of **1**. After acidifying (pH 5) with HCl 2M (1 mL), a white solid precipitated, which was removed by filtration with a Bückner funnel. The residual THF resulting from filtration was evaporated under reduced pressure. The resulting colorless oil was the virtually pure title compound (**2**; 2.39 g, 96% yield).

[α]D_21_ = −22.9 (c = 1.00 in MeOH; Lit. [33] 22.3 for R enantiomer); ^1^H NMR (400 MHz, CDCl_3_): δ = 4.12–4.06 (m, 1H), 3.94–3.84 (m, 2H), 3.64 (dd, *J_1_* = 11.2 Hz, *J_2_* = 4.0 Hz, 1H), 3.55 (dd, *J_1_* = 11.2 Hz, *J_2_* = 4.0 Hz, 1H), 2.37 (br s, 2H), 1.85–1.80 (m, 2H); ^13^C NMR (100 MHz, CDCl_3_): δ = 68.7, 58.3, 47.7, 33.7; IR (neat) ν = 3345 (OH), 3321 (OH) cm^−1^. MS: *m*/*z* 124 (M^+^·).

#### 3.2.2. Synthesis of (−)-4-(Naphthalen-2-yloxy)butane-1,3-diol (**4**)

As reported in the literature [22], anhydrous K_2_CO_3_ (2.76 g, 20 mmol) and MeOH (10 mL) were added in a 50 mL flask. To the resulting turbid solution, **2** (1.24 g, 10 mmol) dissolved in MeOH (5 mL) was added at rt under stirring. After 2 h, TLC (eluent PE/ethyl acetate 3:2) and GC-MS analyses showed the disappearance of **2** and the presence of epoxide **6** (MS: *m*/*z* 88 (M^+^)), which was not isolated, but was reacted in situ. Then, sodium naphthalen-2-olate (1.99, 12 mmol) was added in a single portion. The mixture was stirred at rt for a further 3 h, until the TLC (eluent PE/ethyl acetate 3:2), GC and GC-MS analyses showed the complete disappearance of **6**. MeOH was evaporated under reduced pressure and the crude residue was purified in a short chromatography column (eluent PE/ethyl acetate 1:4). The resulting white solid was the pure title compound (**4**; 2.20 g, 95% yield). 

[α]D_21_ = −12.9 (c = 1.00 in MeOH); ^1^H NMR (400 MHz, CD_3_OD): δ =7.78–7.75 (m, 3H), 7.44 (t, *J =* 7.6 Hz, 1H), 7.32 (t, *J =* 7.6 Hz, 1H), 7.21 (d, *J =* 2.4 Hz, 1H), 7.18 (d, *J =* 2.4 Hz, 1H), 4.22–4.17 (m, 1H), 4.13–4.03 (m, 2H), 3.82–3.79 (m, 2H), 1.97–1.89 (m, 1H), 1.87–1.79 (m, 1H); ^13^C NMR (100 MHz, CD_3_OD): δ = 156.9, 134.7, 129.1, 128.9, 127.2, 126.5, 125.9, 123.3, 118.5, 106.4, 72.0, 67.1, 58.5, 35.9. IR (neat) ν = 3461 (OH), 3408 (OH) cm^−1^. Elemental analysis calcd (%) for C_14_H_16_O_3_: C 72.39; H 6.94; found: C 71.99; H 7.02.

#### 3.2.3. Synthesis of (+)-2-Hydroxy-4-((naphthalen-2-yloxy)methyl)-1,3,2-dioxaphosphinane-2-oxide (**7**)

As reported in the literature [20], THF (5 mL) and POCl_3_ (0.77 g, 5 mmol) were added in a 50 mL flask. The mixture was cooled to −20 °C and NEt_3_ was added (0.51 g, 5 mmol). At this point, a solution of **4** (0.23 g, 1 mmol) in THF (5 mL) was added dropwise. The reaction mixture was stirred at −20 °C for 1 h and at rt for additional 2 h. Then, it was carefully quenched in a saturated solution of NaHCO_3_ (about 20 mL) and was extracted with ethyl acetate (15 mL). At the end of extraction, the organic phase was eliminated and H_2_O was evaporated under reduced pressure. In order to separate the target product from undesired byproducts, a trituration with methanol (50 mL) was performed. The resulting white waxy solid was dissolved in H_2_O (2 mL). The solution was acidified with HCl 2M (2 mL). A white solid precipitate was isolated by filtration with a Büchner funnel and was the pure title compound (**7**; 0.24 g, 82% yield).

[α]D_21_ = + 9.4 (c = 1.00 in MeOH); ^1^H NMR (400 MHz, CD_3_OD): 7.66–7.44 (m, 3H), 7.32 (t, *J =* 7.6 Hz, 1H), 7.22 (t, *J =* 7.6 Hz, 1H), 7.06 (d, *J =* 2.4 Hz, 1H), 7.04 (d, *J =* 2.4 Hz, 1H), 7.30 (m, 1H), 7.24–7.20 (m, 1H), 7.15–7.14 (m,1H), 7.07–7.04 (m, 1H), 4.82–4.78 (m, 1H), 4.41–4.27 (m, 2H), 4.13–4.12 (m, 2H), 2.18–2.08 (m, 1H), 1.89–1.86 (m, 1H); ^13^C NMR (100 MHz, CD_3_OD): 156.4, 134.6, 129.3, 129.1, 127.2, 126.5, 126.0, 123.5, 118.2, 106.6, 77.7 (d, *J* = 5.7 Hz), 69.7 (d, *J* = 9.8 Hz), 67.0 (d, *J* = 6.1 Hz), 27.8 (d, *J* = 5.6 Hz). ^31^P NMR (162 MHz, CD_3_OD): −5.1. IR (neat) ν = 3042 (OH)cm^−1^; elemental analysis calcd (%) for C_14_H_15_O_5_P: C 57.15; H 5.14; found: C 56.99; H 5.21.

### 3.3. (+)-***7*** as a Catalyst in Mannich Reaction: General Procedure

(+)-**7** (10 mol%, 30 mg, 0.1 mmol) was added to stirring mixtures of aldehydes **8** (1 mmol), amines **9** (1 mmol) and ketones **10** (1 mmol). The mixtures were stirred at r.t. for the times listed in Table 2 until the GC and GC-MS analyses showed the complete disappearance of the starting compounds and the complete formation of β-aminoketones **11**. Cold H_2_O (2 mL) was added to the reaction mixture, under vigorous stirring. The resulting solids were filtered with a Hirsch funnel and washed with additional cold H_2_O (1 mL) and petroleum ether (1 mL). Virtually pure (TLC, GC, GC-MS, ^1^H NMR, ^13^C NMR) β-aminoketones **11** were obtained.

## 4. Conclusions

We synthesized a new point-chiral six-membered cyclic phosphoric acid in three steps from a commercially available chiral precursor (total yield 76%). This catalyst demonstrated good performance in asymmetric three-component Mannich reactions, delivering products with good yields (15 positive examples; average yield 80%) and satisfacyory enantioselectivity (15 positive examples; average ee 92.1%). Its operational simplicity, good functional group tolerance, and potential for structural modification make it a valuable addition to the toolbox of chiral Brønsted acid catalysts. Finally, it must be stressed that this new catalyst has a performance comparable to those of axially chirally BINOL-derived phosphoric acids widely used as asymmetric catalysts in the Mannich reaction [10,11,27].

## Figures and Tables

**Table 1:** Trial reactions.

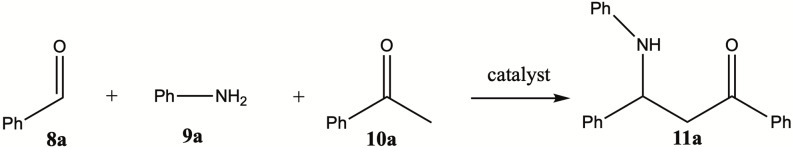							
Entry	Catalyst	Amount (%)	Solvent	T (°C)	Time (h)	Yield (%) of 11a ^1,2^	Ee (%) ^3^
1	**-**	-	neat	20	4	- ^4^	-
2	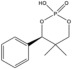	5	toluene	20	24	27	38.2
3	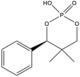	5	neat	20	24	33	49.1
4	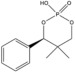	10	neat	20	24	40	49.3
5	**7**	5	neat	20	24	40	89.4
6	**7**	10	neat	20	24	85	90.1
7	**7**	10	neat	0	48	- ^5^	-
8	**7**	10	DCM	20	48	- ^5^	-
9	**7**	10	toluene	20	24 h	40	72.5

^1^ Reactants **8a**, **9a**, **10a** were in equimolecular amount (1 mmol); ^2^ Yield refers to pure and isolated **11a** obtained after filtering via Büchner funnel and washing the crude residues with a small amount of H_2_O and petroleum ether; ^3^ The enantiomeric excesses (ee) were determined by chiral analyses on HPLC connected to a chiral column. **11a** is a (+) enantiomer; ^4^ GC-MS analysis of the crude residue showed the presence of *N*,1-diphenylmethanimine, MS: *m*/*z* 181 (M)^+^ and unreacted **10a**. *N*,1-diphenylmethanimine was isolated in approximate quantitative yield. Even extending the reaction time to 24 h, only *N*,1-diphenylmethanimine was obtained; ^5^ The reaction was not complete.

**Table 2:** Three-component Mannich reaction between **8**, **9**, and **10** catalyzed by **7**.

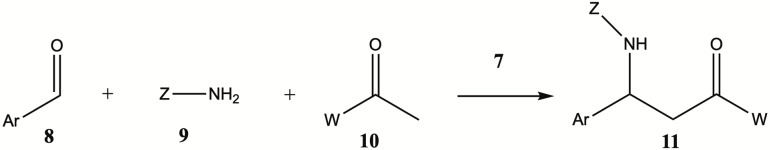							
Entry	Ar in 8	Z in 9	W in 10	Time (h)	Products 11	Yield ^1,2^ (%)	Ee ^3^ (%)
1	C_6_H_5_ **8a**	C_6_H*_5_* **9a**	C_6_H*_5_* **10a**	24	**11a**	85	90.3
2	C_6_H_5_ **8a**	2-NO_2_C_6_H_4_ **9b**	C_6_H*_5_* **10a**	24	**11b**	87	91.5
3	C_6_H_5_ **8a**	3-FC_6_H_4_ **9c**	C_6_H*_5_* **10a**	24	**11c**	91	91.7
4	C_6_H_5_ **8a**	4-MeC_6_H_4_ **9d**	C_6_H*_5_* **10a**	36	**11d**	82	92.9
5	C_6_H_5_ **8a**	4-NO_2_C_6_H_4_ **9e**	C_6_H*_5_* **10a**	24	**11e**	91	95.0
6	C_6_H_5_ **8a**	4-BrC_6_H_4_ **9f**	C_6_H*_5_* **10a**	24	**11f**	92	93.1
7	C_6_H_5_ **8a**	2,6-(CH_3_)_2_C_6_H_3_ **9g**	C_6_H*_5_* **10a**	24	**11g**	82	89.7
8	C_6_H_5_ **8a**	C_6_H_5_CH_2_ **9h**	C_6_H*_5_* **10a**	36	**11h**	87	92.6
9	3-MeOC_6_H_4_ **8b**	C_6_H*_5_* **9a**	C_6_H*_5_* **10a**	36	**11i**	92	92.9
10	3-NO_2_C_6_H_4_ **8c**	C_6_H*_5_* **9a**	C_6_H*_5_* **10a**	24	**11j**	- ^4^	-
11	4-MeC_6_H_4_ **8d**	C_6_H*_5_* **9a**	C_6_H*_5_* **10a**	48	**11k**	81	91.7
12	4-ClC_6_H_4_ **8e**	C_6_H*_5_* **9a**	C_6_H*_5_* **10a**	48	**11l**	87	92.6
13	4-NO_2_C_6_H_4_ **8f**	C_6_H*_5_* **9a**	C_6_H*_5_* **10a**	48	**11m**	67	95.1
14	2-thienyl **8g**	C_6_H*_5_* **9a**	C_6_H*_5_* **10a**	24	**11n**	90	89.6
15	C_6_H_5_ **8a**	C_6_H*_5_* **9a**	3-MeOC_6_H_4_ **10b**	24	**11o**	93	90.1
16	C_6_H_5_ **8a**	C_6_H*_5_* **9a**	4-NO_2_C_6_H_4_ **10c**	24	**11p**	- ^5^	-
17	C_6_H_5_ **8a**	C_6_H*_5_* **9a**	4-ClC_6_H_4_ **10d**	48	**11q**	92	92.8
18	C_6_H_5_ **8a**	C_6_H*_5_* **9a**	Me **10e**	24	**11r**	- ^6^	-

^1^ Reactants **8**, **9**, **10** were in equimolecular amounts (1 mmol) in the presence of 10 mol% of catalyst **7**; ^2^ Yields refers to pure and isolated **11** obtained after filtering via Büchner funnel and washing the crude residues with a small amount of H_2_O and petroleum ether; ^3^ The enantiomeric excesses (ee) were determined by chiral analyses on HPLC connected to a chiral column. All compounds **11** are (+) enantiomers; ^4^ Only the starting products **8c**, **9a**, **10a** were recovered; ^5^ Only *N*,1-diphenylmethanimine was formed; MS: *m*/*z* 181 (M)^+^. ^6^ The main product was 4-methylpent-3-en-2-one; MS: *m*/*z* 98 (M)^+^.

**Table 3:** The free energies (in kcal mol^−1^, 273 K) for the formation of the complex **X-IV** between the catalyst **X** and the imines **IV** from **9a** and **8a**, **8f** or **8c**.

	Benzaldehyde (8a)	4-Nitrobenzaldehyde (8f)	3-Nitrobenzaldehyde (8c)
**TS_Add-N_**	5.8	4.9	6.4
**X-I**	−2.1	−3.1	−1.0
**TS_De-H2O_**	**10.2**	**11.4**	**12.7**
**X-IV**	−4.7	−8.7	−3.1

**Table 4:** The free energy barriers (in kcal mol^−1^, 273 K) for the addition of the enols **12a** (from benzophenone **10a**) and **12e** (from acetone **10e**) to the imine **IVa** and to the ketones **10a** and **10e**.

	Acetophenone (10a)	Acetone (10e)
**TS_Add_**	6.8	7.5
**TS_AldC_**	9.2	6.9

## Data Availability

The data reported in this article can be obtained from the authors upon reasonable request. Samples of the catalyst (+)-**7** and adducts **11** are available from the authors.

## Supplementary Materials

The following supporting information can be downloaded at: https://www.mdpi.com/article/10.3390/molecules30142928/s1. Computational method: S-2; tables with absolute and relative energies: S-4; pictures and Cartesian coordinates of structures: S-11; NMR spectra of precursors **2**, **4** and catalyst **7**: S-49; physical and spectroscopic data of compounds **11**: S-55; NMR spectra of compounds **11**: S-59; chiral analyses of compounds **11**: S-74. Includes refs. [34,35,36,37,38,39,40,41,42,43,44,45,46,47,48,49,50].
