# Supplementary material for: Synthesis and Application of a New Cyclic Phosphoric Acid in Enantioselective Three-Component Mannich Reactions
Source: Molecules. 2025 Jul 10;30(14):2928. doi: 10.3390/molecules30142928 (PMC12300773; doi:10.3390/molecules30142928)
Supplement: Supplementary file 1 [file molecules-30-02928-s001.zip › molecules-3723748-supplementary.pdf]

Supplementary Material

Synthesis and Application of a New Cyclic Phosphoric Acid in Enantioselective three component Mannich Reactions.

Giovanni Ghigo, Alessio Robiolio Bose, Stefano Dughera.

Department of Chemistry, University of Torino, via P. Giuria 7, 10125, Torino, Italy.

INDEX

1. **Computational method.** S-2
2. **Tables with absolute and relative energies**.
   - **2.1.** The thermochemistry for the *Mannich* reaction among **8a**, **9a** and **10a** to **11a**. S-4
   - **2.2.** The enolization of **10a** to **12a**. S-4
   - **2.3.** The *Mannich* reaction among **8a**, **9a** and **12a** to **11a**. S-5
   - **2.4.** The aldol condensation between **10a** and **12a**. S-6
   - **2.5.** The formation of the imine from **9a** and **8b** or **8e**. S-7
   - **2.6.** The enolization of **10b** to **12b**. S-8
   - **2.7.** The addition of the enol **12e** to **IVa** (*Mannich*) or to **10e** (aldol condensation). S-8
   - **2.8.** The addition of the enol **12a** to **IVa** catalized by CPA **7**. S-9
3. **Pictures and Cartesian coordinates of structures.**
   - **3.1.** The specie involved in the *Mannich* reaction among **8a**, **9a** and **10a** (and **12a**) to **11a**. S-11
   - **3.2.** The specie involved in the *Mannich* reaction among **9a** and **8b** or **8e**. S-29
   - **3.3.** The specie involved in the aldol condensation between **10a** and **12a**. S-40
   - **3.4.** The specie involved in the enolization of **10b** to **12b**. S-42
   - **3.5.** The specie involved in the addition of the enol **12e** to **IVa** or to **10e**. S-45
4. **NMR spectra of precursors 2, 4 and catalyst 7.** S-49
5. **Physical and spectroscopic data of compounds 11.** S-55
6. **NMR spectra of compounds 11.** S-59
7. **Chiral analyses of compounds 11.** S-74

1. Computational method.

The structures of the reactants, intermediates and transition states have been optimized by using the density functional method (DFT) [34] with the functional M06-2X [35,36] and the basis sets def2-SVP [37] improved by a set of diffuse functions on the non-Hydrogen atoms. The nature of the critical points was characterized by using vibrational analysis [38] which also furnished the Zero Point Energies (ZPE) and entropies for the calculations of the Free Energies. These have been converted from the gas phase to the 1 M standard state at 1 atm and 298.15 K. [39] Then, the structures have been reoptimized with the basis set def2-TZVP [37] improved by a set of diffuse functions on the non-Hydrogen atoms. and the energies have been combined with the thermal corrections obtained with the smaller basis set. The solvent effects (acetophenone and acetone) were introduced in all calculations using the universal solvation model (SMD) by Truhalr *et al.* [40] The rate constants of the reactions are calculated using canonical transition state theory. [41]

$$k = \sigma \cdot (k_B \cdot T / h_P) \cdot (RT)^{n-1} \cdot \exp(-\Delta G^{\ddagger} / RT)$$

in which σ is the symmetry number, k_B the Boltzmann constant, h_P the Planck constant, n is 1 for for monomolecular reactions, 2 for bimolecular and 3 for threemolecular reactions.

The calculations were performed by the quantum package Gaussian 16-A.03 [42] The figures were obtained using the graphical program Molden. [43]

References

[34] Parr, R. G. Density Functional Theory of Atoms and Molecules, in: Horizons Quantum Chem., Springer Netherlands, **1980**: pp. 5–15. https://doi.org/10.1007/978-94-009-9027-2_2.

[35] Zhao, Y.; Truhlar, D. G. The M06 suite of density functionals for main group thermochemistry, thermochemical kinetics, noncovalent interactions, excited states, and transition elements: Two new functionals and systematic testing of four M06-class functionals and 12 other function." *Theor. Chem. Acc.* **2008**, *120*, 215–241. https://doi.org/10.1007/s00214-007-0310-x.

[36] Zhao, Y.; Truhlar, D. G. "Density functionals with broad applicability in chemistry." *Acc. Chem. Res.* **2008**, *41*, 157–167. https://doi.org/10.1021/ar700111a.

[37] Schaefer, A.; Huber, C.; Ahlrichs, R. Fully optimized contracted Gaussian-basis sets of triple zeta valence quality for atoms Li to Kr. *J. Chem. Phys.* **1994**, *100*, 5829–5835. https://doi.org/10.1063/1.467146.

[38] Foresman, J.; Frisch, A. Exploring chemistry with electronic structure methods, 1996, Gaussian Inc, Pittsburgh, PA, **1996**. http://gaussian.com/expchem3/(accessed June 4, 2021).

[39] Ribeiro, R. F.; Marenich, A. V.; Cramer, C. J.; Truhlar, D. G. Use of solution-phase vibrational frequencies in continuum models for the free energy of solvation. *J. Phys. Chem. B.* **2011**, *115*, 14556–14562. https://doi.org/10.1021/jp205508z.

[40] Marenich, A. V.; Cramer, C. J.; Truhlar, D. G. Universal solvation model based on solute electron density and a continuum model of the solvent defined by the bulk dielectric constant and atomic surface tensions. *J. Phys. Chem. B* **2009**, *113*, 6378–6396. https://doi.org/10.1021/jp810292n.

[41] Truhlar, D. G.; Garrett, B. C.; Klippenstein, S. J. Current status of Transition-State Theory *J. Phys. Chem.* **1996***, 100*, 12771–12800. https://doi.org/10.1021/jp953748q

[42] Frisch, M. J.; Trucks, G. W.; Schlegel, H. B.; Scuseria, G. E.; Robb, M. A.; Cheeseman, J. R.; Scalmani, G.; Barone, V.; Petersson, G. A.; Nakatsuji, H.; Li, X.; Caricato, M.; Marenich, A. V.; Bloino, J.; Janesko, B. G.; Gomperts, R.; Mennucci, B.; Hratch, Gaussian 16, Revision A.03, (**2016**).

[43] Schaftenaar, G.; Noordik, J. H. Molden: A pre- and post-processing program for molecular and electronic structures. *J. Comput. Aided. Mol. Des.* **2000**, *14*, 123–134. https://doi.org/10.1023/A:1008193805436.

2. Tables with absolute and relative energies.

2.1. The thermochemistry for the *Mannich* reaction among **8a**, **9a** and **10a** to **11a.**

These data do not take in acount that the reaction if perfored without solvet (neat).

**Table S-2.1**		E DZ /au[a]	ΔE	ZPE/au	Δn	δG^{273K} /au	E TZ /au[b]	ΔE	ΔE^{0K}	ΔG^{273K}
Benzaldeheyde	**8a**	-345.189033		0.110953		0.083524	-345.568554			
Aniline	**9a**	-287.284573		0.118061		0.091929	-287.599538			
Acetophenone	**10a**	-384.463629		0.138853		0.109585	-384.886934			
Enol-Acetophenone	**12a**	-384.443286		0.139261		0.110071	-384.866766			
**8a + 9a + 10a**		-1016.937235	**0.00**	0.367867	**0**	0.285038	-1018.055026	**0.00**	**0.00**	**0.00**
H_2O		-76.348129		0.021286		0.004820	-76.436030			
anti-C,anti-O 4a	**11a**	-940.619609		0.348984		0.304587	-941.640831			
**11a + H_2O**		-1016.967738	**-19.14**	0.370270	**-1**	0.309407	-1018.076862	**-13.70**	**-12.19**	**-0.10**

Absolute energy values in a.u., relative energy values in kcal mol^{-1}. [a] def2-SVP data; [b] def2-TZVP data.

2.2. The enolization of **10a** to **12a.**

**Table S-2.2**		E DZ /au[a]	ΔE	ZPE/au	Δn	δG^{273K} /au	E TZ /au[b]	ΔE	ΔE^{0K}	ΔG^{273K}
Model Cat	**X**	-760.315004		0.115649		0.086978	-760.940271			
**X + 10a**		-1144.778633	**0.00**	0.254502	**0**	0.196563	-1145.827206	**0.00**	**0.00**	**0.00**
**TS_{Enol} [X + 10a]**	**TS_{Enol}**	-1144.759911	**11.75**	0.251170	**-1**	0.211164	-1145.806109	**13.24**	**11.15**	**20.71**
**X + 12a**		-1144.758289	**12.77**	0.254910	**0**	0.197049	-1145.807037	**12.66**	**12.91**	**12.96**

Absolute energy values in a.u., relative energy values in kcal mol^{-1}. [a] def2-SVP data; [b] def2-TZVP data.

2.3. The *Mannich* reaction among **8a**, **9a** and **12a** to **11a**.

The initial reactant (for the energy reference) is a complex between **8a** and **9a** as, being in neat conditions, they are assumed to be as already in contact. In the second part of the table, the energy (bold figures in kcal mol^{-1}) are recalculated adding the energy of H_2O and starting from the energies of the comples **X+IVa**. These values are taht reported in the profile in Figure 3.

**Table S-2.3**		E DZ /au[a]	ΔE	ZPE/au	Δn	δG^{273K} /au	E TZ /au[b]	ΔE	ΔE^{0K}	ΔG^{273K}
Model Cat	**X**	-760.315004		0.115649		0.086978	-760.940271			
Cpl [8a + 9a]		-632.485041		0.229875		0.192840	-633.176469			
**X + Cpl [8a + 9a]**	**8a+9a**	-1392.800044	**0.00**	0.345524	**0**	0.279818	-1394.116741	**0.00**	**0.00**	**0.00**
**Cpl [X + 8a + 9a]**	**X+8a+9a**	-1392.816708	**-10.46**	0.347687	**-1**	0.297040	-1394.132380	**-9.81**	**-8.46**	**-0.69**
**TS_{Add} [X + 8a + 9a]**	**TS_{Add-N}**	-1392.813686	**-8.56**	0.348248	**-1**	0.301622	-1394.126632	**-6.21**	**-4.50**	**5.79**
**Cpl [X + Int. Ia]**	**X+Ia**	-1392.831633	**-19.82**	0.350684	**-1**	0.304966	-1394.142583	**-16.22**	**-12.98**	**-2.12**
**Cpl [X + Int. IIa]**	**X+IIa**	-1392.828230	**-17.69**	0.351281	**-1**	0.304521	-1394.138959	**-13.94**	**-10.33**	**-0.13**
**TS_{De-H2O} [X + Int. IIa]**	**TS_{De-H2O}**	-1392.808481	**-5.29**	0.349070	**-1**	0.302691	-1394.120661	**-2.46**	**-0.24**	**10.20**
**Cpl [X + Int. IIIa]**	**X+IIIa**	-1392.820701	**-12.96**	0.346279	**-1**	0.296739	-1394.134698	**-11.27**	**-10.79**	**-2.34**
H_2O		-76.348129		0.021286		0.004820	-76.436030			
Cpl [X + Int. IV]	**X+IVa**	-1316.462409		0.322017		0.275720	-1317.688946			
**Cpl [X + Int. IVa] + H_2O**		-1392.810538	**-6.59**	0.343303	**0**	0.280540	-1394.124977	**-5.17**	**-6.56**	**-4.72**
N-PhenylBenzaldimine	**IVa**	-556.125832		0.204743		0.170571	-556.730421			
**X + Int. IVa + H_2O**		-1392.788965	**6.95**	0.341678	**1**	0.262369	-1394.106723	**6.29**	**3.87**	**-2.98**
Enol-Acetophenone	**12a**	-384.443286		0.139261		0.110071	-384.866766			

**[X + Int. IVa] + 12a**		-1700.905695	0.00	0.461278	**0**	0.385791	-1702.555712	0.00	0.00	0.00
Cpl [X + Int. IVa + 5a]		-1700.933234	-17.28	0.462589	**-1**	0.407949	-1702.578107	-14.05	-13.23	-1.84
**[X + Int. IVa + 5a] + H_2O**			**-23.87**					**-19.22**	**-19.79**	**-6.55**
TS_{Add-C} [X + Int. IVa + 5a]	**TS_{Add-C}**	-1700.924578	-11.85	0.463948	**-1**	0.411315	-1702.567762	-7.56	-5.89	6.77
**TS_{Add-C} [X + Int. IVa + 12a] + H_2O**		-1777.272707	**-18.43**	0.485234		0.416135	-1779.003792	**-12.73**	**-12.45**	**2.05**
Cpl [X + 11a]	**X+11a**	-1700.960193	-34.20	0.466556	**-1**	0.411402	-1702.603894	-30.23	-26.92	-15.85
**[X + 11a] + H_2O**			**-40.78**					**-35.40**	**-33.48**	**-20.57**
**11a + H_2O vs [8a 9a] + 12a**		-1016.967738	**-24.73**	0.370270	**0**	0.309407	-1018.076862	**-21.10**	**-20.39**	**-17.02**

Absolute energy values in a.u., relative energy values in kcal mol^{-1}. [a] def2-SVP data; [b] def2-TZVP data.

2.4. The aldol condensation between **10a** and **12a**.

**Table S-2.4**	E DZ /au[a]	ΔE	ZPE/au	Δn	δG^{273K} /au	E TZ /au[b]	ΔE	ΔE^{0K}	ΔG^{273K}
Model Cat	-760.315004		0.115649		0.086978	-760.940271			
Cpl [10a + 12a]	-768.918632		0.278959		0.238009	-769.761919			
**X + [10a + 12a]**	-1529.233636	**0.00**	0.394608	**0**	0.324987	-1530.702190	**0.00**	**0.00**	**0.00**
**$TS_{AldCond}$ X + [10a + 12a]**	-1529.241520	**-4.95**	0.395528	**-4**	0.346954	-1530.706867	**-2.93**	**-2.36**	**9.16**

Absolute energy values in a.u., relative energy values in kcal mol^{-1}. [a] def2-SVP data; [b] def2-TZVP data.

2.5. The formation of the imine from **9a** and **8e** (Table S-2.5a) or **8b** (Table S-2.5b).

**Table S-2.5a**		E DZ /au[a]	ΔE	ZPE/au	Δn	δG^{273K} /au	E TZ /au[b]	ΔE	ΔE^{0K}	ΔG^{273K}
Model Cat	**X**	-760.315004		0.115649		0.086978	-760.940271			
Cpl [8e + 9a]	**8e+9a**	-836.777538		0.233099		0.192550	-837.696400			
**X + Cpl [8e + 9a]**	**X+8e+9a**	-1981.535828	**0.00**	0.488009	**0**	0.389599	-1983.503437	**0.00**	**0.00**	**0.00**
**TS_{Add} [X + 2d + 3a]**	**TS_{Add-N}**	-1597.105159	**-7.92**	0.351598		0.299646	-1598.646358	**-6.08**	**-4.29**	**4.86**
**Cpl [X + Int. Ie]**	**X+Ie**	-1597.124295	**-19.93**	0.354041	**-1**	0.304022	-1598.663383	**-16.76**	**-13.44**	**-3.08**
**TS_{De-H2O} [X + Int. IIe]**	**TS_{De-H2O}**	-1597.100131	**-4.76**	0.351658	**-1**	0.303057	-1598.639316	**-1.66**	**0.17**	**11.42**
Cpl [X + Int. IVd]	**X+IVe**	-1520.752764		0.325483		0.267901	-1522.207665			
H_2O		-76.348129		0.021286		0.004820	-76.436030			
**[X + Int. IVe] + H_2O**		-1520.752764	**-5.24**	0.325483	**0**	0.267901	-1522.207665	**-4.41**	**-5.65**	**-8.68**

Absolute energy values in a.u., relative energy values in kcal mol^{-1}. [a] def2-SVP data; [b] def2-TZVP data.

**Table S-2.5b**		E DZ /au[a]	ΔE	ZPE/au	Δn	δG^{273K} /au	E TZ /au[b]	ΔE	ΔE^{0K}	ΔG^{273K}
Model Cat		-760.315004		0.115649		0.086978	-760.940271			
Cpl [8b + 9a]		-836.780170		0.233309		0.193387	-837.698345			
**X + Cpl [8b + 9a]**		-1981.538459	**0.00**	0.488219	**0**	0.390436	-1983.505382	**0.00**	**0.00**	**0.00**
**TS_{Add} [X + 2b + 3a]**		-1597.105316	**-6.36**	0.351699	**-1**	0.301043	-1598.646461	**-4.92**	**-3.20**	**6.37**
**Cpl [X + Int. Ib]**	**X+Ib**	-1597.124030	**-18.11**	0.353854	**-1**	0.305976	-1598.663086	**-15.35**	**-12.28**	**-0.97**
**TS_{De-H2O} [X + Int. IIb]**		-1597.100836	**-3.55**	0.351992	**-1**	0.304328	-1598.639690	**-0.67**	**1.23**	**12.68**
Cpl [X + Int. IVb]	**X+IVb**	-1520.753075		0.325583		0.275954	-1522.207984			
H_2O		-76.348129		0.021286		0.004820	-76.436030			
**[X + Int. IVe] + H_2O**		-1981.544490	**-3.78**	0.486130	**0**	0.390845	-1983.510780	**-3.39**	**-4.70**	**-3.13**

Absolute energy values in a.u., relative energy values in kcal mol^{-1}. [a] def2-SVP data; [b] def2-TZVP data.

2.6. The enolization of **10b** to **12b**.

**Table S-2.6**		E DZ /au[a]	ΔE	ZPE/au	Δn	δG^{273K} /au	E TZ /au[b]	ΔE	ΔE^{0K}	ΔG^{273K}
Model Cat		-760.315004		0.115649		0.086978	-760.940271			
4-NitroAcetophenone	**10b**	-588.754214		0.141961		0.108260	-589.405611			
**X + 10b**		-1349.069218	**0.00**	0.257610	**0**	0.195238	-1350.345882	**0.00**	**0.00**	**0.00**
TS_{Enol} **[X + 10b]**	TS_{Enol}	-1349.049086	**12.63**	0.254085	**-1**	0.210706	-1350.323528	**14.03**	**11.82**	**22.05**
Enol-Acetophenone	**12b**	-588.736427		0.142578		0.109560	-589.387696			
**X + 12b**		-1349.051431	**11.16**	0.258227	**0**	0.196538	-1350.327968	**11.24**	**11.63**	**12.06**

Absolute energy values in a.u., relative energy values in kcal mol^{-1}. [a] def2-SVP data; [b] def2-TZVP data.

2.7. The addition of the enol **12e** to **IVa** (*Mannich*, Table S-2.7a) or to **10e** (aldol condensation, Table S-27b).

**Table S-2.7a**		E DZ /au[a]	ΔE	ZPE/au	Δn	δG^{273K} /au	E TZ /au[b]	ΔE	ΔE^{0K}	ΔG^{273K}
Cpl [X * IVa]		-1316.463566		0.321981		0.276257	-1317.690090			
Enol-Acetone	**12e**	-192.910116		0.084587		0.060418	-193.130280			
**[X * IVa] + 12e**		-1509.373683	**0.00**	0.406568		0.336675	-1510.820370	**0.00**	**0.00**	
TS_{Add} **[X + Int. IVa + 12e]**		-1509.389525	**-9.94**	0.409939	**0**	0.361242	-1510.830243	**-6.20**	**-4.08**	**7.53**

**Table S-2.7b**	E DZ /au[a]	ΔE	ZPE/au	Δn	δG^{273K} /au	E TZ /au[b]	ΔE	ΔE^{0K}	ΔG^{273K}
Cpl [10e * 12e]	-385.848883		0.169680		0.135521	-386.286570			
**X + [10e * 12e]**	-1146.165282	**0.00**	0.285355		0.222557	-1147.228269			
$TS_{AldCond}$ **X + [10e * 12e]**	-1146.180514	**-9.56**	0.287763	**0**	0.247022	-1147.239054	**-6.77**	**-5.26**	**6.90**

Absolute energy values in a.u., relative energy values in kcal mol^{-1}. [a] def2-SVP data; [b] def2-TZVP data.

2.8. The addition of the enol **12a** to **IVa** catalized by CPA **7**.

**Table S-2.8**		E DZ /au[a]	ΔE	ZPE/au	$\Delta E^{0K,b}$	Δn	δG^{273K} /au	$\Delta G^{273K,b}$
Catalyst	**7**	-1258.995209		0.277721		**f.6**	0.236664	
N-PhenylBenzaldimine	**IVa**	-556.125832		0.204743			0.170571	
Enol-Acetophenone	**12a**	-384.443286		0.139261			0.110071	
**Cat + IVa + 5a**		-2199.564327	**0.00**	0.621725	**0.00**	**0**	0.517306	**0.00**
TS_{Add} **(R) (a)**		-2199.610220	**-28.80**	0.626721	*4.34*	**-2**	0.567410	**1.27**
TS_{Add} **(R) (b)**		-2199.606734	**-26.61**	0.626092	*6.13*	**-2**	0.565318	**2.14**
TS_{Add} **(R) (c)**		-2199.603692	**-24.70**	0.625974	*7.96*	**-2**	0.561545	**1.68**
TS_{Add} **(R) (d)**		-2199.607189	**-26.90**	0.625992	*5.78*	**-2**	0.562856	**0.31**
TS_{Add} **(R) (e)**		-2199.607607	**-27.16**	0.626062	*5.56*	**-2**	0.562823	**0.03**
TS_{Add} **(S) (a)**		-2199.607386	**-27.02**	0.625872	*5.58*	**-2**	0.564448	**1.19**
TS_{Add} **(S) (b)**		-2199.607653	**-27.19**	0.626766	*5.98*	**-2**	0.565895	**1.93**
TS_{Add} **(S) (c)**		-2199.604244	**-25.05**	0.626242	*7.79*	**-2**	0.562292	**1.80**
TS_{Add} **(S) (d)**		-2199.605167	**-25.63**	0.625778	*6.92*	**-2**	0.560530	**0.12**
TS_{Add} **(S) (e)**		-2199.607615	**-27.16**	0.626610	*5.90*	**-2**	0.565199	**1.51**

Absolute energy values in a.u., relative energy values in kcal mol^{-1}. [a] def2-SVP data; [b] an arbitrary value of 30 have been added to all energy values to make the comparison easier.

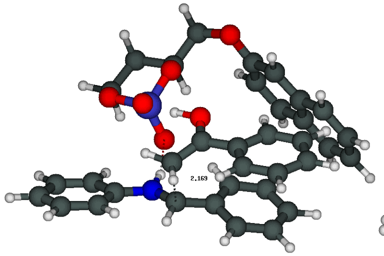

R (a)

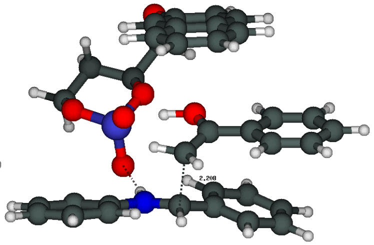

R (b)

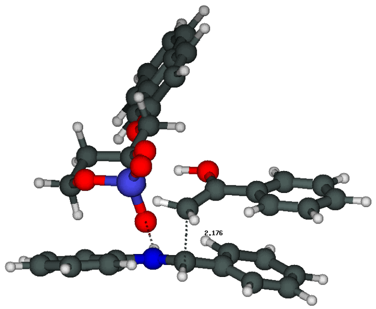

R (c)

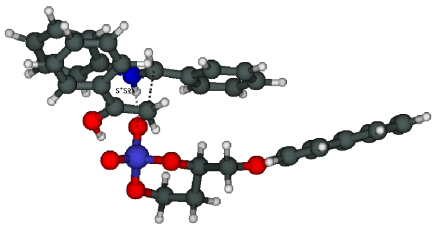

R (d)

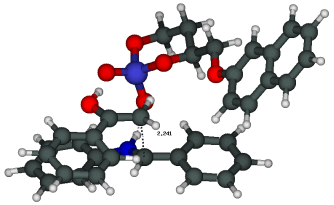

R (e)

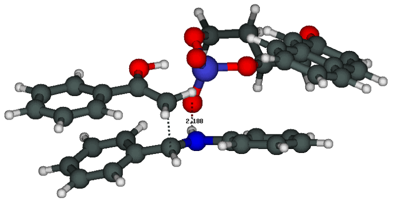

S (a)

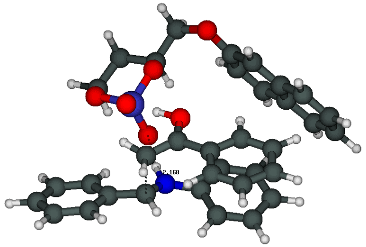

S (b)

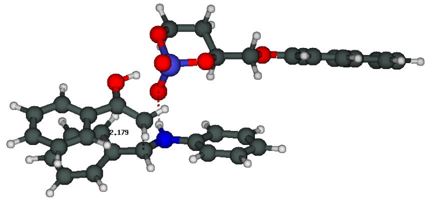

S (c)

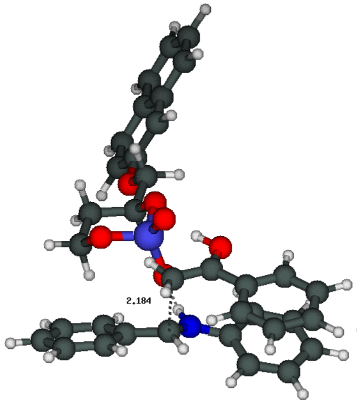

S (d)

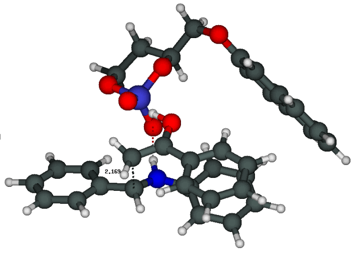

S (e)

3. Pictures and Cartesian coordinates.

3.1. The specie involved in the *Mannich* reaction among **8a**, **9a** and **10a** (and **12a**) to **11a**.

Model catalyst **X**

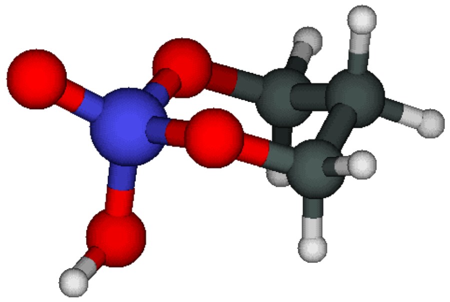

1	15	0	-0.002751	-0.000141	0.000695
2	8	0	-0.002481	0.000076	1.602724
3	8	0	1.552452	0.008328	-0.424625
4	6	0	0.603736	1.122448	2.283622
5	6	0	-0.002315	2.425658	1.796751
6	1	0	0.412908	0.964376	3.350760
7	1	0	1.690552	1.096009	2.109980
8	6	0	0.163944	2.594320	0.298272
9	1	0	-1.071253	2.454207	2.055184
10	1	0	0.495712	3.263021	2.305266
11	8	0	-0.434689	1.479799	-0.406194
12	1	0	-0.353147	3.488631	-0.066092
13	1	0	1.225575	2.657372	0.014878
14	1	0	1.972821	-0.864466	-0.393898
15	8	0	-0.824001	-1.078746	-0.579890

Benzaldehyde **8a**

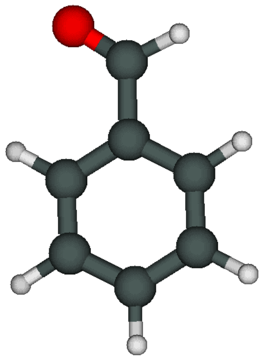

1	6	0	0.024734	0.000000	-0.015105
2	6	0	0.006983	0.000000	1.383741
3	6	0	1.206176	-0.000000	2.094970
4	6	0	2.419208	-0.000000	1.405210
5	6	0	2.439290	0.000000	0.005995
6	6	0	1.244385	0.000000	-0.705694
7	1	0	-0.951275	0.000000	1.909357
8	1	0	1.196217	-0.000000	3.186079
9	1	0	3.358996	-0.000000	1.960667

10	1	0	3.392194	-0.000000	-0.525686
11	1	0	1.239097	0.000000	-1.797557
12	6	0	-1.262337	0.000000	-0.748668
13	8	0	-1.360856	-0.000000	-1.954180
14	1	0	-2.170796	-0.000000	-0.105129

Aniline **9a**

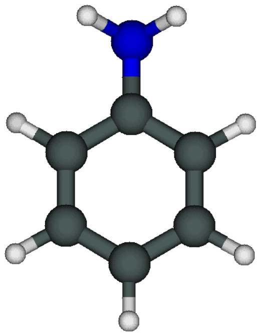

1	6	0	0.008540	-0.035809	-0.041807
2	6	0	0.007430	-0.059785	1.363380
3	6	0	1.206912	-0.036229	2.070662
4	6	0	2.431226	0.014572	1.400717
5	6	0	2.435718	0.040236	0.004563
6	6	0	1.242174	0.017076	-0.712783
7	1	0	-0.947157	-0.098720	1.893309
8	1	0	1.182425	-0.058117	3.162388
9	1	0	3.368250	0.032111	1.959015
10	1	0	3.382705	0.078846	-0.538210
11	1	0	1.252445	0.038423	-1.805033
12	7	0	-1.187382	0.001742	-0.751631
13	1	0	-1.125385	-0.352793	-1.699268
14	1	0	-1.978160	-0.404582	-0.264525

Acetophenone **10a**

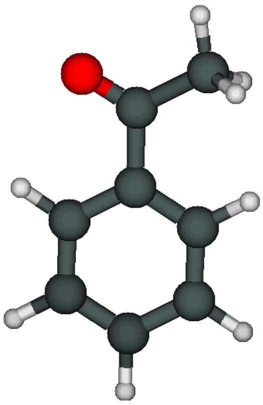

1	6	0	0.018515	0.017058	0.011477
2	6	0	0.026471	-0.039016	1.411056
3	6	0	1.236490	-0.072287	2.103743
4	6	0	2.442987	-0.050372	1.403892

5	6	0	2.441372	0.005539	0.007500
6	6	0	1.234704	0.038941	-0.684705
7	1	0	-0.910515	-0.056545	1.969288
8	1	0	1.236304	-0.115680	3.194095
9	1	0	3.389057	-0.076652	1.947855
10	1	0	3.385006	0.022837	-0.540389
11	1	0	1.216206	0.082620	-1.774833
12	6	0	-1.263486	0.054595	-0.771636
13	8	0	-1.238797	0.105634	-1.984950
14	6	0	-2.565033	0.026497	-0.015603
15	1	0	-2.632497	-0.887909	0.592120
16	1	0	-2.624290	0.882043	0.673348
17	1	0	-3.400634	0.062698	-0.723258

Product **11a**

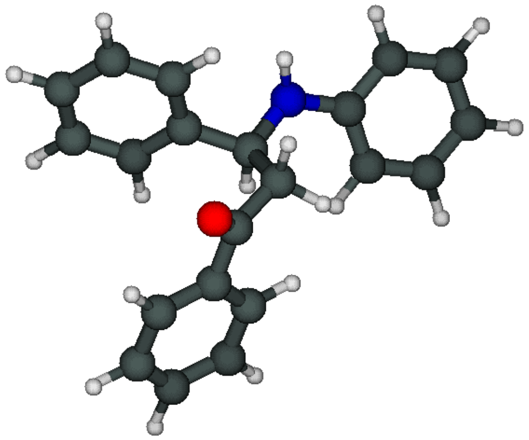

1	6	0	-0.100031	-0.108305	-0.193075
2	6	0	-0.200190	-0.022825	1.317799
3	7	0	1.286212	-0.007813	-0.612096
4	6	0	-1.016436	0.942713	1.914902
5	6	0	-1.152920	0.997367	3.303567
6	6	0	-0.469113	0.087681	4.109018
7	6	0	0.348289	-0.879891	3.520621
8	6	0	0.476811	-0.938200	2.133706
9	1	0	-1.552766	1.656767	1.284248
10	1	0	-1.793846	1.756523	3.755668
11	1	0	-0.573442	0.129686	5.194710
12	1	0	0.883001	-1.597973	4.145065
13	1	0	1.106182	-1.711537	1.684790
14	1	0	-0.640866	0.757286	-0.600546
15	6	0	1.649510	0.143806	-1.941079
16	6	0	2.956132	-0.203602	-2.339706
17	6	0	3.372320	-0.014096	-3.651822
18	6	0	2.502465	0.517547	-4.608967
19	6	0	1.207561	0.857969	-4.221582
20	6	0	0.776537	0.676271	-2.907137
21	1	0	3.639949	-0.621167	-1.596703
22	1	0	4.390567	-0.291745	-3.932592
23	1	0	2.831100	0.661952	-5.638974
24	1	0	0.510774	1.274603	-4.952073
25	1	0	-0.240658	0.957883	-2.633814
26	1	0	1.925229	-0.590785	-0.081502

27	6	0	-0.761134	-1.410373	-0.720261
28	6	0	-2.131056	-1.656775	-0.128768
29	8	0	-2.347384	-2.631316	0.562713
30	6	0	-3.211304	-0.642938	-0.365532
31	1	0	-0.827155	-1.358685	-1.818781
32	1	0	-0.126868	-2.266247	-0.451404
33	6	0	-4.281944	-0.585788	0.537267
34	6	0	-5.293436	0.354640	0.366328
35	6	0	-5.250693	1.236027	-0.717440
36	6	0	-4.194406	1.176297	-1.626799
37	6	0	-3.172274	0.244019	-1.448872
38	1	0	-4.300098	-1.280870	1.378542
39	1	0	-6.118413	0.403836	1.078969
40	1	0	-6.045166	1.972239	-0.852859
41	1	0	-4.164515	1.858423	-2.477938
42	1	0	-2.351722	0.201937	-2.167303

Enol **12a**

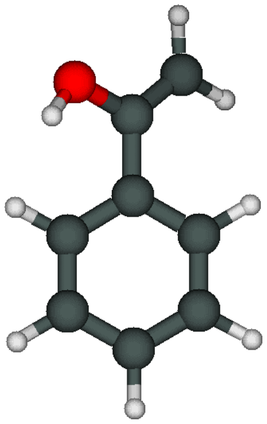

1	6	0	-0.062586	-0.085801	0.033135
2	6	0	0.001299	-0.220875	1.428153
3	6	0	1.226991	-0.168419	2.086467
4	6	0	2.407967	0.010281	1.362532
5	6	0	2.355199	0.136307	-0.025001
6	6	0	1.128528	0.086838	-0.687320
7	1	0	-0.916128	-0.385444	1.996005
8	1	0	1.262577	-0.280314	3.171576
9	1	0	3.368046	0.047353	1.880163
10	1	0	3.272628	0.280257	-0.598281
11	1	0	1.103026	0.209730	-1.772859
12	6	0	-1.370457	-0.128128	-0.672901
13	8	0	-1.359388	-0.622399	-1.941982
14	6	0	-2.519916	0.318473	-0.152271
15	1	0	-3.447392	0.241777	-0.721263
16	1	0	-2.536766	0.788929	0.829296
17	1	0	-0.554858	-1.132533	-2.101434

TS$_{Enol}$

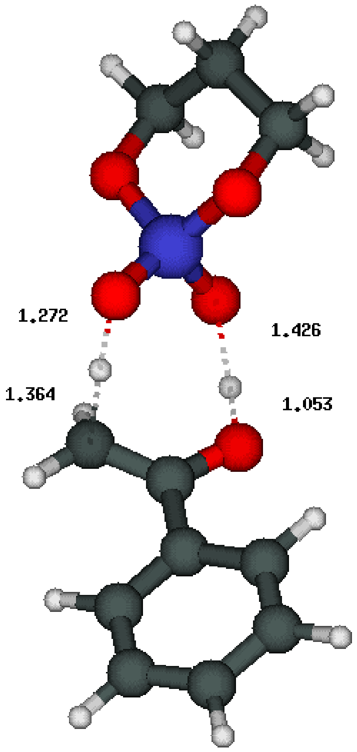

1	15	0	0.035945	-0.003186	-0.002745
2	8	0	0.050952	-0.008372	1.606037
3	8	0	1.447174	0.025099	-0.571475
4	6	0	0.533953	1.190330	2.248154
5	6	0	-0.279144	2.393582	1.802579
6	1	0	0.436276	1.017789	3.325998
7	1	0	1.601161	1.318781	2.007674
8	6	0	-0.247787	2.561827	0.293287
9	1	0	-1.320685	2.275565	2.136504
10	1	0	0.132023	3.297923	2.273040
11	8	0	-0.731167	1.366435	-0.354341
12	1	0	-0.908609	3.372978	-0.032847
13	1	0	0.772533	2.773712	-0.063751
14	1	0	1.992373	-1.223644	-0.993545
15	8	0	-0.834172	-1.179960	-0.457227
16	8	0	2.273287	-2.219174	-1.189863
17	6	0	1.403345	-2.859484	-1.899184
18	6	0	0.360964	-2.191932	-2.559984
19	1	0	-0.334188	-1.778715	-1.462174
20	1	0	0.590833	-1.178947	-2.907101
21	1	0	-0.292855	-2.777273	-3.205906
22	6	0	1.531153	-4.334189	-1.859112
23	6	0	0.928163	-5.140322	-2.835866
24	6	0	1.064887	-6.524588	-2.778945
25	6	0	1.798858	-7.113367	-1.747836
26	6	0	2.403653	-6.316112	-0.773975
27	6	0	2.276019	-4.931614	-0.830220
28	1	0	0.369240	-4.687556	-3.655175
29	1	0	0.600375	-7.146365	-3.545600
30	1	0	1.902760	-8.199068	-1.704546

31	1	0	2.976496	-6.776290	0.032414
32	1	0	2.742865	-4.302454	-0.072193

8a+9a

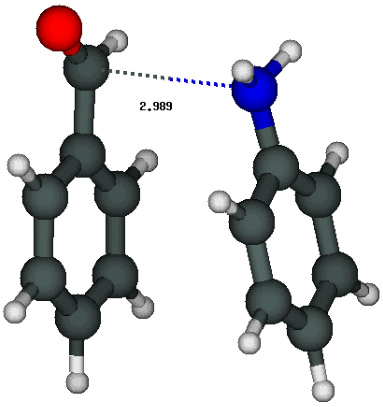

1	6	0	0.007045	0.026731	0.004000
2	6	0	0.060885	0.084469	1.400473
3	6	0	1.293853	0.122940	2.050232
4	6	0	2.471747	0.106688	1.300663
5	6	0	2.420510	0.051496	-0.096353
6	6	0	1.190052	0.009636	-0.745706
7	1	0	-0.869734	0.088151	1.974595
8	1	0	1.338353	0.161691	3.140194
9	1	0	3.438363	0.136310	1.807475
10	1	0	3.345380	0.039079	-0.675602
11	1	0	1.129531	-0.038462	-1.834993
12	6	0	-1.316077	-0.041046	-0.661405
13	8	0	-1.473891	-0.106864	-1.859710
14	1	0	-2.189299	-0.001408	0.026247
15	7	0	-1.432076	-2.868792	0.299322
16	6	0	-0.364910	-3.141438	1.143505
17	6	0	-0.542520	-3.159028	2.538038
18	6	0	0.546916	-3.340105	3.386705
19	6	0	1.835018	-3.501841	2.871351
20	6	0	2.013844	-3.492695	1.485696
21	6	0	0.931247	-3.314993	0.627516
22	1	0	-1.546424	-3.022802	2.947309
23	1	0	0.385420	-3.349974	4.466879
24	1	0	2.685999	-3.642653	3.539276
25	1	0	3.013006	-3.621317	1.063080
26	1	0	1.078361	-3.303020	-0.455247
27	1	0	-1.319219	-3.188176	-0.656112
28	1	0	-2.345819	-3.110146	0.666205

X+8a+9a

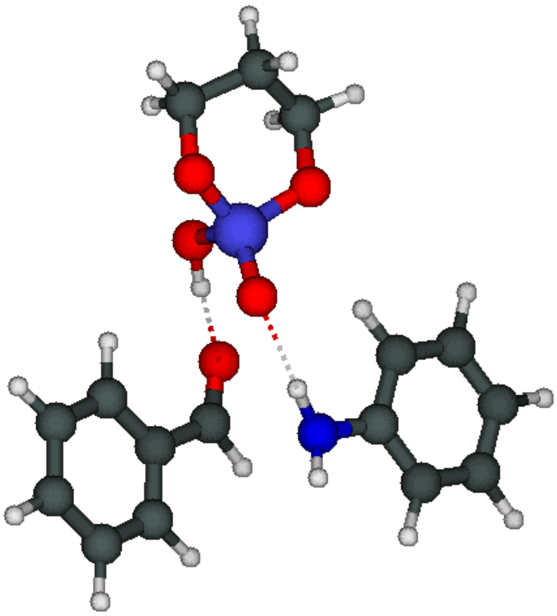

1	15	0	0.266861	-0.095759	-0.305254
2	8	0	-0.400562	-0.216718	1.150182
3	8	0	1.827533	-0.294853	-0.091475
4	6	0	-0.003028	0.746510	2.150080
5	6	0	-0.233223	2.158116	1.641351
6	1	0	-0.614185	0.533455	3.034122
7	1	0	1.055471	0.578847	2.402378
8	6	0	0.521472	2.417659	0.350279
9	1	0	-1.309171	2.318499	1.477803
10	1	0	0.108633	2.873200	2.402745
11	8	0	0.139195	1.457445	-0.661508
12	1	0	0.280607	3.402487	-0.064833
13	1	0	1.609883	2.353565	0.502129
14	1	0	2.149923	-1.231781	-0.254371
15	8	0	-0.383568	-0.993647	-1.289445
16	8	0	2.544292	-2.747263	-0.449124
17	6	0	2.510008	-3.471712	-1.431161
18	6	0	2.549039	-3.005183	-2.829874
19	6	0	2.471019	-3.964555	-3.847140
20	6	0	2.497430	-3.568459	-5.181915
21	6	0	2.605063	-2.212917	-5.498711
22	6	0	2.691371	-1.253216	-4.485468
23	6	0	2.664238	-1.645569	-3.150605
24	1	0	2.383760	-5.021211	-3.581624
25	1	0	2.433604	-4.313700	-5.976138
26	1	0	2.626015	-1.899808	-6.544174
27	1	0	2.781418	-0.196235	-4.740191
28	1	0	2.737488	-0.900204	-2.357253
29	1	0	2.505744	-4.570159	-1.293352
30	7	0	-0.204080	-3.938873	-1.553800
31	6	0	-0.423463	-4.601415	-0.360765
32	6	0	-0.400442	-3.901032	0.860023
33	6	0	-0.555387	-4.580045	2.066087
34	6	0	-0.734115	-5.965057	2.091366
35	6	0	-0.758629	-6.663337	0.881758
36	6	0	-0.603123	-5.996676	-0.330813

37	1	0	-0.273114	-2.816782	0.847531
38	1	0	-0.537892	-4.013670	2.999815
39	1	0	-0.856978	-6.491792	3.038730
40	1	0	-0.900467	-7.746385	0.879684
41	1	0	-0.621642	-6.549454	-1.273120
42	1	0	-0.347037	-2.929749	-1.546148
43	1	0	-0.545660	-4.396776	-2.390133

TS_{Add-N}

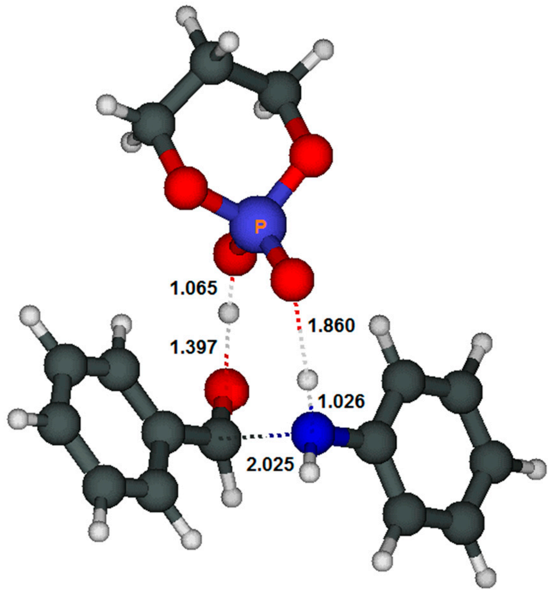

1	15	0	-0.017163	-0.215904	0.016641
2	8	0	-0.547572	0.365432	1.416153
3	8	0	1.486199	-0.583589	0.292399
4	6	0	0.079782	1.565106	1.916365
5	6	0	-0.006059	2.678983	0.887568
6	1	0	-0.456495	1.826936	2.835571
7	1	0	1.126602	1.337138	2.170443
8	6	0	0.621125	2.268560	-0.433487
9	1	0	-1.060390	2.947274	0.724057
10	1	0	0.517707	3.564414	1.274613
11	8	0	-0.012469	1.077105	-0.939868
12	1	0	0.478283	3.038580	-1.200342
13	1	0	1.700304	2.078560	-0.321116
14	1	0	1.898401	-1.401692	-0.230704
15	8	0	-0.890198	-1.294278	-0.519909
16	8	0	2.463762	-2.562570	-0.813561
17	6	0	1.997990	-3.106620	-1.836934
18	6	0	1.514300	-2.315031	-3.009949
19	6	0	0.970838	-2.981320	-4.115467
20	6	0	0.532313	-2.260984	-5.222898
21	6	0	0.642844	-0.867690	-5.235300
22	6	0	1.201056	-0.201791	-4.143914
23	6	0	1.639446	-0.922796	-3.032754
24	1	0	0.891429	-4.071869	-4.100484
25	1	0	0.105948	-2.784153	-6.080404
26	1	0	0.300976	-0.301379	-6.103478
27	1	0	1.300576	0.885025	-4.157038

28	1	0	2.100463	-0.402467	-2.192013
29	1	0	2.322504	-4.135507	-2.074395
30	7	0	0.152839	-3.870225	-1.243762
31	6	0	0.381652	-4.663180	-0.108946
32	6	0	0.474451	-4.063410	1.153317
33	6	0	0.785875	-4.839529	2.268640
34	6	0	1.012427	-6.210385	2.137652
35	6	0	0.926039	-6.804072	0.876277
36	6	0	0.617871	-6.037528	-0.244958
37	1	0	0.292285	-2.991781	1.249864
38	1	0	0.850970	-4.364861	3.249368
39	1	0	1.254161	-6.814104	3.013729
40	1	0	1.101369	-7.875559	0.762523
41	1	0	0.549625	-6.496247	-1.234031
42	1	0	-0.345957	-2.992477	-1.051720
43	1	0	-0.257102	-4.375183	-2.026126

X-Ia

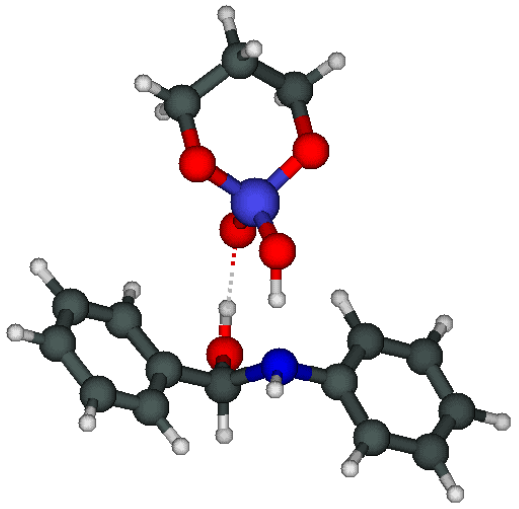

1	15	0	-0.057049	-0.253408	-0.019736
2	8	0	-0.500633	0.089896	1.487640
3	8	0	1.418458	-0.502122	-0.145495
4	6	0	-0.096786	1.377872	1.998324
5	6	0	-0.623329	2.487670	1.104812
6	1	0	-0.512083	1.446711	3.010011
7	1	0	1.002218	1.407603	2.067167
8	6	0	-0.151045	2.321187	-0.329185
9	1	0	-1.723112	2.487350	1.130251
10	1	0	-0.270716	3.455272	1.488819
11	8	0	-0.554236	1.034496	-0.843194
12	1	0	-0.605800	3.067389	-0.990378
13	1	0	0.944664	2.408636	-0.401911
14	1	0	2.060626	-1.802114	-1.034707
15	8	0	-0.984735	-1.415983	-0.467297
16	8	0	2.372478	-2.609023	-1.506111
17	6	0	1.350093	-3.179274	-2.228272
18	6	0	0.822172	-2.323002	-3.371005
19	6	0	-0.011637	-2.906576	-4.332508
20	6	0	-0.537714	-2.141114	-5.371366

21	6	0	-0.230722	-0.781588	-5.460427
22	6	0	0.607034	-0.198028	-4.510848
23	6	0	1.133946	-0.965029	-3.468609
24	1	0	-0.245746	-3.973531	-4.269564
25	1	0	-1.184498	-2.607494	-6.116627
26	1	0	-0.639426	-0.179975	-6.274246
27	1	0	0.856147	0.862650	-4.578523
28	1	0	1.793262	-0.501643	-2.733305
29	1	0	1.733890	-4.129561	-2.634541
30	7	0	0.185173	-3.504180	-1.336456
31	6	0	0.490077	-4.403187	-0.257367
32	6	0	1.074764	-3.903976	0.909744
33	6	0	1.364471	-4.772278	1.961292
34	6	0	1.065449	-6.131882	1.856452
35	6	0	0.476043	-6.624542	0.691841
36	6	0	0.192954	-5.763129	-0.367830
37	1	0	1.298480	-2.838432	0.987448
38	1	0	1.821523	-4.380275	2.871377
39	1	0	1.288862	-6.806693	2.684434
40	1	0	0.236547	-7.685704	0.603808
41	1	0	-0.264462	-6.139466	-1.285896
42	1	0	-0.466673	-2.294551	-0.838828
43	1	0	-0.533025	-3.932688	-1.923553

X-IIa

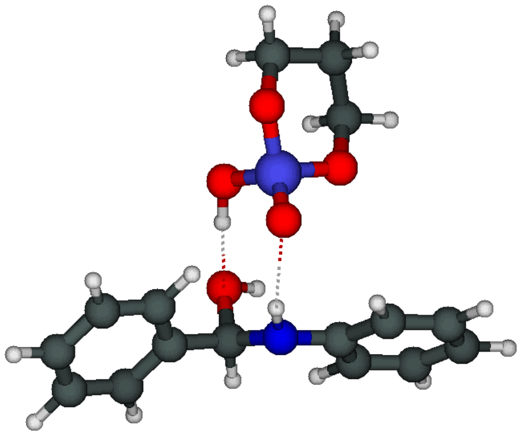

1	15	0	-0.065501	-0.016774	-0.128977
2	8	0	0.329582	-0.298281	1.399889
3	8	0	1.301737	0.026405	-0.940488
4	6	0	1.156035	0.679429	2.068679
5	6	0	0.522936	2.056166	1.979213
6	1	0	1.238898	0.342205	3.107850
7	1	0	2.157925	0.668966	1.611953
8	6	0	0.298761	2.478959	0.538644
9	1	0	-0.437103	2.054092	2.516366
10	1	0	1.185737	2.786444	2.464362
11	8	0	-0.527554	1.512143	-0.150534
12	1	0	-0.241457	3.430196	0.479542
13	1	0	1.251291	2.577167	-0.003883
14	1	0	1.765158	-0.853688	-1.043541
15	8	0	-1.099750	-0.957653	-0.624709
16	8	0	2.496364	-2.305775	-1.147802

17	6	0	1.655220	-3.388342	-1.622727
18	6	0	1.353331	-3.101225	-3.076429
19	6	0	2.084450	-3.762501	-4.066640
20	6	0	1.850568	-3.493175	-5.415403
21	6	0	0.885087	-2.555910	-5.782028
22	6	0	0.153674	-1.891359	-4.796272
23	6	0	0.384036	-2.161827	-3.447535
24	1	0	2.840095	-4.496611	-3.778075
25	1	0	2.423729	-4.018656	-6.181273
26	1	0	0.700189	-2.343979	-6.836684
27	1	0	-0.604732	-1.158598	-5.076870
28	1	0	-0.200557	-1.634314	-2.689556
29	1	0	2.249329	-4.308609	-1.561402
30	7	0	0.474606	-3.533591	-0.839072
31	6	0	0.532619	-3.952307	0.489796
32	6	0	-0.598720	-3.756914	1.304250
33	6	0	-0.598923	-4.178898	2.628162
34	6	0	0.525500	-4.801711	3.180181
35	6	0	1.648186	-4.993680	2.378796
36	6	0	1.663077	-4.573341	1.046682
37	1	0	-1.472626	-3.261442	0.876843
38	1	0	-1.489390	-4.015648	3.238677
39	1	0	0.522671	-5.131656	4.219816
40	1	0	2.537364	-5.476384	2.789362
41	1	0	2.560781	-4.747772	0.450823
42	1	0	-0.256523	-2.845766	-1.012166
43	1	0	2.872429	-2.549969	-0.289057

TS$_{De-H2O}$

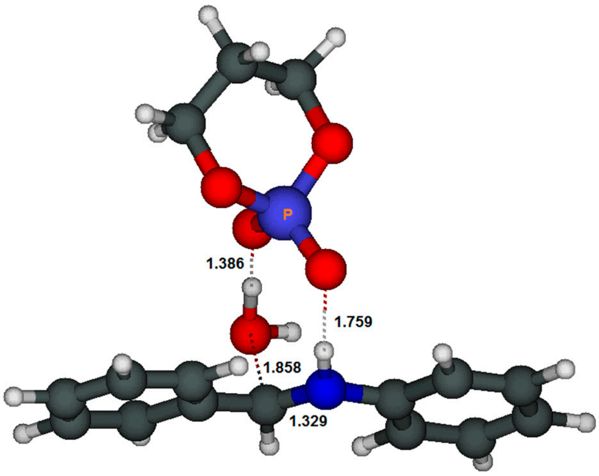

1	15	0	0.051082	-0.053325	0.071558
2	8	0	-0.038334	0.231315	1.671056
3	8	0	1.523437	-0.188045	-0.312199
4	6	0	0.604977	1.425335	2.138352
5	6	0	0.028073	2.648553	1.443208
6	1	0	0.434072	1.468344	3.221098
7	1	0	1.689619	1.347037	1.958407
8	6	0	0.151235	2.540781	-0.068490
9	1	0	-1.033057	2.754704	1.715286
10	1	0	0.562169	3.546317	1.786589
11	8	0	-0.492236	1.348678	-0.542233

12	1	0	-0.345706	3.381846	-0.567293
13	1	0	1.209328	2.526815	-0.376243
14	1	0	2.395086	-1.265671	-0.511851
15	8	0	-0.905135	-1.156722	-0.283882
16	8	0	3.023585	-2.094112	-0.704161
17	6	0	1.783476	-3.344865	-1.366902
18	6	0	1.610392	-2.793066	-2.731393
19	6	0	2.643556	-3.010398	-3.652147
20	6	0	2.539384	-2.522199	-4.951419
21	6	0	1.400461	-1.813196	-5.337618
22	6	0	0.368108	-1.597363	-4.423593
23	6	0	0.467138	-2.084595	-3.121360
24	1	0	3.532395	-3.563457	-3.341034
25	1	0	3.346757	-2.695843	-5.664341
26	1	0	1.315364	-1.430310	-6.356158
27	1	0	-0.524163	-1.046481	-4.724639
28	1	0	-0.347226	-1.902373	-2.418505
29	1	0	2.501503	-4.161422	-1.290127
30	7	0	0.766243	-3.369924	-0.512137
31	6	0	0.796542	-4.006395	0.747039
32	6	0	-0.323915	-3.845945	1.573209
33	6	0	-0.354044	-4.446014	2.828467
34	6	0	0.727671	-5.208015	3.274268
35	6	0	1.842197	-5.360400	2.450771
36	6	0	1.888112	-4.761454	1.191569
37	1	0	-1.159728	-3.240519	1.218158
38	1	0	-1.232117	-4.314449	3.462954
39	1	0	0.702695	-5.677931	4.258403
40	1	0	2.696658	-5.949258	2.788652
41	1	0	2.776730	-4.897177	0.574025
42	1	0	0.027418	-2.650729	-0.599730
43	1	0	3.349288	-2.405186	0.153470

X-IIIa

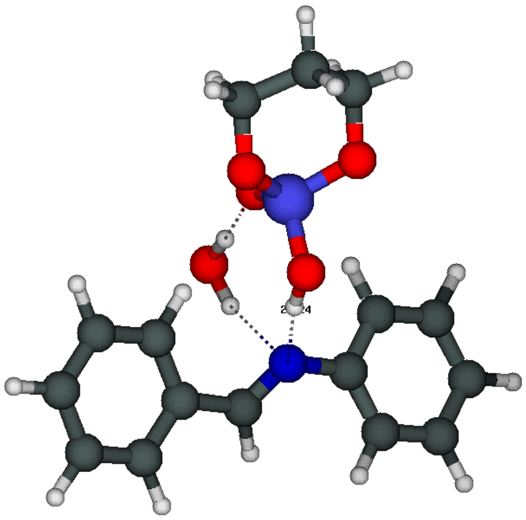

1	15	0	0.065118	-0.150714	0.218554
2	8	0	-0.318645	0.247611	1.727293
3	8	0	1.526414	-0.396621	0.018688
4	6	0	0.065791	1.569553	2.162639

5	6	0	-0.535705	2.620541	1.246008
6	1	0	-0.307142	1.670532	3.188015
7	1	0	1.165124	1.635414	2.179702
8	6	0	-0.120519	2.404766	-0.198820
9	1	0	-1.632706	2.587573	1.321051
10	1	0	-0.198551	3.615002	1.570845
11	8	0	-0.503803	1.083692	-0.638546
12	1	0	-0.627659	3.105832	-0.871169
13	1	0	0.967555	2.521813	-0.324112
14	1	0	2.661768	-1.682515	-0.520402
15	8	0	-0.882209	-1.340246	-0.136623
16	8	0	3.302230	-2.360568	-0.803116
17	6	0	0.302960	-4.162206	-1.835707
18	6	0	0.207158	-3.412933	-3.094065
19	6	0	-0.123715	-4.104520	-4.267639
20	6	0	-0.243412	-3.417155	-5.473584
21	6	0	-0.009916	-2.042002	-5.516687
22	6	0	0.350049	-1.352697	-4.354906
23	6	0	0.459310	-2.032395	-3.146294
24	1	0	-0.297532	-5.182075	-4.225660
25	1	0	-0.512842	-3.956279	-6.383119
26	1	0	-0.096079	-1.504574	-6.462786
27	1	0	0.555925	-0.281891	-4.394215
28	1	0	0.778961	-1.504462	-2.246055
29	1	0	0.457778	-5.248721	-1.915511
30	7	0	0.228349	-3.598855	-0.690384
31	6	0	0.337910	-4.344028	0.509123
32	6	0	0.958447	-3.714282	1.595734
33	6	0	1.096688	-4.392520	2.804957
34	6	0	0.598721	-5.689058	2.942103
35	6	0	-0.041869	-6.304859	1.864991
36	6	0	-0.177323	-5.638620	0.648847
37	1	0	1.343713	-2.698710	1.474112
38	1	0	1.592284	-3.902281	3.644338
39	1	0	0.699588	-6.216497	3.891959
40	1	0	-0.449957	-7.311164	1.974306
41	1	0	-0.702351	-6.110136	-0.183421
42	1	0	-0.378262	-2.214147	-0.415100
43	1	0	2.819775	-3.191237	-0.747051

X-IVa

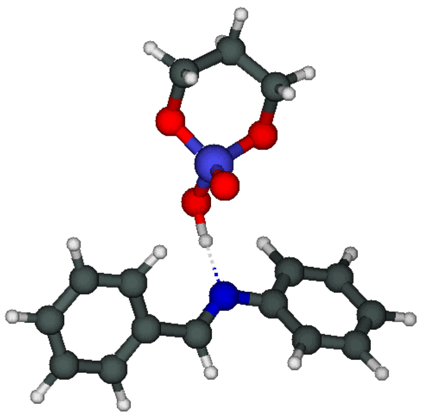

1	15	0	-0.024379	-0.006710	0.030137

2	8	0	0.032893	0.091132	1.638086
3	6	0	1.348884	0.095651	2.226688
4	6	0	2.171999	1.248945	1.677710
5	6	0	2.274533	1.188972	0.162962
6	8	0	0.958960	1.190150	-0.424939
7	1	0	1.197922	0.191561	3.308061
8	1	0	1.836205	-0.871791	2.025196
9	1	0	1.709939	2.202133	1.974846
10	1	0	3.181917	1.206772	2.109717
11	1	0	2.789636	2.069336	-0.238454
12	1	0	2.815612	0.286698	-0.164589
13	8	0	0.354798	-1.337203	-0.520866
14	8	0	-1.464899	0.506884	-0.303954
15	1	0	-1.894143	0.053922	-1.144460
16	7	0	-2.510881	-0.440048	-2.466657
17	6	0	-3.434976	-1.315226	-2.588223
18	6	0	-3.853765	-2.196805	-1.491633
19	6	0	-5.133989	-2.766670	-1.538341
20	6	0	-5.572493	-3.587157	-0.501473
21	6	0	-4.726623	-3.859165	0.574890
22	6	0	-3.438838	-3.316937	0.611587
23	6	0	-2.998883	-2.488612	-0.416553
24	1	0	-5.786498	-2.553793	-2.388180
25	1	0	-6.573692	-4.019576	-0.534915
26	1	0	-5.067239	-4.507217	1.384537
27	1	0	-2.770688	-3.549582	1.442364
28	1	0	-1.979597	-2.096486	-0.409146
29	1	0	-3.972405	-1.421452	-3.542788
30	6	0	-2.088560	0.319350	-3.582640
31	6	0	-1.748896	1.663445	-3.378563
32	6	0	-1.322113	2.441547	-4.452250
33	6	0	-1.212129	1.883178	-5.727140
34	6	0	-1.528922	0.538292	-5.923928
35	6	0	-1.962903	-0.247075	-4.857711
36	1	0	-1.835213	2.091508	-2.378551
37	1	0	-1.071289	3.491102	-4.289760
38	1	0	-0.869262	2.493019	-6.564509
39	1	0	-1.425920	0.090704	-6.914017
40	1	0	-2.181373	-1.306305	-5.003635

X-IVa-12a

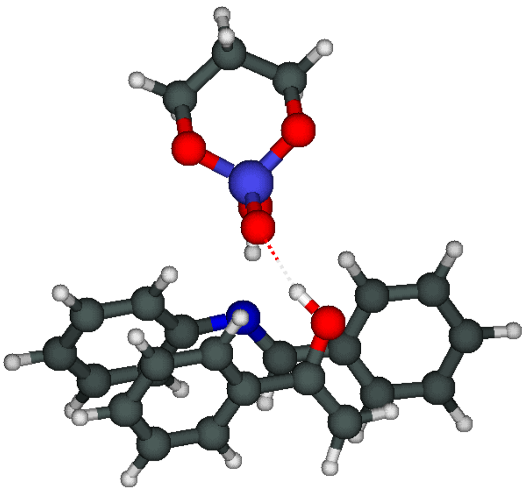

1	15	0	0.243203	0.192042	-0.045603
2	8	0	0.091159	0.179690	1.549176
3	6	0	1.304449	0.132138	2.332942
4	6	0	2.224657	1.279081	1.955182
5	6	0	2.559189	1.264089	0.474173
6	8	0	1.350177	1.323023	-0.312604
7	1	0	0.989650	0.198432	3.380243
8	1	0	1.791243	-0.841998	2.170694
9	1	0	1.744849	2.234858	2.212782
10	1	0	3.155375	1.195139	2.533703
11	1	0	3.149985	2.141246	0.187497
12	1	0	3.117704	0.356181	0.198176
13	8	0	0.904329	-1.185924	-0.411998
14	8	0	-1.032080	0.514054	-0.745445
15	1	0	0.524812	-1.666481	-1.278659
16	7	0	0.109123	-2.305240	-2.570255
17	6	0	-0.575516	-3.370702	-2.754556
18	6	0	-1.215452	-4.151445	-1.691303
19	6	0	-1.775225	-5.388539	-2.043926
20	6	0	-2.412395	-6.172638	-1.085511
21	6	0	-2.504508	-5.718226	0.230822
22	6	0	-1.954636	-4.483448	0.587403
23	6	0	-1.310844	-3.700777	-0.365777
24	1	0	-1.708101	-5.729905	-3.079482
25	1	0	-2.842561	-7.135271	-1.365963
26	1	0	-3.009294	-6.326277	0.983757
27	1	0	-2.033865	-4.126446	1.615312
28	1	0	-0.896351	-2.733773	-0.084180
29	1	0	-0.705374	-3.760996	-3.774958
30	6	0	0.749408	-1.667529	-3.662799
31	6	0	0.846801	-0.270586	-3.641667
32	6	0	1.478017	0.399053	-4.687612
33	6	0	2.025389	-0.316888	-5.753674
34	6	0	1.942876	-1.710294	-5.765038
35	6	0	1.313814	-2.389317	-4.723067
36	1	0	0.388169	0.287369	-2.823444
37	1	0	1.534596	1.488968	-4.670375
38	1	0	2.523701	0.209025	-6.569592
39	1	0	2.384984	-2.278114	-6.585616
40	1	0	1.282073	-3.480193	-4.720252
41	6	0	-3.931936	-2.652637	-2.994760
42	6	0	-3.220726	-1.542204	-2.735639
43	1	0	-4.203213	-2.919579	-4.014987
44	1	0	-4.274590	-3.283545	-2.172575
45	8	0	-2.984417	-1.181913	-1.459005
46	6	0	-2.707017	-0.641105	-3.804408
47	1	0	-2.246285	-0.542319	-1.347354
48	6	0	-2.596338	0.737377	-3.574161
49	6	0	-2.150210	1.590560	-4.584396
50	6	0	-1.798471	1.077419	-5.832815
51	6	0	-1.889702	-0.297408	-6.066028
52	6	0	-2.337104	-1.149987	-5.059318
53	1	0	-2.874964	1.149451	-2.602898
54	1	0	-2.080153	2.662902	-4.392656
55	1	0	-1.445175	1.744835	-6.621161
56	1	0	-1.600119	-0.709028	-7.034979
57	1	0	-2.388520	-2.225549	-5.241279

TS$_{Add\text{-}C}$

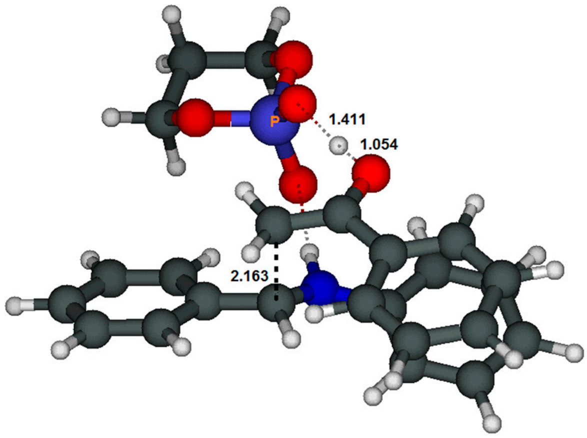

1	15	0	-0.004738	0.032981	-0.050840
2	8	0	-0.079457	0.041954	1.573772
3	6	0	1.184445	0.014413	2.253510
4	6	0	2.033836	1.209684	1.848623
5	6	0	2.238075	1.266896	0.342198
6	8	0	0.973236	1.299169	-0.333987
7	1	0	0.961578	0.033836	3.327366
8	1	0	1.700281	-0.931454	2.020884
9	1	0	1.540846	2.135181	2.182419
10	1	0	3.012577	1.143115	2.345288
11	1	0	2.771635	2.179604	0.049409
12	1	0	2.816148	0.396259	-0.009279
13	8	0	0.639991	-1.251667	-0.524572
14	8	0	-1.374768	0.401220	-0.591886
15	1	0	-0.030684	-1.699737	-2.107277
16	7	0	-0.479198	-1.640452	-3.040146
17	6	0	-1.537069	-2.399816	-3.324412
18	6	0	-1.782271	-3.647005	-2.578249
19	6	0	-2.575080	-4.630161	-3.189065
20	6	0	-2.839303	-5.829222	-2.532696
21	6	0	-2.313028	-6.055431	-1.258553
22	6	0	-1.526746	-5.078503	-0.645339
23	6	0	-1.260925	-3.873809	-1.295774
24	1	0	-2.982707	-4.447123	-4.186195
25	1	0	-3.453849	-6.590192	-3.016923
26	1	0	-2.516882	-6.995420	-0.742431
27	1	0	-1.115814	-5.252746	0.350351
28	1	0	-0.647754	-3.115934	-0.802069
29	1	0	-1.852978	-2.416366	-4.367503
30	6	0	-0.043544	-0.562225	-3.828063
31	6	0	1.141994	0.086896	-3.451060
32	6	0	1.613829	1.161008	-4.198373
33	6	0	0.915750	1.602459	-5.324843
34	6	0	-0.261878	0.955289	-5.696417
35	6	0	-0.749486	-0.121052	-4.955631
36	1	0	1.680864	-0.262527	-2.569530
37	1	0	2.537342	1.657684	-3.895267
38	1	0	1.287968	2.445622	-5.908487
39	1	0	-0.818872	1.291699	-6.572542
40	1	0	-1.678619	-0.600248	-5.263324
41	6	0	-3.245520	-1.103939	-2.946624
42	6	0	-3.675344	-1.640373	-1.743187

43	1	0	-2.640652	-0.196213	-2.912782
44	1	0	-3.827601	-1.278240	-3.849386
45	8	0	-3.124111	-1.329042	-0.608503
46	6	0	-4.691990	-2.715281	-1.660539
47	1	0	-2.381110	-0.582357	-0.648564
48	6	0	-4.786558	-3.471819	-0.482926
49	6	0	-5.681125	-4.536479	-0.400761
50	6	0	-6.499752	-4.848753	-1.487207
51	6	0	-6.427013	-4.086199	-2.655167
52	6	0	-5.528610	-3.026698	-2.744079
53	1	0	-4.139043	-3.228789	0.359497
54	1	0	-5.738413	-5.126361	0.515590
55	1	0	-7.200911	-5.682759	-1.422023
56	1	0	-7.075201	-4.317546	-3.501853
57	1	0	-5.491217	-2.437653	-3.660819

X+11a

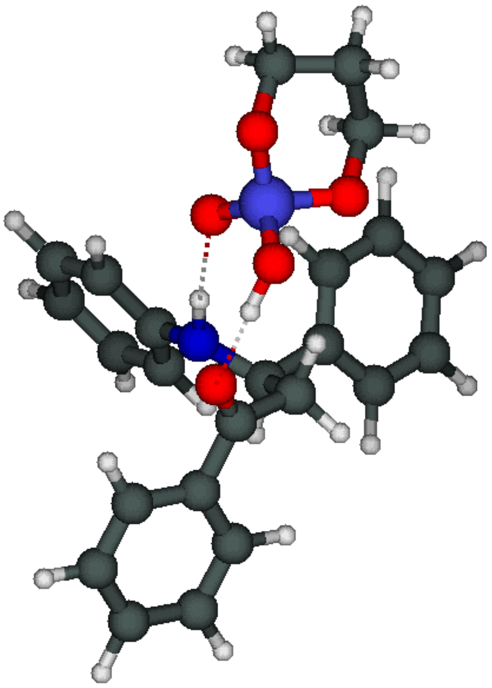

1	15	0	-0.008167	-0.063609	-0.230773
2	8	0	-0.041489	-1.029948	1.055082
3	6	0	1.176609	-1.102125	1.826673
4	6	0	1.563069	0.283890	2.314656
5	6	0	1.738264	1.253399	1.158275
6	8	0	0.529056	1.322199	0.368381
7	1	0	0.968180	-1.781007	2.661344
8	1	0	1.973403	-1.539958	1.203553
9	1	0	0.787395	0.661671	2.997005
10	1	0	2.508660	0.218833	2.870989
11	1	0	1.922855	2.273067	1.513791
12	1	0	2.572520	0.950944	0.506028
13	8	0	0.800209	-0.607957	-1.360837
14	8	0	-1.517883	0.233098	-0.540587
15	1	0	0.335418	-2.107408	-2.435785
16	7	0	0.172920	-3.001165	-2.904060

17	6	0	-0.714315	-3.899883	-2.198864
18	6	0	-0.066819	-4.648766	-1.041438
19	6	0	-0.516973	-5.925122	-0.691749
20	6	0	0.037278	-6.598098	0.397904
21	6	0	1.051522	-5.998986	1.146134
22	6	0	1.507702	-4.725811	0.799256
23	6	0	0.951021	-4.052643	-0.289260
24	1	0	-1.306833	-6.397257	-1.283035
25	1	0	-0.319542	-7.596141	0.659291
26	1	0	1.490594	-6.525811	1.995300
27	1	0	2.305732	-4.255172	1.377233
28	1	0	1.308160	-3.055005	-0.561191
29	1	0	-1.099675	-4.643651	-2.912681
30	6	0	1.274711	-3.471898	-3.599727
31	6	0	2.272157	-2.554619	-3.990075
32	6	0	3.374600	-2.975195	-4.723555
33	6	0	3.520854	-4.317606	-5.088893
34	6	0	2.542102	-5.229489	-4.698266
35	6	0	1.431114	-4.823430	-3.956833
36	1	0	2.158182	-1.505626	-3.707103
37	1	0	4.131667	-2.243742	-5.014295
38	1	0	4.387223	-4.644804	-5.665364
39	1	0	2.640866	-6.283761	-4.965848
40	1	0	0.689580	-5.564109	-3.656114
41	6	0	-1.911169	-3.079916	-1.678295
42	6	0	-2.463888	-2.174990	-2.751662
43	1	0	-2.692510	-3.763586	-1.316643
44	1	0	-1.593939	-2.460185	-0.827354
45	8	0	-2.425860	-0.956079	-2.621728
46	6	0	-3.035161	-2.760382	-3.993737
47	1	0	-1.866663	-0.234180	-1.369459
48	6	0	-3.139271	-1.945893	-5.131136
49	6	0	-3.670021	-2.458447	-6.309856
50	6	0	-4.114116	-3.782644	-6.356560
51	6	0	-4.023910	-4.594257	-5.225362
52	6	0	-3.480152	-4.089135	-4.045765
53	1	0	-2.788333	-0.914372	-5.079808
54	1	0	-3.738061	-1.826598	-7.196589
55	1	0	-4.533542	-4.183491	-7.281087
56	1	0	-4.378502	-5.625271	-5.260378
57	1	0	-3.422179	-4.730601	-3.165084

3.2. The specie involved in the *Mannich* reaction among **9a** and **8b** or **8e**.

8b+9a

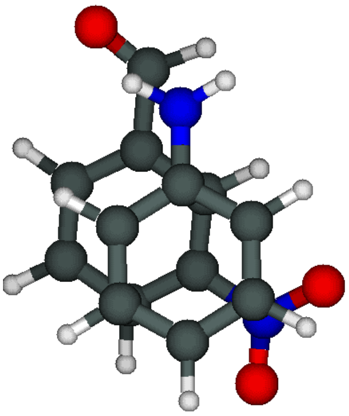

1	6	0	-0.938771	0.674376	-0.635258
2	6	0	-1.552821	0.457697	0.597657
3	6	0	-1.047186	-0.543615	1.417167
4	6	0	0.049179	-1.322981	1.058338
5	6	0	0.662243	-1.083928	-0.171361
6	6	0	0.166702	-0.095266	-1.018464
7	1	0	-2.419869	1.043794	0.904438
8	7	0	-1.711389	-0.802150	2.702492
9	1	0	0.410458	-2.098353	1.732514
10	1	0	1.525339	-1.680602	-0.467434
11	1	0	0.630686	0.091341	-1.988879
12	6	0	-1.503486	1.708552	-1.541612
13	8	0	-1.076938	1.933286	-2.649666
14	1	0	-2.348344	2.292835	-1.118768
15	7	0	-3.792073	-0.187091	-1.838702
16	6	0	-3.608456	-1.288371	-1.013345
17	6	0	-4.275201	-1.377051	0.221188
18	6	0	-4.005290	-2.427528	1.096057
19	6	0	-3.066941	-3.408603	0.766234
20	6	0	-2.409248	-3.328017	-0.464474
21	6	0	-2.672672	-2.283262	-1.346877
22	1	0	-4.999610	-0.605195	0.492114
23	1	0	-4.530658	-2.473187	2.052703
24	1	0	-2.854446	-4.226240	1.456576
25	1	0	-1.675112	-4.087908	-0.741868
26	1	0	-2.148480	-2.220904	-2.303729
27	1	0	-3.558047	-0.337144	-2.813917
28	1	0	-4.686839	0.279828	-1.740900
29	8	0	-1.321617	-1.737561	3.366445
30	8	0	-2.613683	-0.065003	3.033653

TS_{Add-N}

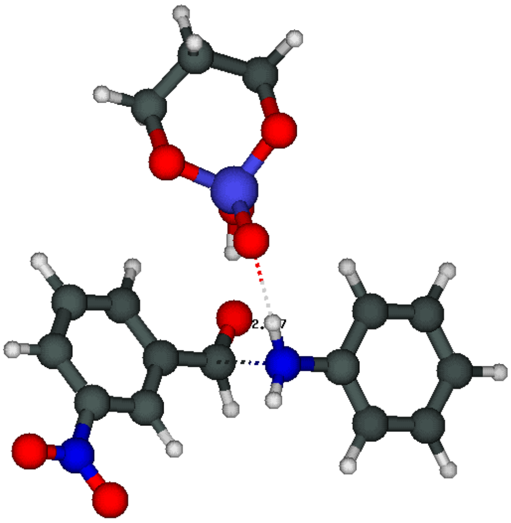

1	15	0	0.007012	0.039718	-0.004658
2	8	0	0.015210	-0.017920	1.596797
3	8	0	1.533847	0.038080	-0.401050
4	6	0	0.612107	1.092192	2.302660
5	6	0	-0.038741	2.398097	1.883204
6	1	0	0.456774	0.890733	3.368406
7	1	0	1.693859	1.100040	2.097892
8	6	0	0.061325	2.616246	0.383879
9	1	0	-1.097140	2.394058	2.183035
10	1	0	0.461557	3.228766	2.400517
11	8	0	-0.543949	1.514598	-0.325155
12	1	0	-0.485117	3.513564	0.072132
13	1	0	1.110570	2.711798	0.062979
14	1	0	1.792605	-0.443589	-1.280804
15	8	0	-0.842772	-1.014857	-0.617050
16	8	0	2.215190	-1.207819	-2.459294
17	6	0	1.455030	-1.559480	-3.375936
18	6	0	0.389110	-0.655019	-3.914607
19	6	0	-0.462066	-1.125537	-4.916017
20	6	0	-1.432078	-0.268066	-5.416529
21	6	0	-1.584093	1.042163	-4.967468
22	6	0	-0.716186	1.503780	-3.982783
23	6	0	0.268404	0.662862	-3.462194
24	1	0	-0.372625	-2.142843	-5.298659
25	7	0	-2.331596	-0.764523	-6.469558
26	1	0	-2.358950	1.680360	-5.389035
27	1	0	-0.803506	2.529127	-3.622607
28	1	0	0.964326	1.044670	-2.713867
29	1	0	1.781119	-2.355677	-4.067857
30	7	0	0.143747	-2.974547	-2.480086
31	6	0	0.962681	-3.926930	-1.858031
32	6	0	1.487575	-3.672078	-0.584485
33	6	0	2.368301	-4.581897	-0.001414
34	6	0	2.737841	-5.744659	-0.678721
35	6	0	2.218032	-5.992924	-1.951375
36	6	0	1.339709	-5.089341	-2.544130
37	1	0	1.191401	-2.764685	-0.055586
38	1	0	2.767159	-4.377358	0.993830
39	1	0	3.425508	-6.455228	-0.217676

40	1	0	2.499483	-6.899671	-2.490263
41	1	0	0.931487	-5.278354	-3.539618
42	1	0	-0.352494	-2.352829	-1.831523
43	1	0	-0.476002	-3.359448	-3.188983
44	8	0	-3.169741	-0.006279	-6.905314
45	8	0	-2.188171	-1.906705	-6.848218

X+Ib

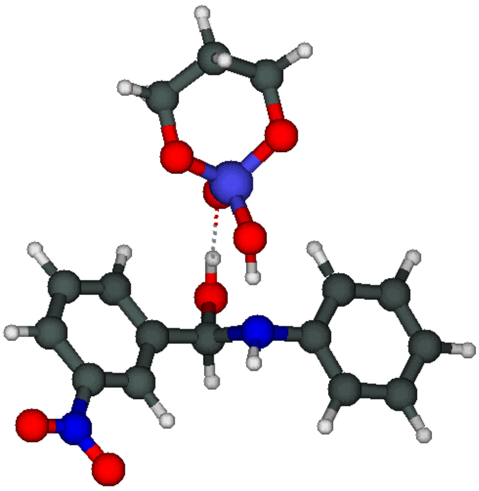

1	15	0	-0.018076	-0.001138	-0.007943
2	8	0	-0.009516	-0.007856	1.598123
3	8	0	1.362994	-0.010702	-0.597147
4	6	0	0.418525	1.211240	2.243692
5	6	0	-0.444578	2.377082	1.793424
6	1	0	0.322051	1.033018	3.320497
7	1	0	1.480614	1.385280	2.009669
8	6	0	-0.415702	2.544704	0.284160
9	1	0	-1.481105	2.214888	2.124043
10	1	0	-0.074253	3.298946	2.263527
11	8	0	-0.847402	1.327616	-0.362798
12	1	0	-1.109864	3.325430	-0.046270
13	1	0	0.595403	2.798969	-0.071269
14	1	0	1.841997	-1.022402	-1.863906
15	8	0	-0.938116	-1.184786	-0.422643
16	8	0	2.069506	-1.664387	-2.576709
17	6	0	0.936246	-2.252314	-3.084151
18	6	0	0.011126	-1.294007	-3.825420
19	6	0	-1.009980	-1.817780	-4.620008
20	6	0	-1.868764	-0.939033	-5.269217
21	6	0	-1.752079	0.444276	-5.165892
22	6	0	-0.723782	0.953488	-4.379986
23	6	0	0.153537	0.092208	-3.716394
24	1	0	-1.138858	-2.894878	-4.739125
25	7	0	-2.941506	-1.497928	-6.106799
26	1	0	-2.445980	1.096496	-5.693443
27	1	0	-0.600387	2.032709	-4.284795
28	1	0	0.960726	0.507693	-3.112039
29	1	0	1.263202	-3.041712	-3.780277
30	7	0	0.129767	-2.897906	-1.997446
31	6	0	0.828774	-3.913917	-1.258022

32	6	0	1.694243	-3.543225	-0.225252
33	6	0	2.365158	-4.528221	0.498155
34	6	0	2.168467	-5.878236	0.202293
35	6	0	1.297466	-6.243506	-0.824472
36	6	0	0.631327	-5.263060	-1.559215
37	1	0	1.836111	-2.486284	0.007373
38	1	0	3.041096	-4.236638	1.303668
39	1	0	2.691534	-6.645826	0.775041
40	1	0	1.135704	-7.297037	-1.058994
41	1	0	-0.049245	-5.538408	-2.368311
42	1	0	-0.474959	-1.893991	-1.083375
43	1	0	-0.686314	-3.326450	-2.438634
44	8	0	-3.698591	-0.724985	-6.652063
45	8	0	-3.015175	-2.703383	-6.209363

TS$_{De\text{-}H2O}$

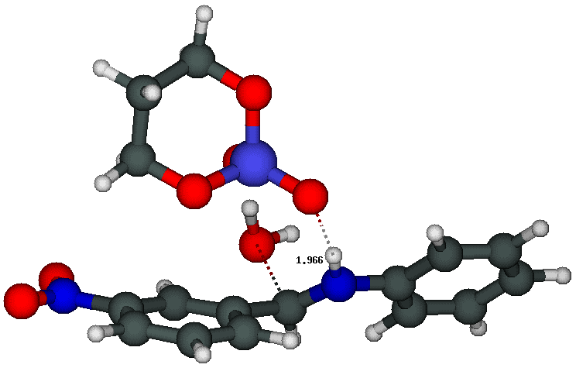

1	15	0	0.473861	-0.103153	0.088679
2	8	0	0.100366	1.011023	1.209759
3	8	0	1.964260	-0.408125	0.218270
4	6	0	0.801413	2.260288	1.099701
5	6	0	0.557515	2.902906	-0.258486
6	1	0	0.426223	2.898738	1.908921
7	1	0	1.876807	2.084368	1.262494
8	6	0	0.939545	1.971132	-1.400068
9	1	0	-0.507388	3.165281	-0.349757
10	1	0	1.146142	3.828921	-0.328522
11	8	0	0.228521	0.734598	-1.283759
12	1	0	0.663702	2.403020	-2.371523
13	1	0	2.024422	1.769892	-1.401509
14	1	0	2.693998	-1.511989	-0.296933
15	8	0	-0.513545	-1.236530	0.145787
16	8	0	3.180846	-2.337634	-0.724919
17	6	0	1.654252	-3.191606	-1.622321
18	6	0	1.288574	-2.104216	-2.577679
19	6	0	2.231845	-1.198258	-3.066541
20	6	0	1.802276	-0.221249	-3.953997
21	6	0	0.487764	-0.122118	-4.401006
22	6	0	-0.434329	-1.046221	-3.925248
23	6	0	-0.041653	-2.022844	-3.010843
24	1	0	3.270949	-1.232396	-2.749716
25	7	0	2.781934	0.771610	-4.421777
26	1	0	0.202601	0.661881	-5.100595
27	1	0	-1.471206	-1.000171	-4.258173

28	1	0	-0.775961	-2.738600	-2.638973
29	1	0	2.321986	-3.976333	-1.979108
30	7	0	0.777184	-3.492831	-0.690848
31	6	0	0.831570	-4.610142	0.172833
32	6	0	-0.165334	-4.705161	1.150071
33	6	0	-0.168103	-5.779969	2.034536
34	6	0	0.820101	-6.762072	1.952052
35	6	0	1.813705	-6.658289	0.978477
36	6	0	1.829476	-5.586003	0.087562
37	1	0	-0.932519	-3.930402	1.206749
38	1	0	-0.949190	-5.847941	2.793437
39	1	0	0.817649	-7.604133	2.645607
40	1	0	2.593253	-7.418632	0.908081
41	1	0	2.619180	-5.527939	-0.661937
42	1	0	0.125563	-2.725524	-0.405842
43	1	0	3.435657	-2.926073	-0.001162
44	8	0	2.396547	1.636729	-5.177877
45	8	0	3.923460	0.678063	-4.026533

X+IVb

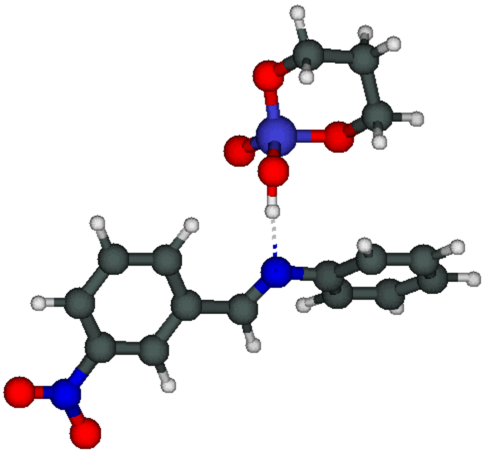

1	15	0	-0.038231	-0.212734	-0.003025
2	8	0	0.046401	-0.106501	1.594997
3	6	0	1.344811	0.103947	2.189919
4	6	0	2.015711	1.328129	1.592364
5	6	0	2.144435	1.211558	0.083367
6	8	0	0.846992	1.030747	-0.514860
7	1	0	1.167712	0.229438	3.264143
8	1	0	1.954250	-0.799862	2.035341
9	1	0	1.432265	2.226681	1.842702
10	1	0	3.017371	1.435646	2.031966
11	1	0	2.558970	2.125834	-0.357228
12	1	0	2.788928	0.362368	-0.194038
13	8	0	0.813538	-1.519672	-0.310387
14	8	0	-1.416986	-0.174771	-0.537435
15	1	0	0.739241	-1.814272	-1.284860
16	7	0	0.586474	-1.980046	-2.889199
17	6	0	-0.136461	-2.736402	-3.616586
18	6	0	-0.901767	-3.876276	-3.086293
19	6	0	-1.589579	-4.680800	-3.998528
20	6	0	-2.315305	-5.762570	-3.514768

21	6	0	-2.386639	-6.074839	-2.161434
22	6	0	-1.699012	-5.262948	-1.260171
23	6	0	-0.963382	-4.172937	-1.714662
24	1	0	-1.560375	-4.468493	-5.066984
25	7	0	-3.038032	-6.609654	-4.476678
26	1	0	-2.968371	-6.931782	-1.825257
27	1	0	-1.742153	-5.484517	-0.193862
28	1	0	-0.437350	-3.542650	-0.996984
29	1	0	-0.208588	-2.567346	-4.701592
30	6	0	1.242187	-0.856542	-3.445051
31	6	0	2.537513	-0.569793	-2.997303
32	6	0	3.205968	0.551183	-3.482570
33	6	0	2.577464	1.404074	-4.392916
34	6	0	1.278123	1.127271	-4.821419
35	6	0	0.605624	0.000443	-4.351010
36	1	0	3.012038	-1.239971	-2.277814
37	1	0	4.220730	0.764639	-3.141774
38	1	0	3.097520	2.289439	-4.762106
39	1	0	0.776937	1.800054	-5.519449
40	1	0	-0.422399	-0.201695	-4.657267
41	8	0	-3.678767	-7.541652	-4.043202
42	8	0	-2.955053	-6.331760	-5.652990

8e+9a

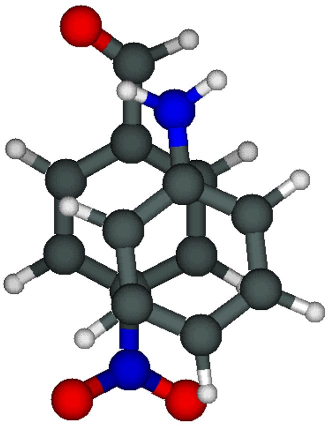

1	6	0	-0.044068	0.049847	0.001031
2	6	0	-0.043849	0.119281	1.396697
3	6	0	1.156397	0.084246	2.099308
4	6	0	2.334592	-0.025954	1.368922
5	6	0	2.367061	-0.075603	-0.023737
6	6	0	1.159713	-0.033557	-0.709029
7	1	0	-0.991595	0.178709	1.936847
8	7	0	3.603878	-0.136503	2.106125
9	1	0	1.183740	0.119218	3.187171
10	1	0	3.317698	-0.159722	-0.547592
11	1	0	1.139496	-0.081974	-1.798939
12	6	0	-1.349180	0.016958	-0.714991
13	8	0	-1.452583	-0.051650	-1.916406

14	1	0	-2.245806	0.077580	-0.061451
15	7	0	-1.365566	-2.815324	0.308273
16	6	0	-0.287908	-3.085273	1.141691
17	6	0	-0.447411	-3.091438	2.539085
18	6	0	0.653203	-3.261243	3.375207
19	6	0	1.935933	-3.423138	2.844233
20	6	0	2.096117	-3.429838	1.456061
21	6	0	1.000817	-3.265199	0.610597
22	1	0	-1.446216	-2.953266	2.960092
23	1	0	0.506171	-3.261538	4.457428
24	1	0	2.797009	-3.550750	3.502125
25	1	0	3.090239	-3.561643	1.021875
26	1	0	1.133576	-3.266688	-0.474014
27	1	0	-1.268730	-3.154588	-0.642384
28	1	0	-2.274718	-3.047788	0.692439
29	8	0	4.611058	-0.363948	1.471788
30	8	0	3.577114	-0.001294	3.309528

TS$_{Add-N}$

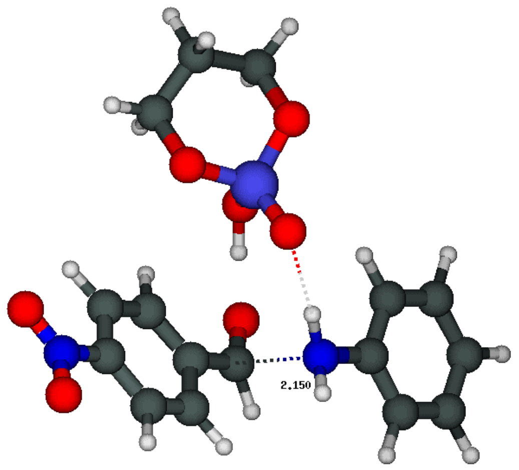

1	15	0	-0.010781	0.005486	-0.017263
2	8	0	-0.033215	0.004210	1.584606
3	8	0	1.526640	-0.015473	-0.375597
4	6	0	0.552601	1.138222	2.261776
5	6	0	-0.087032	2.429631	1.784062
6	1	0	0.376234	0.975100	3.330789
7	1	0	1.638070	1.136913	2.077715
8	6	0	0.041895	2.594745	0.280110
9	1	0	-1.151009	2.437992	2.063399
10	1	0	0.405191	3.277031	2.281601
11	8	0	-0.550804	1.468856	-0.400275
12	1	0	-0.497987	3.480486	-0.073465
13	1	0	1.097086	2.679004	-0.024248
14	1	0	1.801700	-0.484195	-1.251882
15	8	0	-0.850031	-1.067533	-0.611238
16	8	0	2.249906	-1.243767	-2.445043
17	6	0	1.493380	-1.569840	-3.371130
18	6	0	0.429180	-0.649981	-3.888924
19	6	0	-0.462018	-1.103924	-4.869629
20	6	0	-1.442566	-0.259864	-5.373396
21	6	0	-1.502688	1.042712	-4.881583

22	6	0	-0.619913	1.528779	-3.923790
23	6	0	0.357276	0.668928	-3.431314
24	1	0	-0.386486	-2.129069	-5.238608
25	1	0	-2.147742	-0.594964	-6.131960
26	7	0	-2.537503	1.946858	-5.407613
27	1	0	-0.694232	2.558544	-3.578491
28	1	0	1.079046	1.038009	-2.702231
29	1	0	1.808816	-2.362109	-4.072510
30	7	0	0.151261	-3.003257	-2.494432
31	6	0	0.970214	-3.955202	-1.874411
32	6	0	1.480275	-3.710592	-0.592484
33	6	0	2.362458	-4.619697	-0.010454
34	6	0	2.748831	-5.771817	-0.696472
35	6	0	2.243956	-6.010097	-1.977104
36	6	0	1.364276	-5.107337	-2.568898
37	1	0	1.170932	-2.811680	-0.056569
38	1	0	2.749350	-4.422877	0.991074
39	1	0	3.437796	-6.481672	-0.236277
40	1	0	2.538528	-6.908421	-2.523072
41	1	0	0.968393	-5.288593	-3.570804
42	1	0	-0.351803	-2.387545	-1.845928
43	1	0	-0.458103	-3.381964	-3.215100
44	8	0	-2.579762	3.077297	-4.973642
45	8	0	-3.295656	1.515089	-6.248503

X+Ie

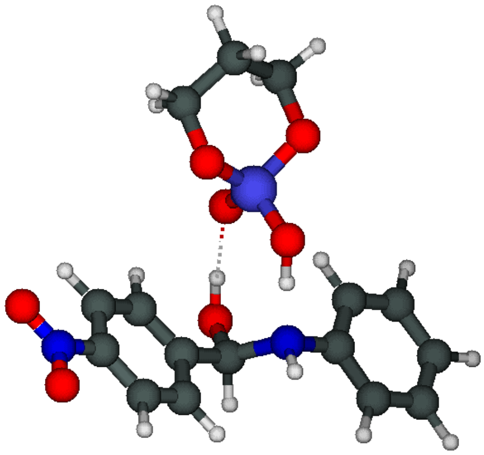

1	15	0	0.000808	0.003758	-0.003143
2	8	0	-0.000111	-0.006698	1.603058
3	8	0	1.385496	0.004694	-0.583649
4	6	0	0.416426	1.214039	2.253100
5	6	0	-0.452628	2.374754	1.800961
6	1	0	0.316128	1.033038	3.329097
7	1	0	1.478393	1.395845	2.024442
8	6	0	-0.417327	2.545863	0.292207
9	1	0	-1.489620	2.204518	2.126049
10	1	0	-0.091236	3.298230	2.274841
11	8	0	-0.836304	1.326807	-0.359564
12	1	0	-1.115826	3.321793	-0.040326
13	1	0	0.593579	2.808588	-0.057512
14	1	0	1.875601	-0.995989	-1.854412

15	8	0	-0.908734	-1.185824	-0.425225
16	8	0	2.109566	-1.633956	-2.568969
17	6	0	0.982184	-2.229905	-3.079485
18	6	0	0.043840	-1.279862	-3.813783
19	6	0	-0.989592	-1.813096	-4.595098
20	6	0	-1.884391	-0.978571	-5.253036
21	6	0	-1.721454	0.397690	-5.115760
22	6	0	-0.699985	0.958346	-4.357682
23	6	0	0.187922	0.105848	-3.705695
24	1	0	-1.092945	-2.896506	-4.696473
25	1	0	-2.690678	-1.379247	-5.864933
26	7	0	-2.661104	1.292217	-5.806575
27	1	0	-0.601821	2.040003	-4.282392
28	1	0	0.999243	0.529856	-3.114092
29	1	0	1.316725	-3.010908	-3.781845
30	7	0	0.181932	-2.890059	-1.996776
31	6	0	0.893465	-3.897862	-1.258130
32	6	0	1.749900	-3.517696	-0.221276
33	6	0	2.432209	-4.494913	0.501995
34	6	0	2.255824	-5.846809	0.201962
35	6	0	1.393916	-6.221695	-0.828982
36	6	0	0.716548	-5.248881	-1.563651
37	1	0	1.875777	-2.459401	0.014262
38	1	0	3.101092	-4.195810	1.310636
39	1	0	2.787671	-6.608407	0.774631
40	1	0	1.248138	-7.276806	-1.066883
41	1	0	0.043304	-5.532019	-2.376135
42	1	0	-0.437137	-1.891124	-1.081842
43	1	0	-0.627884	-3.328554	-2.439547
44	8	0	-2.503481	2.487836	-5.685853
45	8	0	-3.547997	0.789882	-6.462633

$TS_{De\text{-}H2O}$

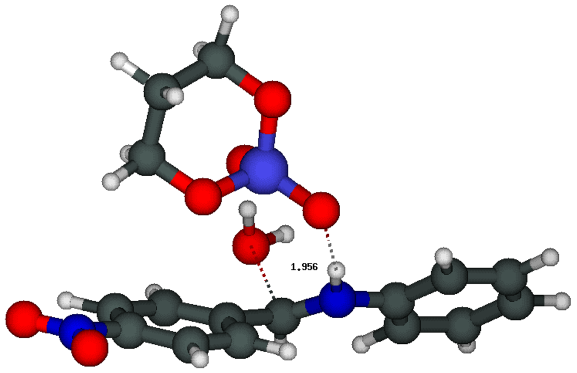

1	15	0	0.384986	-0.074859	0.116725
2	8	0	-0.099295	0.997946	1.235506
3	8	0	1.873272	-0.337799	0.335220
4	6	0	0.547732	2.279500	1.195319
5	6	0	0.353535	2.942138	-0.160724
6	1	0	0.096743	2.879966	1.994842
7	1	0	1.619095	2.149358	1.417700
8	6	0	0.855147	2.057756	-1.292890
9	1	0	-0.715986	3.152055	-0.313081
10	1	0	0.896526	3.898110	-0.175090

11	8	0	0.201807	0.785169	-1.251820
12	1	0	0.622895	2.498781	-2.271981
13	1	0	1.945796	1.909643	-1.225706
14	1	0	2.645521	-1.430103	-0.138712
15	8	0	-0.566945	-1.239183	0.088050
16	8	0	3.171997	-2.251722	-0.529774
17	6	0	1.753281	-3.118074	-1.560587
18	6	0	1.450203	-2.016162	-2.521006
19	6	0	2.451519	-1.151907	-2.984179
20	6	0	2.133388	-0.128219	-3.866612
21	6	0	0.812947	-0.002081	-4.288039
22	6	0	-0.193171	-0.864446	-3.875104
23	6	0	0.135338	-1.878267	-2.981483
24	1	0	3.476445	-1.266530	-2.636198
25	1	0	2.893462	0.565169	-4.222168
26	7	0	0.467331	1.103720	-5.195439
27	1	0	-1.213161	-0.738769	-4.233590
28	1	0	-0.640837	-2.565148	-2.644245
29	1	0	2.460208	-3.883679	-1.882241
30	7	0	0.810378	-3.455393	-0.707297
31	6	0	0.822626	-4.586334	0.139479
32	6	0	-0.240727	-4.716215	1.039953
33	6	0	-0.287062	-5.805722	1.904960
34	6	0	0.723208	-6.768340	1.879810
35	6	0	1.781873	-6.630917	0.982077
36	6	0	1.841778	-5.543485	0.111604
37	1	0	-1.024876	-3.956582	1.052795
38	1	0	-1.119529	-5.900128	2.604027
39	1	0	0.686916	-7.621583	2.558589
40	1	0	2.578621	-7.376120	0.956307
41	1	0	2.681703	-5.459856	-0.578266
42	1	0	0.129855	-2.702550	-0.452355
43	1	0	3.368797	-2.841500	0.211282
44	8	0	-0.687768	1.214361	-5.543633
45	8	0	1.354726	1.851296	-5.545056

X+IVe

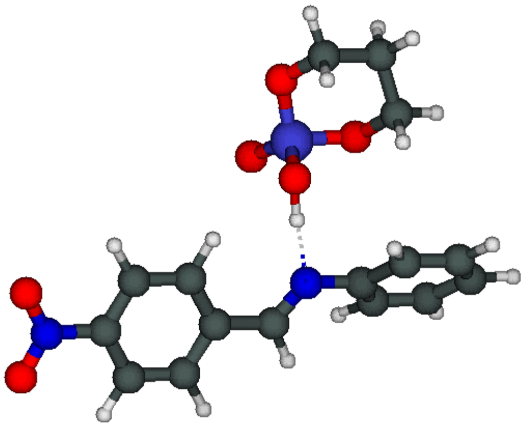

1	15	0	-0.037822	-0.097724	0.074763
2	8	0	0.045255	-0.117979	1.675745
3	6	0	1.351166	-0.037746	2.286513
4	6	0	2.103705	1.180619	1.781970

5	6	0	2.236651	1.164419	0.269179
6	8	0	0.934763	1.114327	-0.345593
7	1	0	1.175660	0.018980	3.366708
8	1	0	1.901458	-0.965497	2.065830
9	1	0	1.577417	2.094660	2.094961
10	1	0	3.106800	1.190571	2.231218
11	1	0	2.715134	2.077678	-0.103527
12	1	0	2.824590	0.295616	-0.066402
13	8	0	0.723655	-1.434309	-0.328944
14	8	0	-1.407412	0.075418	-0.456295
15	1	0	0.663506	-1.632474	-1.326978
16	7	0	0.571737	-1.655637	-2.951915
17	6	0	-0.200577	-2.262123	-3.764376
18	6	0	-1.144858	-3.314796	-3.352267
19	6	0	-1.861158	-3.980102	-4.356470
20	6	0	-2.766369	-4.984699	-4.031885
21	6	0	-2.938123	-5.300266	-2.689080
22	6	0	-2.246132	-4.655666	-1.665631
23	6	0	-1.344100	-3.657054	-2.005216
24	1	0	-1.705633	-3.707030	-5.401704
25	1	0	-3.329373	-5.512713	-4.799435
26	7	0	-3.895872	-6.360046	-2.332316
27	1	0	-2.416777	-4.933330	-0.627093
28	1	0	-0.800452	-3.138490	-1.215885
29	1	0	-0.180749	-2.024295	-4.838325
30	6	0	1.408688	-0.608227	-3.402877
31	6	0	2.700232	-0.528427	-2.867589
32	6	0	3.547357	0.507118	-3.253967
33	6	0	3.102526	1.480467	-4.151501
34	6	0	1.806971	1.410584	-4.666697
35	6	0	0.956255	0.370431	-4.296513
36	1	0	3.032107	-1.291498	-2.160889
37	1	0	4.558408	0.558580	-2.845976
38	1	0	3.762924	2.298770	-4.442761
39	1	0	1.449170	2.178592	-5.354549
40	1	0	-0.068124	0.329723	-4.671305
41	8	0	-4.044816	-6.619242	-1.158456
42	8	0	-4.485332	-6.919061	-3.231031

3.3. The specie involved in the aldol condensation between **10a** and **12a**.

10a+12a

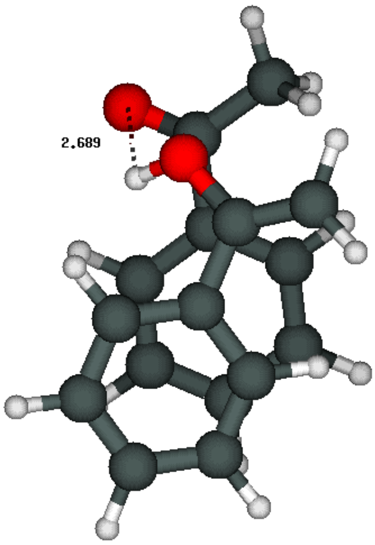

1	6	0	-0.145179	-0.083580	0.289608
2	6	0	-0.578896	0.212809	1.588812
3	6	0	0.317258	0.154718	2.655667
4	6	0	1.645260	-0.213238	2.434103
5	6	0	2.080566	-0.526299	1.143530
6	6	0	1.188995	-0.457653	0.076073
7	1	0	-1.614849	0.500738	1.773143
8	1	0	-0.023418	0.394519	3.664319
9	1	0	2.343905	-0.262620	3.271781
10	1	0	3.117592	-0.820252	0.971188
11	1	0	1.515853	-0.690082	-0.939335
12	6	0	-1.073972	-0.012686	-0.888970
13	8	0	-0.654189	-0.224793	-2.010504
14	6	0	-2.519222	0.318337	-0.638891
15	6	0	-1.538176	-3.152856	0.650765
16	6	0	-0.190931	-3.521640	1.160782
17	6	0	0.128025	-3.367613	2.518441
18	6	0	1.390326	-3.715130	2.992415
19	6	0	2.360154	-4.210236	2.117851
20	6	0	2.055007	-4.363038	0.765817
21	6	0	0.788760	-4.023113	0.289934
22	1	0	-0.611774	-2.951548	3.204517
23	1	0	1.623788	-3.583039	4.050591
24	1	0	3.351226	-4.475343	2.490274
25	1	0	2.803116	-4.754946	0.074625
26	1	0	0.565706	-4.174130	-0.769003
27	1	0	-2.608192	1.325891	-0.205286
28	1	0	-3.074980	0.271041	-1.581911
29	1	0	-2.948777	-0.389936	0.085157
30	6	0	-2.650850	-3.121925	1.397471
31	8	0	-1.630738	-2.865341	-0.675938
32	1	0	-3.601802	-2.832329	0.948129
33	1	0	-2.631292	-3.424296	2.442737
34	1	0	-0.767254	-2.635868	-1.044806

TS$_{AldC}$

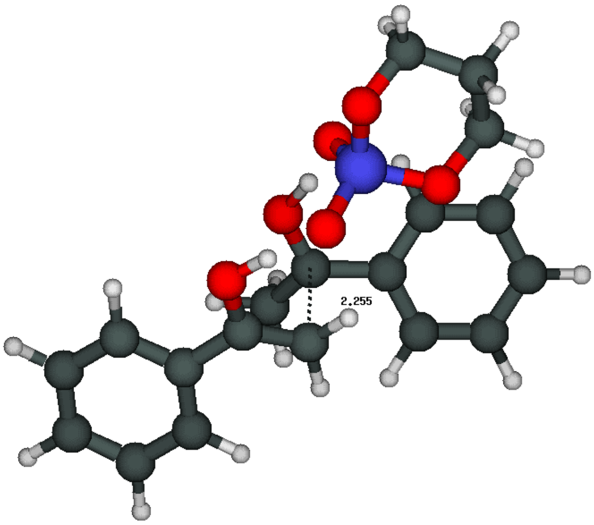

1	15	0	-0.064574	0.048378	0.015757
2	8	0	-0.011240	0.101747	1.631731
3	6	0	1.295770	0.041709	2.226555
4	6	0	2.158348	1.185891	1.718822
5	6	0	2.275286	1.163863	0.203660
6	8	0	0.971575	1.211519	-0.405146
7	1	0	1.145312	0.104031	3.311119
8	1	0	1.757044	-0.931220	1.990171
9	1	0	1.720796	2.143276	2.038981
10	1	0	3.163085	1.103316	2.157228
11	1	0	2.818900	2.040951	-0.166588
12	1	0	2.797535	0.256457	-0.139189
13	8	0	0.499913	-1.290931	-0.465144
14	8	0	-1.455533	0.417446	-0.433756
15	1	0	-0.274396	-2.364324	-0.591206
16	8	0	-0.889035	-3.237660	-0.834769
17	6	0	-1.725839	-3.712287	0.012205
18	6	0	-1.434030	-3.623148	1.480574
19	6	0	-2.389743	-4.015456	2.432443
20	6	0	-2.084059	-3.994503	3.788443
21	6	0	-0.815187	-3.594099	4.216569
22	6	0	0.144742	-3.222660	3.277883
23	6	0	-0.160152	-3.234784	1.915705
24	1	0	-3.386276	-4.326755	2.115557
25	1	0	-2.839442	-4.294072	4.516460
26	1	0	-0.575504	-3.581195	5.281195
27	1	0	1.143540	-2.925361	3.602348
28	1	0	0.605861	-2.955229	1.193155
29	6	0	-2.487029	-4.912927	-0.467279
30	6	0	-3.383643	-2.185324	0.092635
31	6	0	-3.493021	-1.995057	-1.267680
32	1	0	-4.119402	-2.790891	0.615905
33	1	0	-2.822122	-1.453845	0.674871
34	8	0	-2.731054	-1.154176	-1.915430
35	6	0	-4.375802	-2.813667	-2.132364
36	1	0	-2.191633	-0.533518	-1.299004
37	6	0	-4.112626	-2.884172	-3.508530
38	6	0	-4.901896	-3.680893	-4.335203
39	6	0	-5.967181	-4.405825	-3.799752

40	6	0	-6.243660	-4.329222	-2.432914
41	6	0	-5.453701	-3.539520	-1.602091
42	1	0	-3.278038	-2.317844	-3.921663
43	1	0	-4.683718	-3.736081	-5.402923
44	1	0	-6.586835	-5.027667	-4.448336
45	1	0	-7.083500	-4.884236	-2.011939
46	1	0	-5.689382	-3.480059	-0.538783
47	1	0	-1.828208	-5.782734	-0.303598
48	1	0	-3.416618	-5.081053	0.087893
49	1	0	-2.695322	-4.832721	-1.540752

3.4. The specie involved in the enolization of **10b** to **12b**.

10b

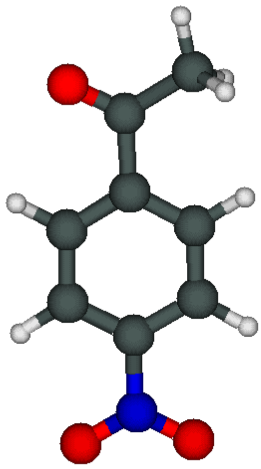

1	6	0	0.002685	-0.001069	0.005019
2	6	0	-0.001926	-0.001990	1.404981
3	6	0	1.197463	-0.001764	2.111418
4	6	0	2.385554	-0.000589	1.389787
5	6	0	2.424960	0.000317	-0.002185
6	6	0	1.218668	0.000045	-0.690573
7	1	0	-0.939955	-0.002880	1.959967
8	1	0	1.214181	-0.002460	3.199860
9	7	0	3.658757	-0.000259	2.130885
10	1	0	3.379348	0.001205	-0.525750
11	1	0	1.207438	0.000721	-1.780794
12	6	0	-1.280716	-0.001256	-0.791413
13	8	0	-1.234182	-0.000892	-2.002548
14	6	0	-2.583776	-0.001738	-0.044176
15	1	0	-2.648953	-0.888577	0.603608
16	1	0	-2.648923	0.883923	0.605198
17	1	0	-3.415251	-0.001136	-0.757235
18	8	0	4.687688	0.000599	1.491812
19	8	0	3.611619	-0.001130	3.341290

TS$_{Enol}$

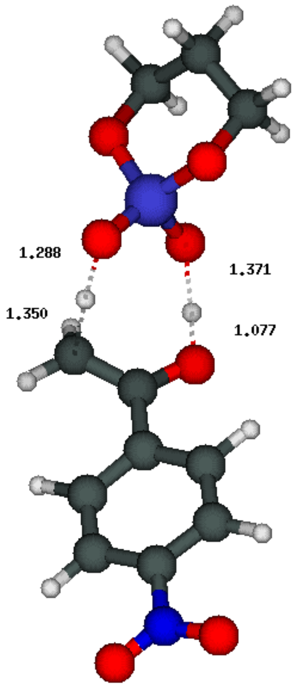

1	15	0	0.001131	-0.001218	0.005889
2	8	0	-0.004881	0.005261	1.612847
3	8	0	1.426515	-0.003904	-0.539664
4	6	0	0.491860	1.199429	2.255073
5	6	0	-0.289191	2.415175	1.787358
6	1	0	0.372312	1.036061	3.332034
7	1	0	1.565247	1.303808	2.031463
8	6	0	-0.228195	2.573146	0.278042
9	1	0	-1.338411	2.320701	2.104170
10	1	0	0.132295	3.314189	2.258822
11	8	0	-0.723303	1.382351	-0.371155
12	1	0	-0.868154	3.393857	-0.065031
13	1	0	0.801768	2.762849	-0.063108
14	1	0	1.937552	-1.212864	-0.935779
15	8	0	-0.877437	-1.164460	-0.459414
16	8	0	2.214913	-2.232161	-1.146545
17	6	0	1.357978	-2.850575	-1.877113
18	6	0	0.328627	-2.180301	-2.556270
19	1	0	-0.373415	-1.773823	-1.476621
20	1	0	0.566923	-1.166979	-2.896979
21	1	0	-0.313351	-2.763974	-3.215489
22	6	0	1.469492	-4.335016	-1.846262
23	6	0	0.933083	-5.118631	-2.876638
24	6	0	1.052972	-6.503136	-2.837092
25	6	0	1.708759	-7.076439	-1.753215
26	6	0	2.253712	-6.327187	-0.715450
27	6	0	2.134580	-4.944018	-0.772360
28	1	0	0.437899	-4.653115	-3.728190
29	1	0	0.650032	-7.128035	-3.632002
30	7	0	1.836170	-8.544775	-1.704475
31	1	0	2.757889	-6.818847	0.114514
32	1	0	2.550807	-4.328907	0.024789
33	8	0	2.405830	-9.033090	-0.754136
34	8	0	1.364343	-9.185258	-2.617385

12b

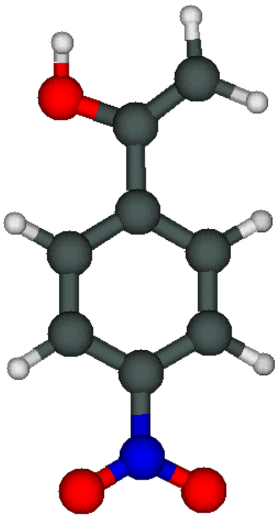

1	6	0	0.005329	-0.005042	0.005245
2	6	0	0.001742	-0.013085	1.410493
3	6	0	1.191818	-0.010753	2.122673
4	6	0	2.391947	0.005658	1.414588
5	6	0	2.434007	0.022096	0.025478
6	6	0	1.232364	0.019551	-0.674956
7	1	0	-0.938324	-0.009996	1.961133
8	1	0	1.196478	-0.014104	3.211264
9	7	0	3.654221	0.012135	2.162700
10	1	0	3.389874	0.036980	-0.495409
11	1	0	1.247025	0.032672	-1.763430
12	6	0	-1.261958	-0.017062	-0.767817
13	8	0	-1.056281	0.231041	-2.088189
14	6	0	-2.473499	-0.255086	-0.249594
15	1	0	-3.358680	-0.247093	-0.889325
16	1	0	-2.608560	-0.473674	0.807403
17	1	0	-1.895601	0.194694	-2.564385
18	8	0	4.690800	0.034911	1.533943
19	8	0	3.599267	-0.005842	3.374020

3.5. The specie involved in the addition of the enol **12e** to **IVa** or to **10e**.

12e$_{I}$

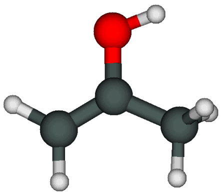

1	6	0	-0.176602	-0.124059	-0.107885
2	8	0	-0.233594	-0.458314	1.213455
3	6	0	1.198042	-0.088687	-0.698571
4	1	0	1.682127	-1.073389	-0.597589
5	1	0	1.165062	0.181306	-1.760401
6	1	0	1.823373	0.645338	-0.165245
7	6	0	-1.308156	0.141511	-0.767616
8	1	0	-2.272979	0.093117	-0.260167
9	1	0	-1.274259	0.409303	-1.822557
10	1	0	0.652722	-0.621795	1.559242

TS$_{AddI}$

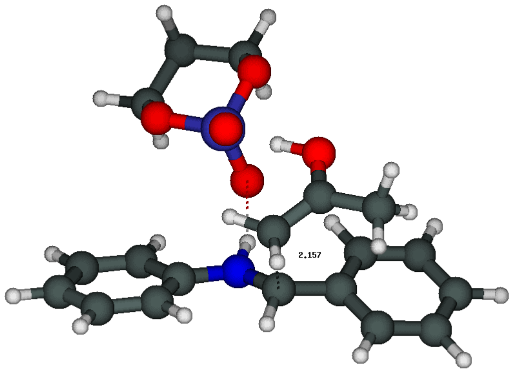

1	15	0	-0.017441	-0.004770	0.023980
2	8	0	0.024752	0.019087	1.650241
3	6	0	1.333594	0.036368	2.240098
4	6	0	2.115604	1.251813	1.765877
5	6	0	2.213265	1.297113	0.248488
6	8	0	0.903863	1.284327	-0.337781
7	1	0	1.185333	0.060777	3.326674
8	1	0	1.860654	-0.896131	1.980186
9	1	0	1.618716	2.166041	2.124278
10	1	0	3.128021	1.220704	2.193832
11	1	0	2.697424	2.221472	-0.089904
12	1	0	2.791433	0.439534	-0.133702
13	8	0	0.625485	-1.277856	-0.483412
14	8	0	-1.431443	0.321457	-0.419890
15	1	0	-0.110112	-1.762455	-2.030508
16	7	0	-0.610962	-1.717390	-2.937391
17	6	0	-1.645624	-2.518969	-3.172653
18	6	0	-1.797211	-3.780211	-2.424604
19	6	0	-2.549255	-4.806124	-3.014451

20	6	0	-2.716508	-6.021188	-2.355605
21	6	0	-2.140087	-6.216992	-1.098706
22	6	0	-1.394438	-5.196127	-0.506244
23	6	0	-1.218401	-3.978451	-1.162321
24	1	0	-3.002421	-4.644199	-3.995587
25	1	0	-3.296934	-6.817829	-2.823769
26	1	0	-2.270858	-7.168504	-0.580182
27	1	0	-0.942901	-5.347495	0.475504
28	1	0	-0.632863	-3.187220	-0.687874
29	1	0	-2.024994	-2.533243	-4.194254
30	6	0	-0.253974	-0.623252	-3.745459
31	6	0	0.932362	0.057079	-3.433559
32	6	0	1.331677	1.145508	-4.202214
33	6	0	0.558422	1.569439	-5.285447
34	6	0	-0.619881	0.890115	-5.592755
35	6	0	-1.034749	-0.201480	-4.829920
36	1	0	1.530396	-0.279597	-2.585890
37	1	0	2.257036	1.666851	-3.950217
38	1	0	0.873024	2.424083	-5.886189
39	1	0	-1.234268	1.212506	-6.435216
40	1	0	-1.964946	-0.708635	-5.086315
41	6	0	-3.402228	-1.294967	-2.676017
42	6	0	-3.695020	-1.852637	-1.445401
43	1	0	-2.836630	-0.362224	-2.714953
44	1	0	-4.063915	-1.525924	-3.511048
45	8	0	-3.082834	-1.519396	-0.347657
46	6	0	-4.678963	-2.960513	-1.274690
47	1	0	-2.396935	-0.732819	-0.429956
48	1	0	-4.178959	-3.817890	-0.796409
49	1	0	-5.476344	-2.624718	-0.593692
50	1	0	-5.120106	-3.268893	-2.228675

10e+12e$_l$

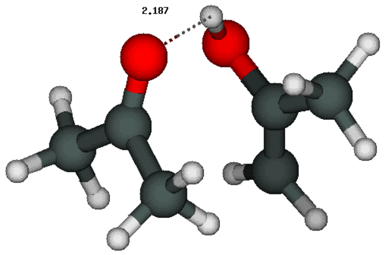

1	6	0	-0.270308	-0.247061	-0.177056
2	8	0	-0.602403	-0.669865	0.915529
3	6	0	1.173061	-0.170724	-0.590133
4	1	0	1.447377	0.887137	-0.727699
5	1	0	1.820980	-0.626922	0.166979
6	1	0	1.309641	-0.669646	-1.561302
7	6	0	-1.289837	0.203864	-1.186965
8	6	0	-0.471860	-3.354587	-0.951836
9	6	0	0.025517	-3.478212	-2.188154
10	1	0	1.104193	-3.499615	-2.353683
11	1	0	-0.645012	-3.584922	-3.040155
12	8	0	0.380202	-3.297724	0.111690

13	1	0	-0.015954	-2.775467	0.823920
14	6	0	-1.931019	-3.327609	-0.612218
15	1	0	-1.318525	-0.543333	-1.997533
16	1	0	-2.283096	0.287939	-0.730267
17	1	0	-0.990994	1.160087	-1.639976
18	1	0	-2.183844	-4.197191	0.014869
19	1	0	-2.178816	-2.423424	-0.033801
20	1	0	-2.548121	-3.350345	-1.518513

TS_{AldC}

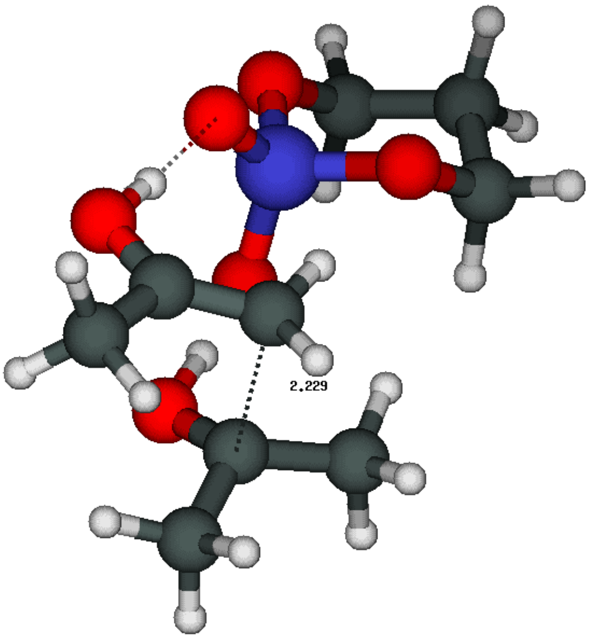

1	15	0	-0.009244	0.021902	-0.003043
2	8	0	-0.006117	0.008424	1.619579
3	6	0	1.284729	-0.018871	2.253905
4	6	0	2.113334	1.177114	1.814184
5	6	0	2.274015	1.222148	0.303564
6	8	0	0.986006	1.246115	-0.341002
7	1	0	1.098499	0.000846	3.334345
8	1	0	1.791838	-0.964366	2.001214
9	1	0	1.627791	2.102809	2.157868
10	1	0	3.107925	1.118012	2.278887
11	1	0	2.792505	2.134219	-0.014273
12	1	0	2.840209	0.350252	-0.060675
13	8	0	0.633889	-1.276330	-0.500131
14	8	0	-1.399735	0.350217	-0.476784
15	1	0	-0.161539	-2.320596	-0.753605
16	8	0	-0.834878	-3.145553	-0.982583
17	6	0	-1.370899	-3.709158	0.033027
18	6	0	-0.695662	-3.591167	1.370629
19	6	0	-2.131331	-4.966239	-0.249115
20	6	0	-3.060512	-2.322757	0.649220
21	6	0	-3.565458	-2.152567	-0.614365
22	1	0	-3.584343	-2.979402	1.342961
23	1	0	-2.386771	-1.570096	1.061572

24	8	0	-3.079595	-1.280837	-1.463935
25	6	0	-4.654320	-2.998506	-1.185408
26	1	0	-2.385584	-0.660512	-1.056219
27	1	0	-1.376149	-5.767408	-0.329643
28	1	0	-2.818016	-5.222409	0.565167
29	1	0	-2.662733	-4.904976	-1.205986
30	1	0	-5.509675	-2.353883	-1.439504
31	1	0	-4.310033	-3.464540	-2.121351
32	1	0	-4.981521	-3.771292	-0.481343
33	1	0	-0.315812	-2.579104	1.555025
34	1	0	-1.359845	-3.903834	2.183742
35	1	0	0.163871	-4.283057	1.335437

----

4. NMR spectra of precursors 2, 4 and catalyst 7.

(S) (-) 4-chlorobutane-1,3-diol (2)

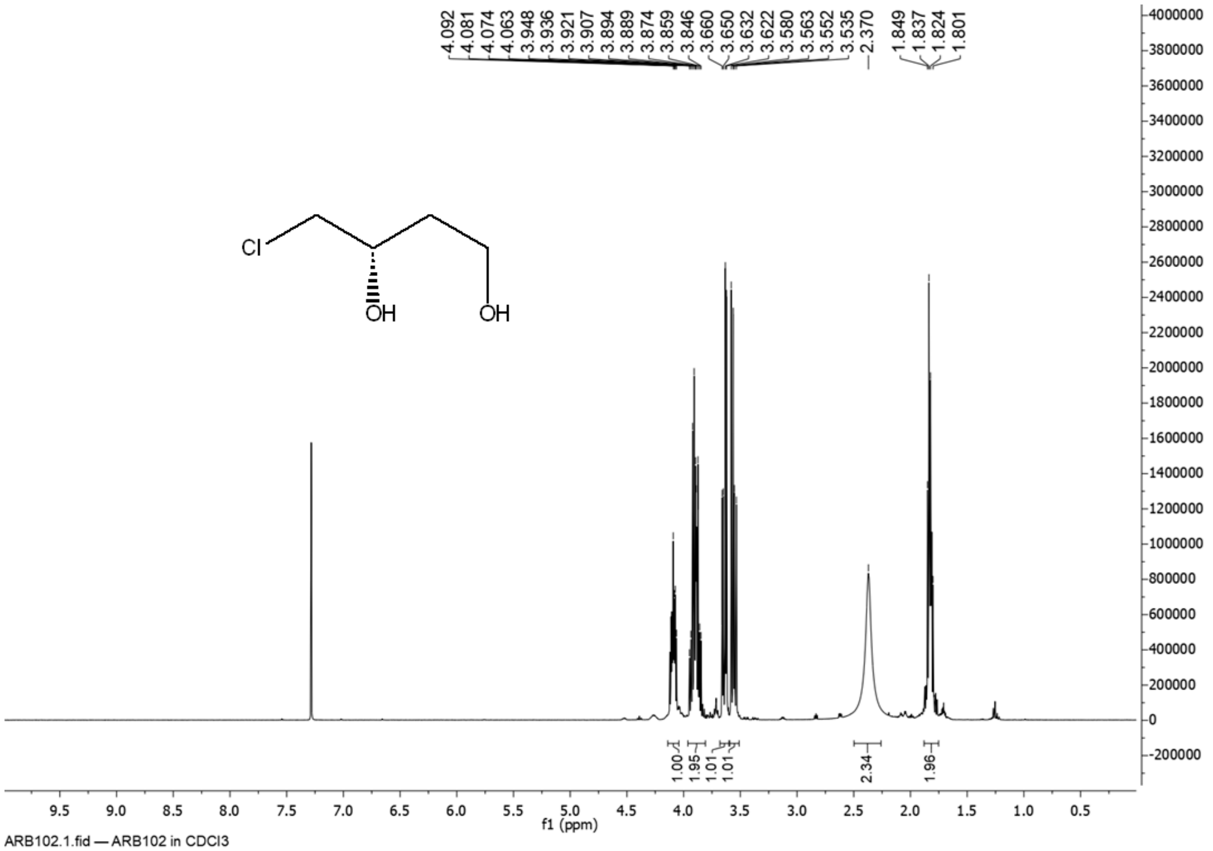

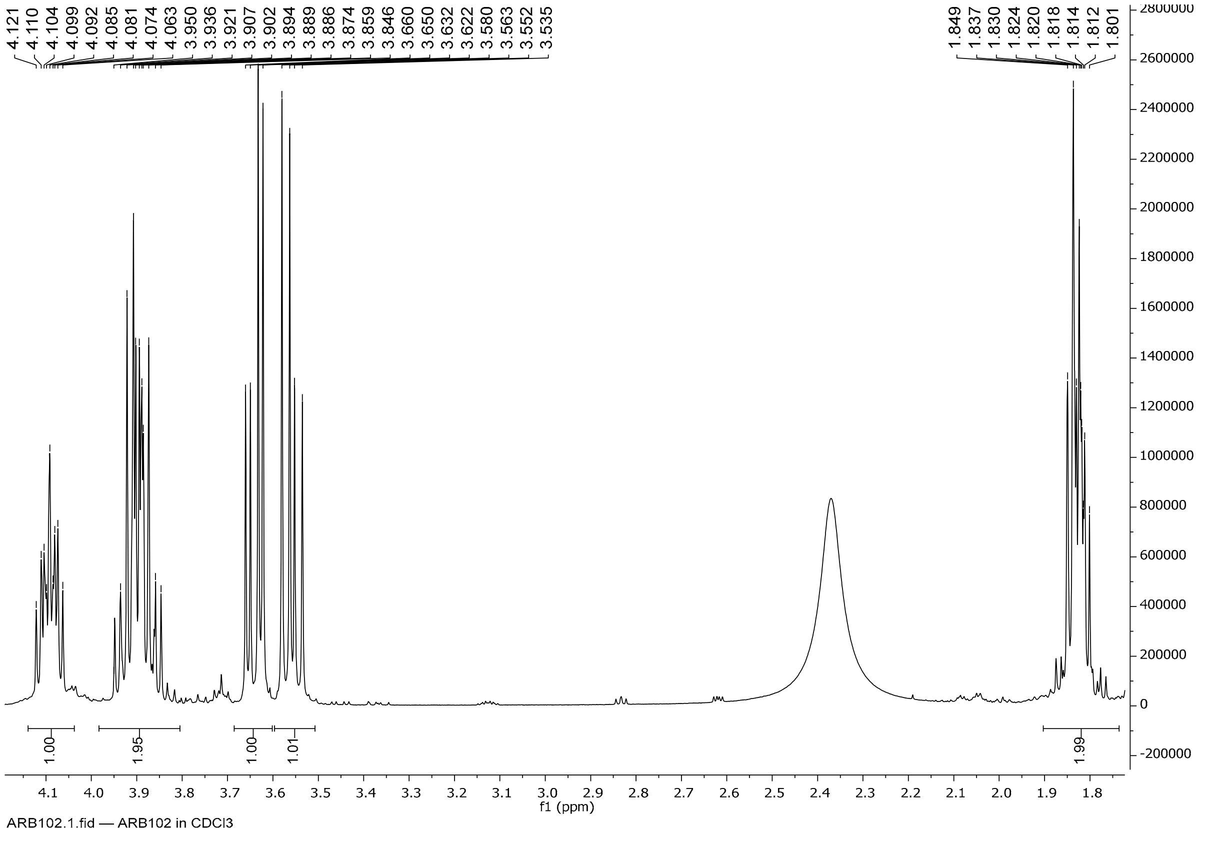

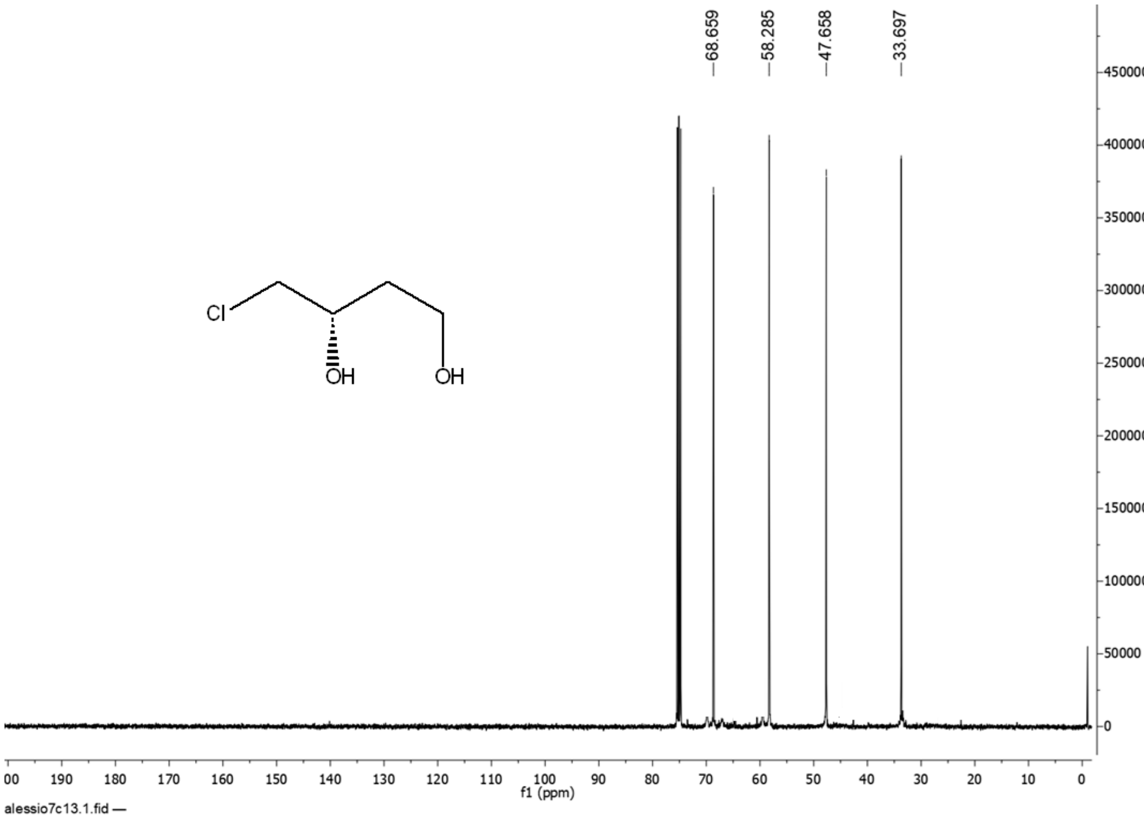

Cl
OH
OH
68.659
58.285
47.658
33.697
450000
400000
350000
300000
250000
200000
150000
100000
50000
0
00
190
180
170
160
150
140
130
120
110
100
90
80
70
60
50
40
30
20
10
0
f1 (ppm)
alessio7c13.1.fid

(-) 4-(Naphthalen-2-yloxy)butane-1,3-diol (4)

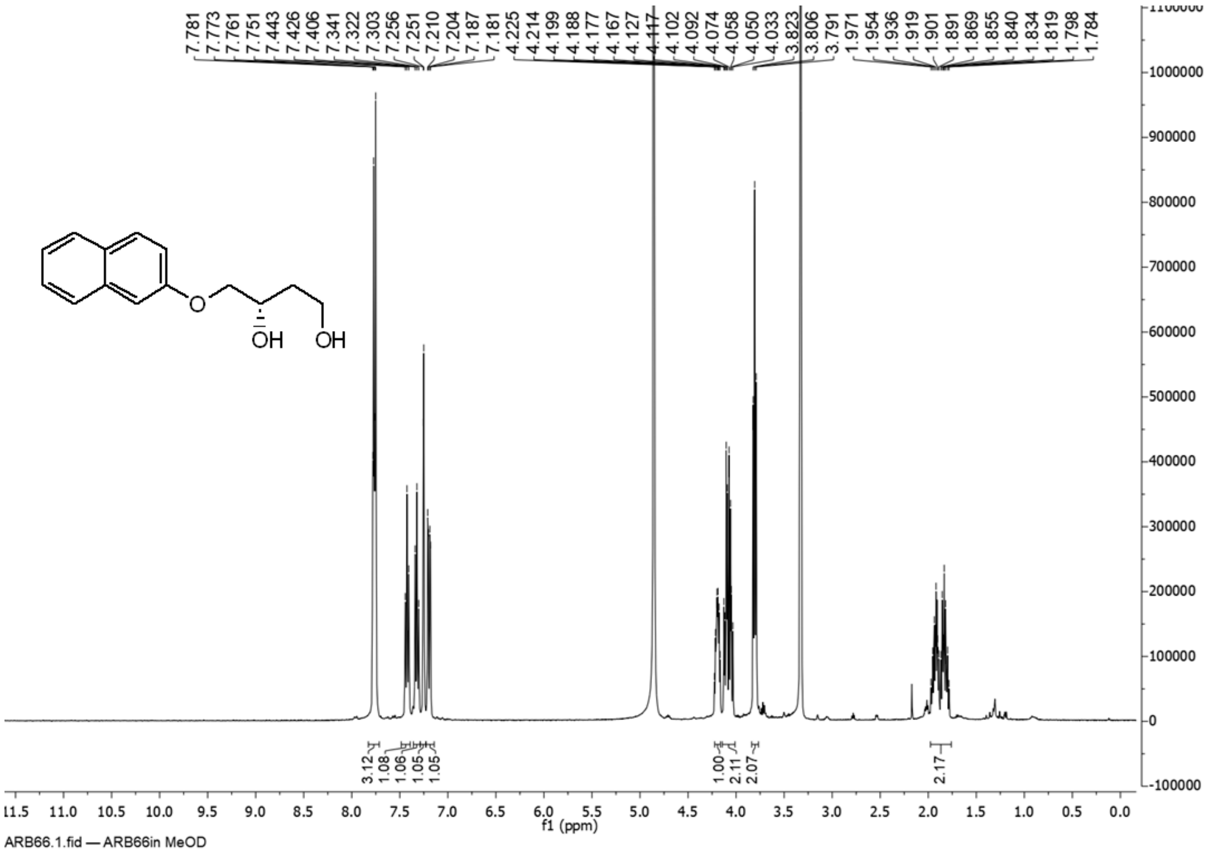

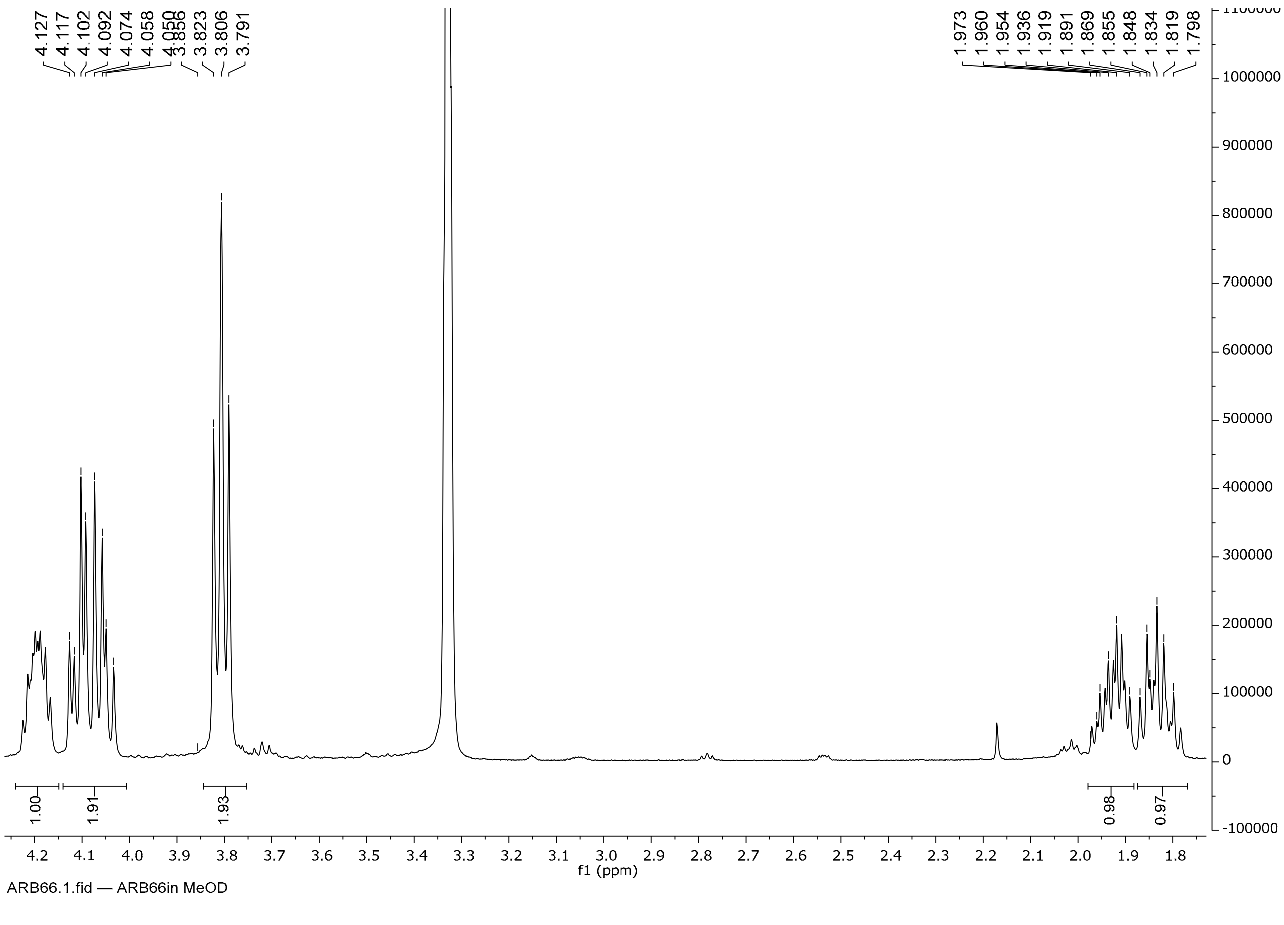

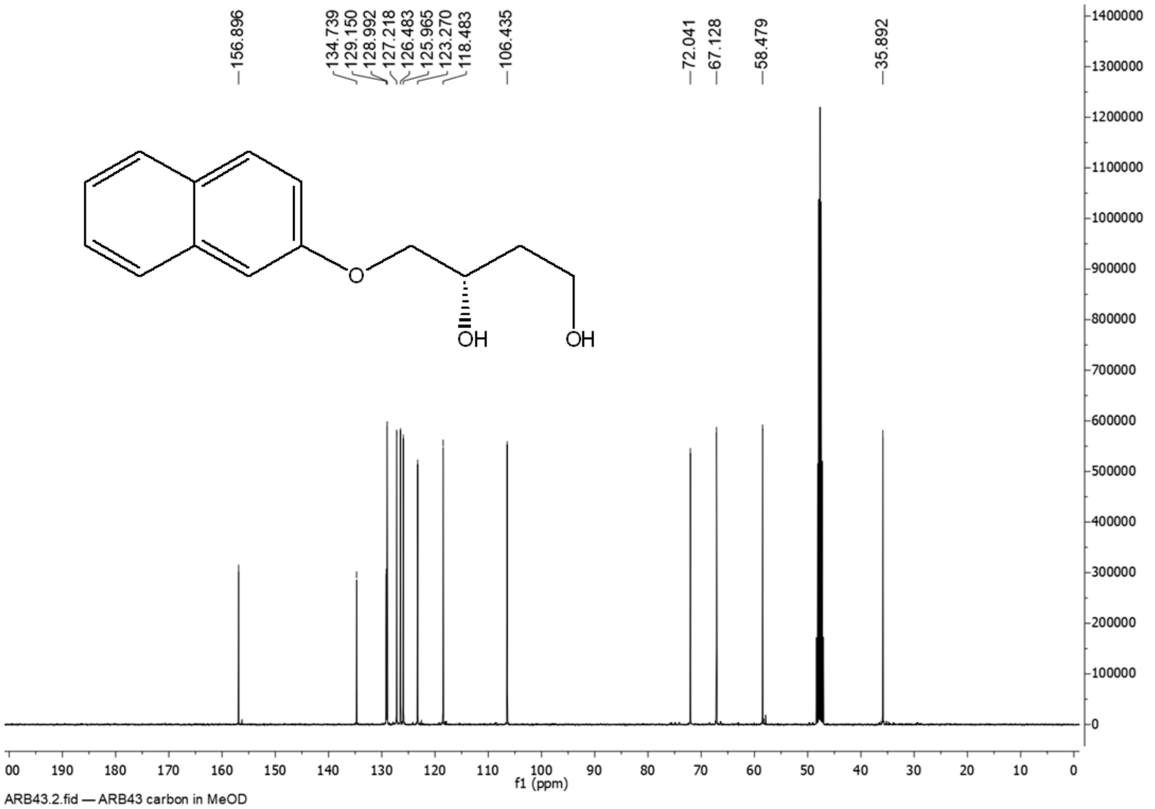
156.896
134.739
129.150
128.992
127.218
126.483
125.965
123.270
118.483
106.435
72.041
67.128
58.479
35.892
OH
OH
O
f1 (ppm)
ARB43.2.fid — ARB43 carbon in MeOD

(+) 2-Hydroxy-4-((naphthalen-2-yloxy)methyl)-1,3,2-dioxaphosphinane 2-oxide (7)

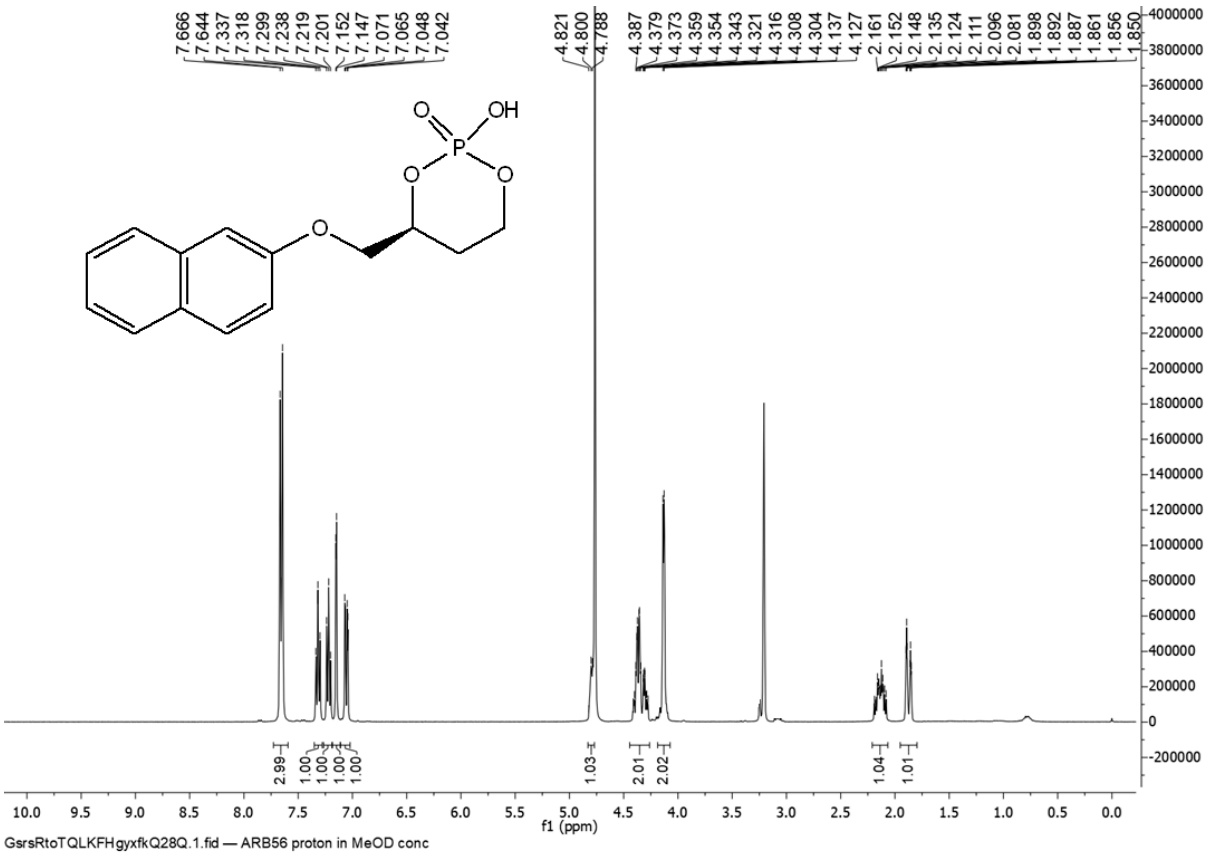

GsrsRtoTQLKFHgyxfkQ28Q.1.fid — ARB56 proton in MeOD conc

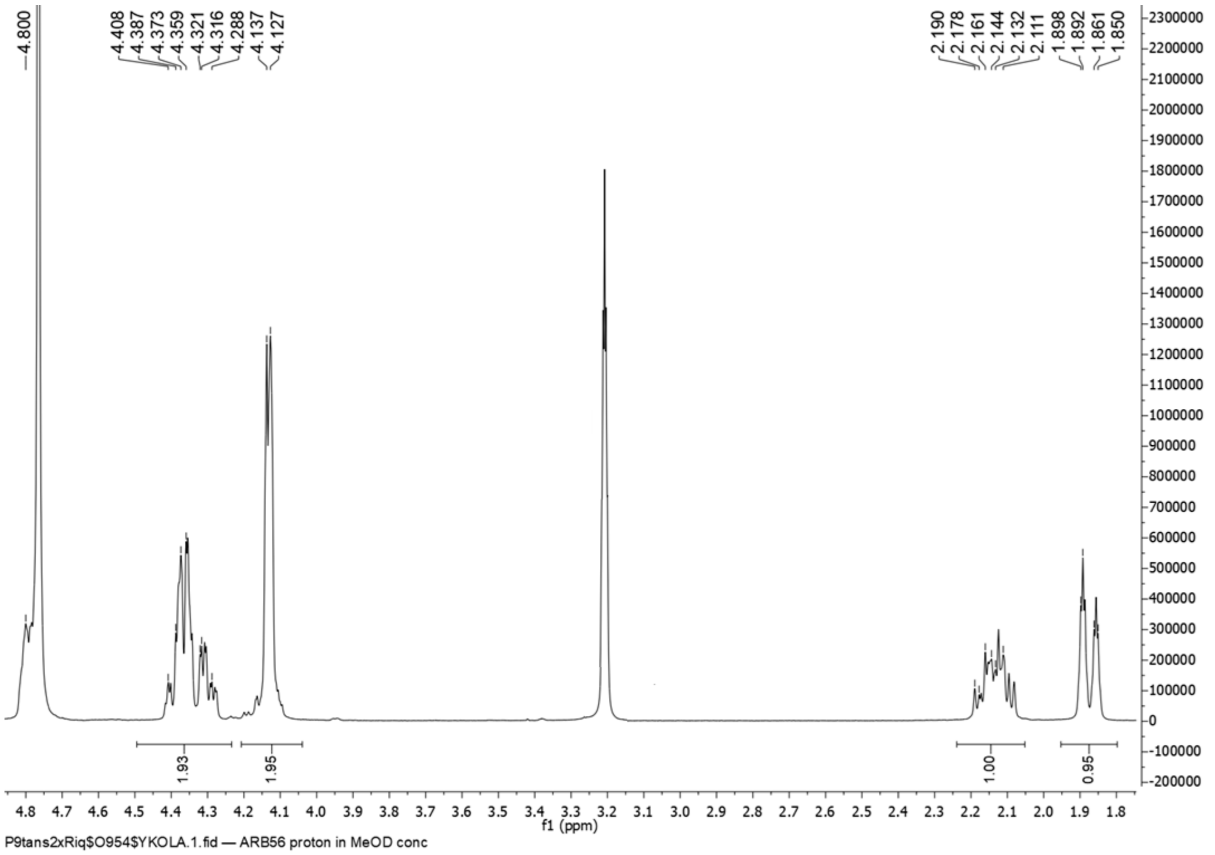

P9tans2xRiq$O954$YKOLA.1.fid — ARB56 proton in MeOD conc

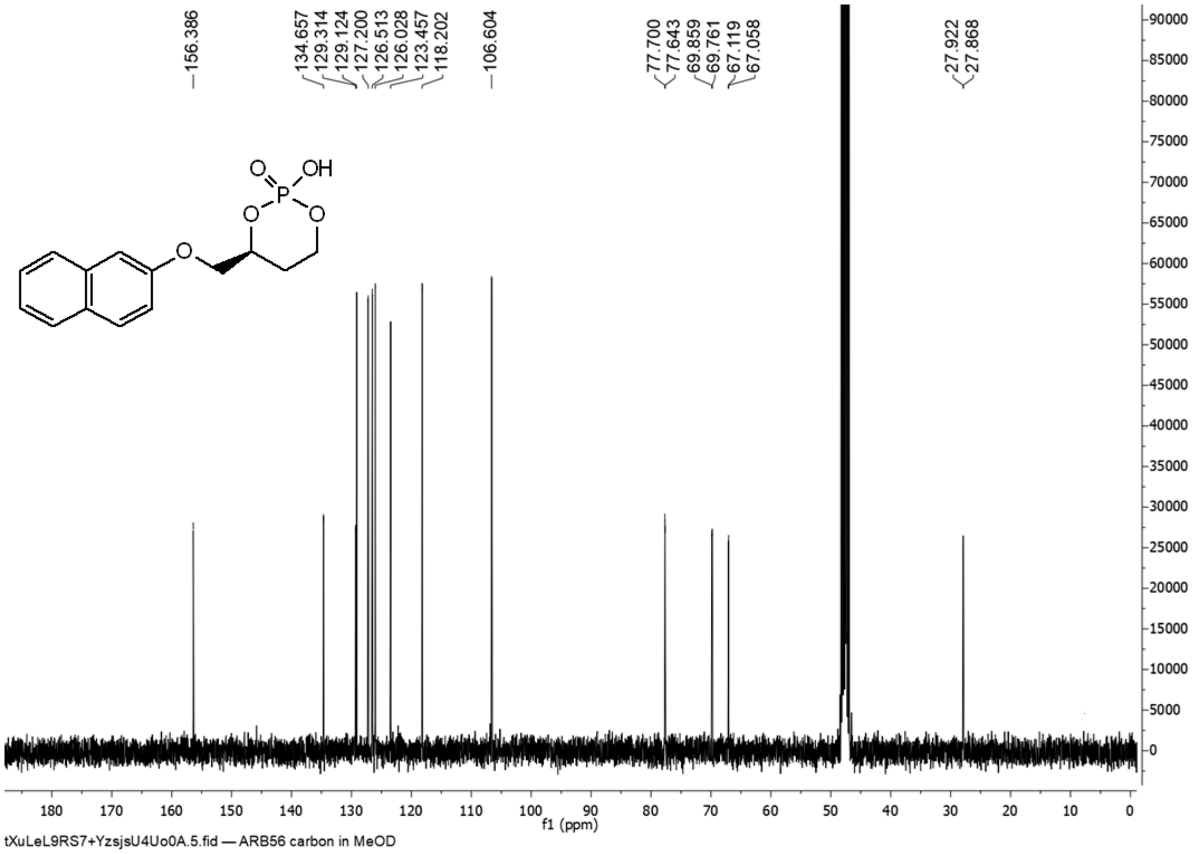

tXuLeL9RS7+YzsjsU4Uo0A.5.fid — ARB56 carbon in MeOD

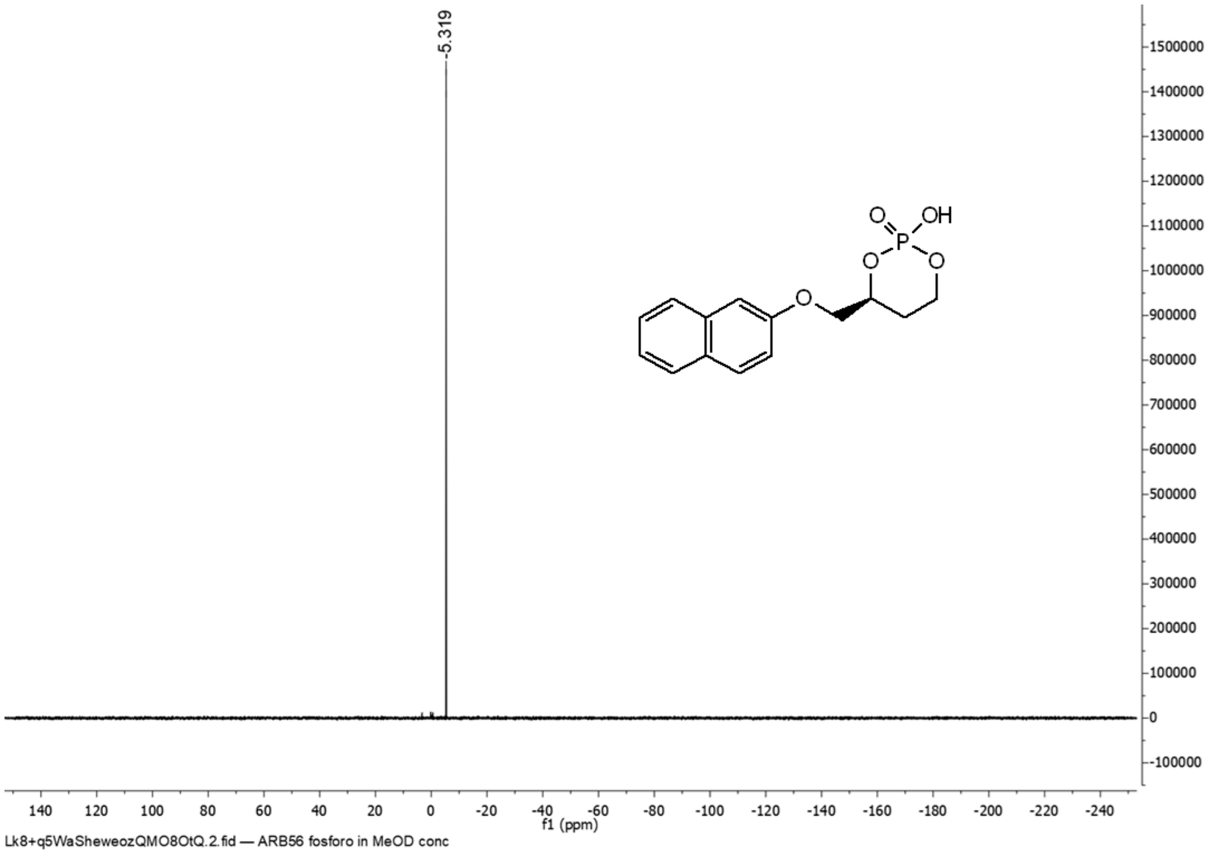

Lk8+q5WaSheweozQMO8OtQ.2.fid — ARB56 fosforo in MeOD conc

5. Physical and spectroscopic data of compounds 11.

(+)-1,3-Diphenyl-3-(phenylamino)propan-1-one (11a): 255 mg (85% yield). White solid, mp 167–168°C (crystallized from MeOH. Lit.[44] 168–169°C). [α]D20= 12.57 (c=0.15 in $CHCl_3$);^{1}H-NMR ($CDCl_3$, 400 MHz): 7.91 (d, *J*=7.2 Hz, 2H), 7.59–7.55 (m, 1H), 7.48–7.42 (m, 4H), 7.35–7.31 (m, 2H), 7.28–7.24 (m, 1H), 7.15–7.11 (m, 2H), 6.78–6.74 (m, 1H), 6.70–6.68 (m, 2H), 5.04 (t, *J*=6.4 Hz, 1H), 3.66-3.52 (m, 2H). ^{13}C-NMR ($CDCl_3$, 100 MHz): 197.9, 145.4, 141.7, 136.6, 133.4, 129.1, 128.8, 128.7, 128.2, 127.6, 126.7, 119.2, 115.2, 56.2, 45.7. IR (neat) ν=3385 (NH), 1672 cm^{-1} (CO). MS: *m/z* 209 (molecular ion $M^{+\cdot}$ *m/z* 301 is not detectable. 209 arises from the loss of PhNH· (92) from $M^{+\cdot}$). 90.3% ee, t_R=8.7267 min (minor), t_R=11.2067 min (major).

(+)-3-((2-Nitrophenyl)amino)-1,3-diphenylpropan-1-one (11b): 301 mg (87% yield). Yellow solid, mp 100–101°C (crystallized from MeOH. Lit.[45] 101–103°C). [α]D20= 15.81(c=0.15 in $CHCl_3$). ^{1}H-NMR ($CDCl_3$, 400 MHz): 8.17 (d, *J*=8.4 Hz, 1H), 7.94 (d, *J*=7.2 Hz, 2H), 7.61–7.57 (m, 1H), 7.49–7.45 (m, 4H), 7.38–7.28 (m, 4H), 6.82 (d, *J*=8.8 Hz, 1H), 6.66 (t, *J*=7.6 Hz, 1H), 5.39 (t, *J*=6.4 Hz, 1H), 3.67 (dd, , J_1=16.8 Hz; J_2=7.6 Hz, 1H), 3.54 (dd, J_1=16.8 Hz; J_2=7.6 Hz, 1H). ^{13}C-NMR ($CDCl_3$, 100 MHz): 196.6, 144.3, 141.5, 136.5, 136.1, 133.7, 129.7, 129.1, 128.7, 128.1, 127.8, 126.7, 126.3, 116.0, 115.5, 53.5, 46.7. IR (neat) ν=3399 (NH), 1678 cm^{-1} (CO). MS: *m/z* 209 (molecular ion $M^{+\cdot}$ *m/z* 346 is not detectable. 209 arises from the loss of 2-$NO_2C_6H_4NH$· (137) from $M^{+\cdot}$) 91.5% ee, t_R=10.3833 min (minor), t_R=13.5333 min (major).

(+)-3-((3-Fluorophenyl)amino)-1,3-diphenylpropan-1-one (11c): 290 mg (91% yield). White solid, mp 113–114°C (crystallized from MeOH. [α]D20= 13.72 (c=0.15 in $CHCl_3$). ^{1}H-NMR ($CDCl_3$, 400 MHz): 7.92 (d, *J*=7.2 Hz, 2H), 7.64–7.57 (m, 1H), 7.46–7.45 (m, 4H), 7.37–7.33 (m, 2H), 7.28–7.27 (m, 1H), 7.10–7.03 (m, 1H), 6.41 (d, *J*=7.6 Hz, 2H), 6.32–6.30 (m, 1H), 5.03–4.99 (m, 1H), 3.61–3.47 (m, 2H). ^{13}C-NMR (CD_3COCD_3, 100 MHz): 196.7, 163.8 (d, *J*= 238.7 Hz), 149.34 (d, *J*=11.0 Hz), 137.1, 133.1, 130.1 (d, *J*= 10.2 Hz), 128.6, 128.5, 128.4, 128.1, 126.6, 125.9, 109.9, 103.1 (d, *J*=21.5 Hz), 100.1 (d, *J*=25.3 Hz), 54.1, 46.1 IR (neat) ν=3324 (NH), 1674 cm^{-1} (CO). Elemental analysis calcd (%) for $C_{21}H_{18}FNO$: C 78.98; H 5.68; found: C 78.45; H 5.25. MS: *m/z* 209 (molecular ion $M^{+\cdot}$ *m/z* 319 is not detectable. 209 arises from the loss of 3-FC_6H_4NH· (110) from $M^{+\cdot}$) 91.7% ee, t_R=9.2333 min (major), t_R=10.3333 min (minor).

(+)-1,3-Diphenyl-3-(p-tolylamino)propan-1-one (11d): 259 mg (82% yield). Pale brown solid, mp 168–169°C (crystallized from MeOH. Lit.[46] 167–168°C). [α]D20= 14.16 (c=0.15 in $CHCl_3$). ^{1}H-NMR ($CDCl_3$, 400 MHz): 7.91 (d, *J*=7.2 Hz, 2H), 7.59–7.55 (m, 1H), 7.49–7.42 (m, 4H), 7.34–7.25 (m, 3H), 6.94 (d, *J*=8.0 Hz, 2H), 6.65 (d, *J*=8.0 Hz, 2H), 5.02 (t, *J*=6.4 Hz, 1H), 3.67–3.63 (m, 2H), 2.21 (s, 3H). ^{13}C-NMR (CD_3COCD_3, 100 MHz): 197.8, 142.2, 141.3, 136.6, 133.4, 129.7, 128.8, 128.6, 128.2, 127.7, 126.9, 115.9, 56.9, 45.4, 20.5. IR (neat) ν=3339 (NH), 1671 cm^{-1} (CO). MS: *m/z* 209 (molecular ion $M^{+\cdot}$ *m/z* 315 not detectable. 209 arises from the loss of 4-MeC_6H_4NH· (106) from $M^{+\cdot}$).92.9% ee, t_R=9.600 min (minor), t_R=12.4167 min (major).

(+)-3-((4-Nitrophenyl)amino)-1,3-diphenylpropan-1-one (11e): 315 mg (91% yield). Yellow solid, mp 186–187°C (crystallized from MeOH. Lit.[47] 186–187°C). [α]D20=16.77 (c=0.15 in $CHCl_3$). ^{1}H-NMR ($CDCl_3$, 400 MHz): 7.94 (d, *J*=8.8 Hz, 2H), 7.81 (d, *J*=7.6 Hz, 2H), 7.52–7.48 (m, 1H), 7.39–7.35 (m, 2H), 7.32–7.25 (m, 4H), 7.21–7.19 (m, 1H), 6.48 (d, *J*=8.8 Hz, 2H), 5.02 (t, *J*=6.4 Hz, 1H), 3.48–3.47 (m, 2H).^{13}C-NMR (CD_3COCD_3, 100 MHz): 197.7, 151.2, 140.6, 139.2, 136.4, 133.9, 129.1, 128.8, 128.2, 128.1, 126.4, 126.0, 113.2, 55.2, 45.2. IR (neat) ν=3351 (NH), 1673 cm^{-1} (CO). MS: *m/z* 209 (molecular ion $M^{+\cdot}$ *m/z* 346 is not detectable. 209 arises from the loss of 4-$NO_2C_6H_4NH$· (137) from $M^{+\cdot}$). 95.0% ee, t_R=18.0833 min (minor), t_R=22.4833 min (major).

(+)-3-((4-Bromophenyl)amino)-1,3-diphenylpropan-1-one (11f): 349 mg (92% yield). White solid, mp 181–182°C (crystallized from MeOH. Lit.[47] 183 °C). [α]D_{20}= 16.12 (c=0.15 in $CHCl_3$). ^{1}H-NMR ($CDCl_3$, 400 MHz): 7.90 (d, *J*=7.6 Hz, 2H), 7.59–7.58 (d, 2H), 7.56–7.49 (m, 2H), 7.46–7.43 (m, 2H), 7.41–7.38 (m, 2H), 7.31–7.26 (m, 3H), 6.77 (d, *J*=8.8 Hz, 1H), 5.01 (t, *J*=6.4 Hz, 1H), 3.89–3.77 (m, 2H).^{13}C-NMR (CD_3COCD_3, 100 MHz): 197.5, 144.9, 138.2, 136.3, 132.1, 130.6, 129.0, 128.7, 128.5, 128.2, 127.0, 122.1, 118.4, 57.9, 44.7. IR (neat) ν=3318 (NH), 1669 cm^{-1} (CO). MS: *m/z* 209 (molecular ion $M^{+\cdot}$ *m/z* 379; M+· + 2 *m/z* 381 is not detectable. 209 arises from the loss of 4-$BrC_6H_4NH^\cdot$ (170; 172) from $M^{+\cdot}$). 93.1% ee, t_R=12.7667 min (minor), t_R=16.5167 min (major).

(+)- 3-((2,6-Dimethylphenyl)amino)-1,3-diphenylpropan-1-one (11g): 270 mg (82% yield). Grey solid, mp 121–122°C (crystallized from MeOH). [α]D_{20}= 13.11 (c=0.15 in $CHCl_3$). ^{1}H-NMR ($CDCl_3$, 400 MHz): 7.95–7.94 (m, 2H), 7.59–7.57 (m, 1H), 7.50–7.47 (m, 2H), 7.36–7.35 (m, 2H), 7.31–7.27 (m, 1H), 7.23–7.20 (m, 2H), 7.07–7.00 (m, 2H), 6.93–6.91 (m, 1H), 5.02 (t, *J*=6.4 Hz, 1H), 3.70-3.69 (m, 2H), 2.32 (s, 6H). ^{13}C-NMR ($CDCl_3$,100 MHz): 197.2, 144.5, 141.4, 137.0, 132.9, 129.8, 128.6, 128.5, 128.4, 128.3, 127.5, 126.1, 124.3, 54.5, 45.6, 21.6. IR (neat) ν=3322 (NH), 1670 cm^{-1} (CO). Elemental analysis calcd (%) for $C_{23}H_{23}NO$: C 83.85; H 7.04; found: C 83.48; H 7.18. MS: *m/z* 209 (molecular ion $M^{+\cdot}$ *m/z* 329 is not detectable. 209 arises from the loss of 2,6-$Me_2C_6H_3NH^\cdot$ (120) from $M^{+\cdot}$). 89.7% ee, t_R=8.5667 min (minor), t_R=11.2667 min (major).

(+)-3-(Benzylamino)-1,3-diphenylpropan-1-one (11h): 275 mg (87% yield). White solid, mp 71–72°C (crystallized from MeOH. Lit. [48] 73–74 °C). [α]D_{20}= 9.99 (c=0.15 in $CHCl_3$). ^{1}H-NMR ($CDCl_3$, 400 MHz): $CDCl_3$ 8.06–8.04 (m, 2H), 7.90–7.88 (m, 2H), 7.57–7.55 (m, 1H) 7.51–7.17 (m, 9H), 7.10–7.08 (m, 1H), 4.36 (t, *J*=6.4 Hz, 1H), 3.99–3.89 (m, 2H), 2.36 (s, 2H).^{13}C-NMR (CD_3COCD_3, 100 MHz): 197.4, 140.9, 140.6, 133.1, 131.9, 129.7, 128.9, 128.8, 128.6, 128.5, 128.0, 125.9, 126.3, 56.1, 44.6, 43.5. IR (neat) ν=3338 (NH), 1675 cm^{-1} (CO). MS: *m/z* 209 (molecular ion $M^{+\cdot}$ *m/z* 315 is not detectable. 209 arises from the loss of $C_6H_5CH_2NH^\cdot$ (106) from $M^{+\cdot}$).92.6% ee, t_R=7.500 min (major), t_R=9.3833 min (minor).

(+)-3-(3-Methoxyphenyl)-1-phenyl-3-(phenylamino)propan-1-one (11i): 304 mg (92% yield). White solid, mp 104–105°C (crystallized from MeOH. Lit. [49] 106–107 °C). [α]D_{20}= 18.71 (c=0.15 in $CHCl_3$). ^{1}H-NMR ($CDCl_3$, 400 MHz): 7.89–7.80 (m, 2H), 7.55–7.52 (m, 1H), 7.43–7.34 (m, 2H), 7.23–7.08 (m, 5H), 7.01–6.99 (m, 3H), 6.79–6.76 (m, 1H), 5.04 (t, *J*=6.4 Hz, 1H), 4.07–3.91 (m, 2H), 3.75 (s, 3H).^{13}C-NMR ($CDCl_3$, 100 MHz): 197.7, 159.9, 140.7, 136.5, 133.4, 129.8, 129.5, 129.2, 128.9, 128.7, 128.2, 125.9, 119.9, 119.3, 112.7, 55.3, 55.2, 45.2. IR (neat) ν=3324 (NH), 1680 cm^{-1} (CO). MS: *m/z* 239 (molecular ion $M^{+\cdot}$ *m/z* 331 is not detectable. 239 arises from the loss of $C_6H_5NH^\cdot$ (92) from $M^{+\cdot}$). 92.9% ee, t_R=12.9667 min (major), t_R=17.8333 min (minor).

(+)-1-Phenyl-3-(phenylamino)-3-(*p*-tolyl)propan-1-one (11k) 255 mg (81% yield). White solid, mp 128–129°C (crystallized from MeOH. Lit. [48] 129–130 °C). [α]D_{20}= 14.43 (c=0.15 in $CHCl_3$). ^{1}H-NMR ($CDCl_3$, 400 MHz): 7.88 (d, *J*=7.2 Hz, 2H), 7.56 (t, *J*=7.6 Hz, 1H), 7.42 (t, *J*=7.6 Hz, 1H), 7.36 (d, *J*=8.0 Hz, 1H), 7.17–7.10 (m, 5H), 6.86–6.79 (m, 2H), 5.02 (t, *J*=6.4 Hz, 1H), 3.73-3.66 (m, 2H), 2.31 (s, 3H).^{13}C-NMR ($CDCl_3$, 100 MHz): 198.1, 145.8, 139.0, 137.2, 136.7, 133.4, 129.5, 129.1, 128.7, 128.2, 126.5, 118.8, 114.9, 55.5, 45.9, 21.1. IR (neat) ν=3332 (NH), 1683 cm^{-1} (CO). MS: *m/z* 223 (molecular ion $M^{+\cdot}$ *m/z* 315 is not detectable. 223 arises from the loss of $C_6H_5NH^\cdot$ (92) from $M^{+\cdot}$). (92.9% ee, t_R=9.2333 min (major), t_R=10.2333 min (minor).

(+)-3-(4-Chlorophenyl)-1-phenyl-3-(phenylamino)propan-1-one (11l) 291 mg (87% yield). Grey solid, mp 115–116°C (crystallized from MeOH. Lit. [48] 117–118°C). [α]D_{20}= 12.98 (c=0.15 in

$CHCl_3$). ^{1}H-NMR ($CDCl_3$, 400 MHz): 7.93 (d, *J*=7.2 Hz, 2H), 7.85 (d, *J*=7.6 Hz, 1H), 7.70–7.66 (m, 1H), 7.54–7.48 (m, 3H), 7.44–7.41 (m, 3H), 7.31 (d, *J*=8.4 Hz, 2H) 7.17–7.15 (m, 2H), 4.90 (t, *J*=6.4 Hz, 1H), 4.04-3.91 (m, 2H). ^{13}C-NMR ($CDCl_3$, 100 MHz): 198.2, 143.3, 138.0, 136.5, 133.4, 132.9, 129.6, 129.3, 128.7, 128.5, 128.1, 122.5, 113.2, 53.8, 44.7. IR (neat) ν=3312 (NH), 1681 cm^{-1} (CO). MS: *m/z* 243 (molecular ion $M^{+\cdot}$ *m/z* 335 is not detectable. 243 arises from the loss of $C_6H_5NH^{\cdot}$ (92) from $M^{+\cdot}$). 92.6% ee, t_R=7.5000 min (major), t_R=9.3833 min (minor).

(+)-3-(4-Nitrophenyl)-1-phenyl-3-(phenylamino)propan-1-one (11m) 231 mg (67% yield). Pale yellow solid, mp 103–104°C (crystallized from MeOH. Lit. [48] 105–106°C). $[\alpha]D_{20}$= 13.43 (c=0.15 in $CHCl_3$). ^{1}H-NMR ($CDCl_3$, 400 MHz): 8.30 (d, *J*=8.4 Hz, 2H), 8.06 (d, *J*=8.4 Hz, 2H), 7.86–7.80 (m, 2H), 7.65–7.63 (m, 2H), 7.56 (t, *J*=7.6 Hz, 2H), 7.43–7.42 (m, 2H), 7.33–7.28 (m, 2H), 5.22 (t, *J*=6.4 Hz, 1H), 4.48–4.27 (m, 2H). ^{13}C-NMR ($CDCl_3$, 100 MHz): 197.6, 149.9, 148.6, 145.7, 137.6, 133.3, 129.0, 128.9, 128.8, 128.6, 124.2, 118.4, 113.1, 55.3, 45.1. IR (neat) ν=3342 (NH), 1677 cm^{-1} (CO). MS: *m/z* 254 (molecular ion $M^{+\cdot}$ *m/z* 346 is not detectable. 254 arises from the loss of $C_6H_5NH^{\cdot}$ (92) from $M^{+\cdot}$). 95.1% ee, t_R=19.5833 min (major), t_R=29.6833 min (minor).

(+)-1-Phenyl-3-(phenylamino)-3-(thiophen-2-yl)propan-1-one (11n) 275 mg (90% yield). Pale yellow solid, mp 103–104°C (crystallized from MeOH. Lit. [50] 105–106°C). $[\alpha]D_{20}$= 16.27 (c=0.15 in $CHCl_3$). ^{1}H-NMR ($CDCl_3$, 400 MHz): 7.95 (d, *J*=7.6 Hz, 2H),7.61–7.58 (m, 1H), 7.49–7.46 (m, 2H), 7.20–7.17 (m, 3H), 7.06–7.05 (m, 1H), 6.95–6.93 (m, 1H), 6.82–6.75 (m, 3H), 5.42 (t, *J*=6.4 Hz, 1H), 3.68-3.67 (m, 2H). ^{13}C-NMR ($CDCl_3$, 100 MHz): 197.7, 146.8, 145.8, 136.7, 133.5, 129.2, 128.7, 128.1, 126.9, 124.4, 124.3, 119.1, 114.7, 51.3, 45.7. IR (neat) ν=3331 (NH), 1674 cm^{-1} (CO). MS: *m/z* 223 (molecular ion $M^{+\cdot}$ *m/z* 315 is not detectable. 223 arises from the loss of $C_6H_5NH^{\cdot}$ (92) from $M^{+\cdot}$). 89.6% ee, t_R=9.3167 min (minor), t_R=11.1000 min (major).

(+)-1-(3-Methoxyphenyl)-3-phenyl-3-(phenylamino)propan-1-one (11o) 308 mg (93% yield). White solid, mp 102–103°C (crystallized from MeOH. Lit. [49] 105–106°C). $[\alpha]D_{20}$= 15.5 (c=0.15 in $CHCl_3$). ^{1}H-NMR ($CDCl_3$, 400 MHz): 7.47–7.44 (m, 4H), 7.33–7.23 (m, 4H), 7.15–7.11 (m, 3H), 6.83–6.73 (m, 3H), 5.04 (t, *J*=6.4 Hz, 1H), 3.83 (s, 3H), 3.76–3.64 (m, 2H). ^{13}C-NMR ($CDCl_3$, 100 MHz): 197.3, 159.9, 140.7, 137.9, 129.7, 129.5, 129.2, 128.9, 128.8, 127.8, 127.0, 125.9, 120.9, 120.2, 112.2, 55.5, 55.4, 45.3. IR (neat) ν=3300 (NH), 1669 cm^{-1} (CO). MS: *m/z* 239 (molecular ion $M^{+\cdot}$ *m/z* 331 is not detectable. 239 arises from the loss of $C_6H_5NH^{\cdot}$ (92) from $M^{+\cdot}$). 90.1% ee, t_R=12.9000 min (major), t_R=18.2833 min (minor).

(+)-1-(4-Chlorophenyl)-3-phenyl-3-(phenylamino)propan-1-one (11q) 308 mg (92% yield). White solid, mp 119–120°C (crystallized from MeOH. Lit. [48] 118–119°C). $[\alpha]D_{20}$= 14.43 (c=0.15 in $CHCl_3$). ^{1}H-NMR ($CDCl_3$, 400 MHz): 7.95–7.94 (d, *J* = 8 Hz, 2H), 7.67–7.02 (m, 12H) 5.03 (t, *J*=6.4 Hz, 1H), 3.59–3.58 (m, 2H). ^{13}C-NMR (CD_3OD, 100 MHz): 197.3, 145.2, 142.1, 140.3, 135.5, 129.7, 128.7, 128.4, 128.1, 125.5, 121.6, 117.6, 114.2, 54.8, 45.8. IR (neat) ν=3341 (NH), 1677 cm^{-1} (CO). MS: *m/z* 243 (molecular ion $M^{+\cdot}$ *m/z* 335 is not detectable. 243 arises from the loss of $C_6H_5NH^{\cdot}$ (92) from $M^{+\cdot}$). 92.8% ee, t_R=6.1000 min (minor), t_R =7.3000 min (major).

References

[44] Hu, W.; Xiang, J.; Zhou, Q.; Gao, X. Harnessing Protonated 2,2′-Bipyridinium Salts as Powerful Brønsted Acid Catalysts in Organic Reactions. *J. Org. Chem*. **2023**, *88*, 4066−4076. https://doi.org/10.1021/acs.joc.2c02239.

[45] Kaur Rajput, J.; Kaur, G. $Bi(NO_3)_3 \cdot 5H_2O$: An Efficient and Green Catalyst for Synthesis of1,5-Benzodiazepines and β-Amino Carbonyl Compounds. *Asian J. Chem.* **2013**, *25,* 6545−6549 https://doi.org/10.14233/ajchem.2013.14353.

[46] Li, H.; Zeng, H.-Y.; Shao, H.-W. Bismuth(III) chloride-catalyzed one-pot Mannich reaction: three-component synthesis of β-amino carbonyl compounds. *Tetrahedron Lett.* **2009,** *50,* 6858−6860. https://doi.org/10.1016/j.tetlet.2009.09.131.

[47] Blatt, A.H.; Gross, N. The Addition of Ketones to Schiff Bases. *J. Org. Chem.* **1964,** *29,* 3306–3311. https://doi.org/10.1021/jo01034a044.

[48] Movassagh, B.; Khosousi, S. K_3PO_4-catalyzed one-pot synthesis of β-amino ketones. *Monatsh. Chem*. **2012,** *143*, 1503–1506. https://doi.org/10.1007/s00706-012-0729-1.

[49] Wu, Y.; Chen, C.; Jia, G.; Zhu, X.; Sun, H.; Zhang, G.; Zhang, W.; Gao, Z. Salicylato Titanocene Complexes as Cooperative Organometallic Lewis Acid and Brønsted Acid Catalysts for Three-Component Mannich Reactions. *Chem. Eur. J.* **2014**, *20*, 8530–8535. https://doi.org/10.1002/chem.201402438.

[50] Akiyama, T.; Takaya, J.; Kagoshima, H. Brønsted Acid-Catalyzed Mannich-Type Reactions in Aqueous Media. *Adv. Synth. Catal.* **2002**, *344*, 338–347. https://doi.org/10.1002/1615-4169(200206)344:3/4<338::AID-ADSC338>3.0.CO;2-O.

6. NMR spectra of compounds 11.

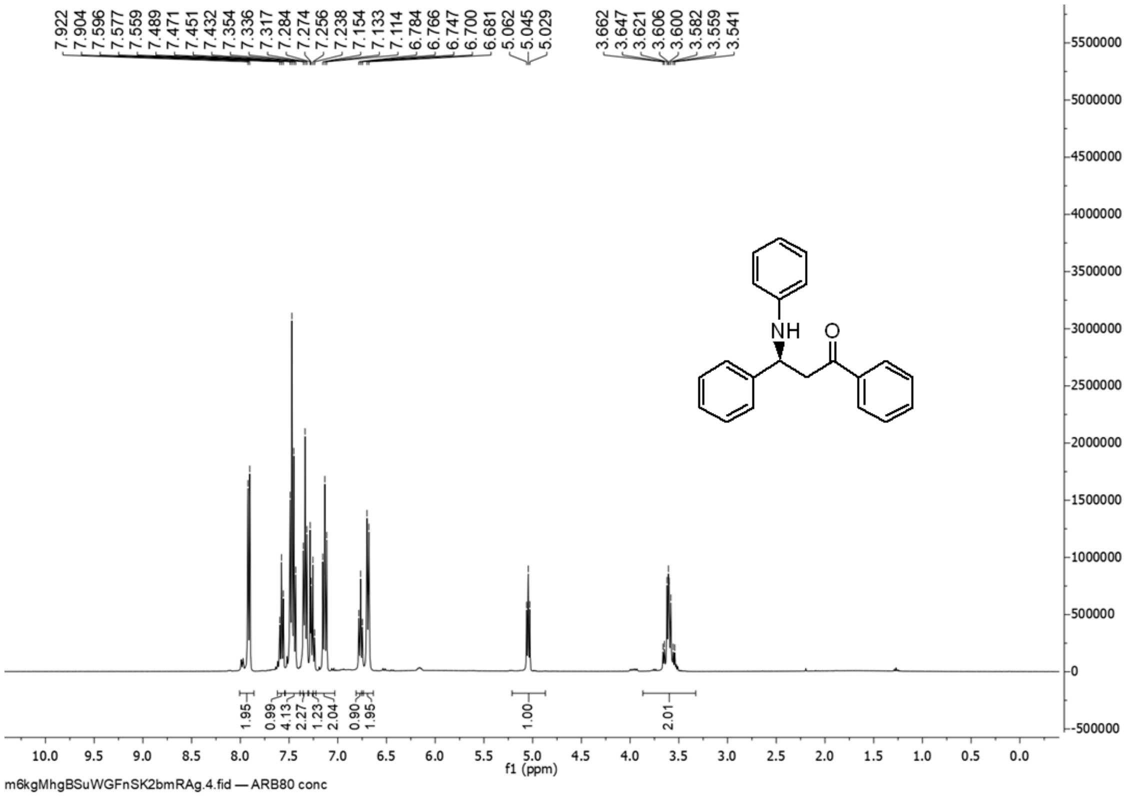

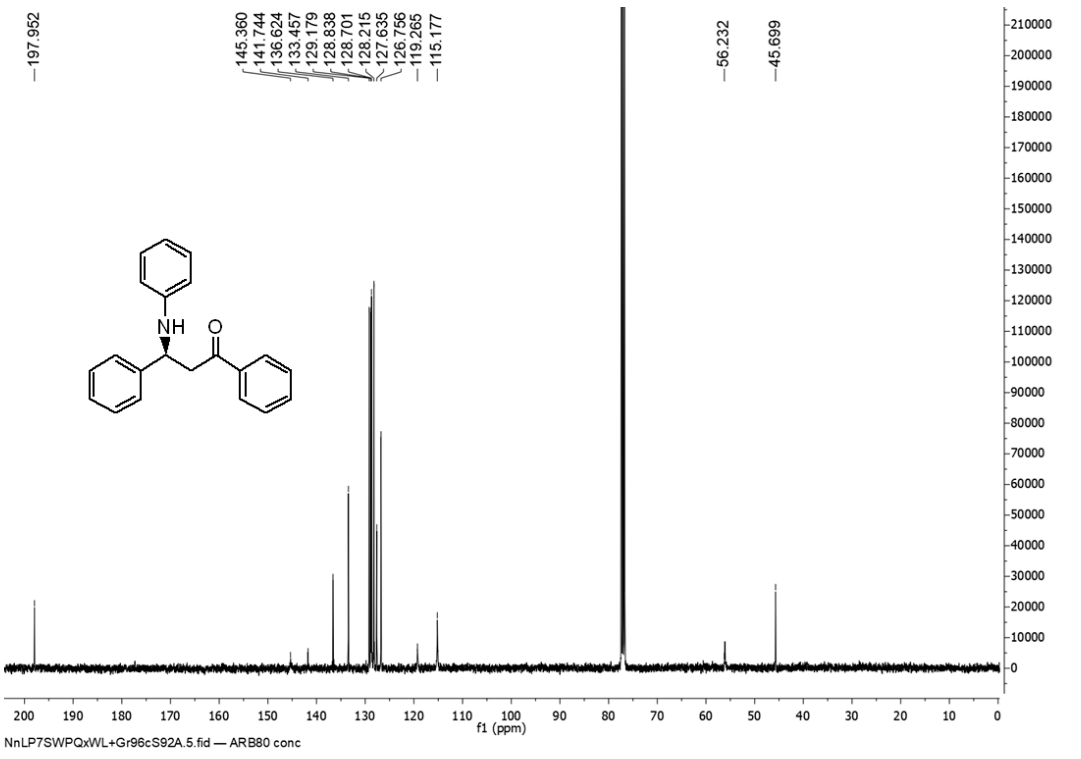

(+)-1,3-Diphenyl-3-(phenylamino)propan-1-one (11a).

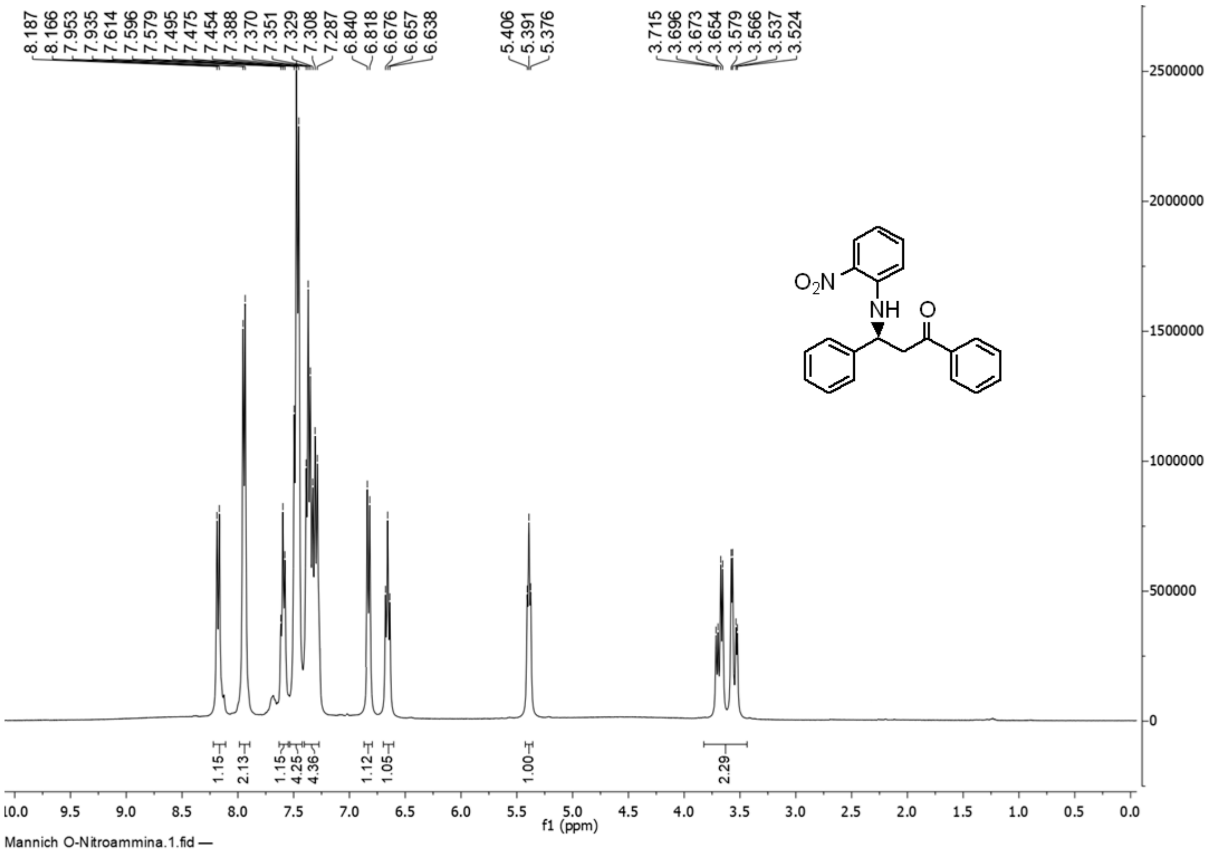

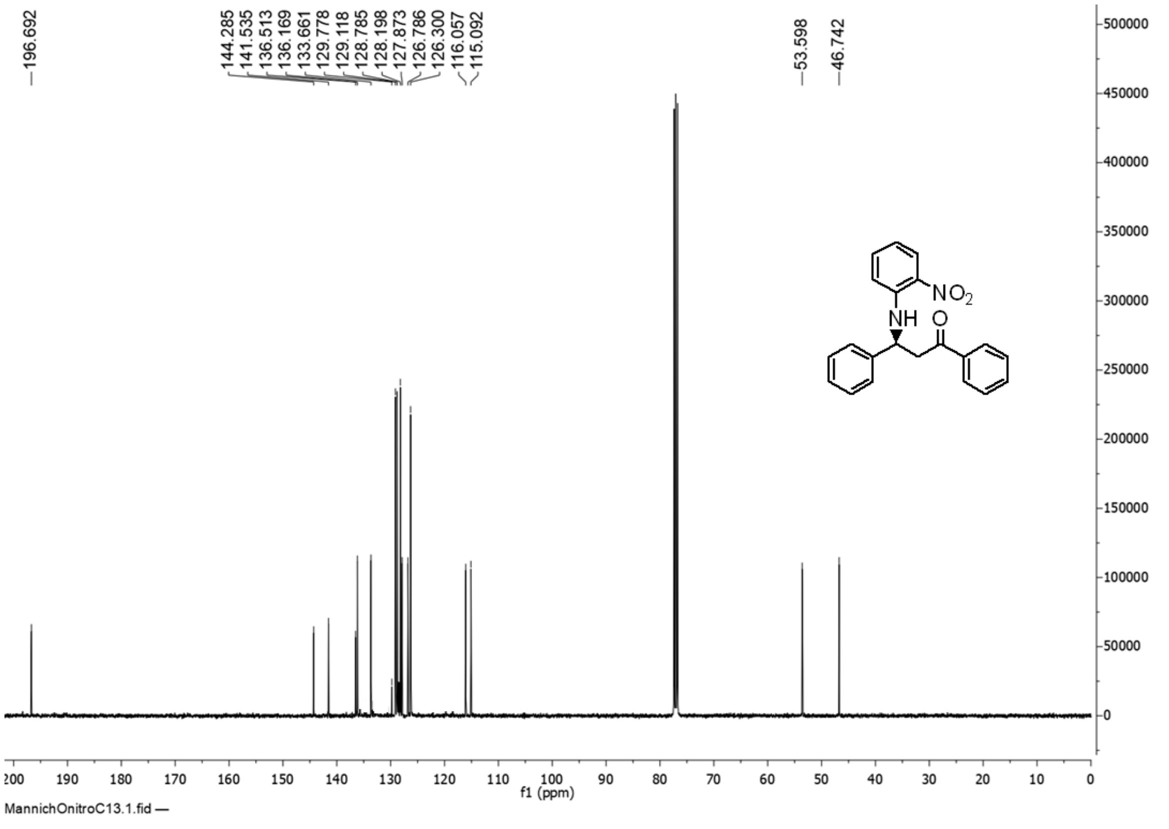

(+)-3-((2-Nitrophenyl)amino)-1,3-diphenylpropan-1-one (11b).

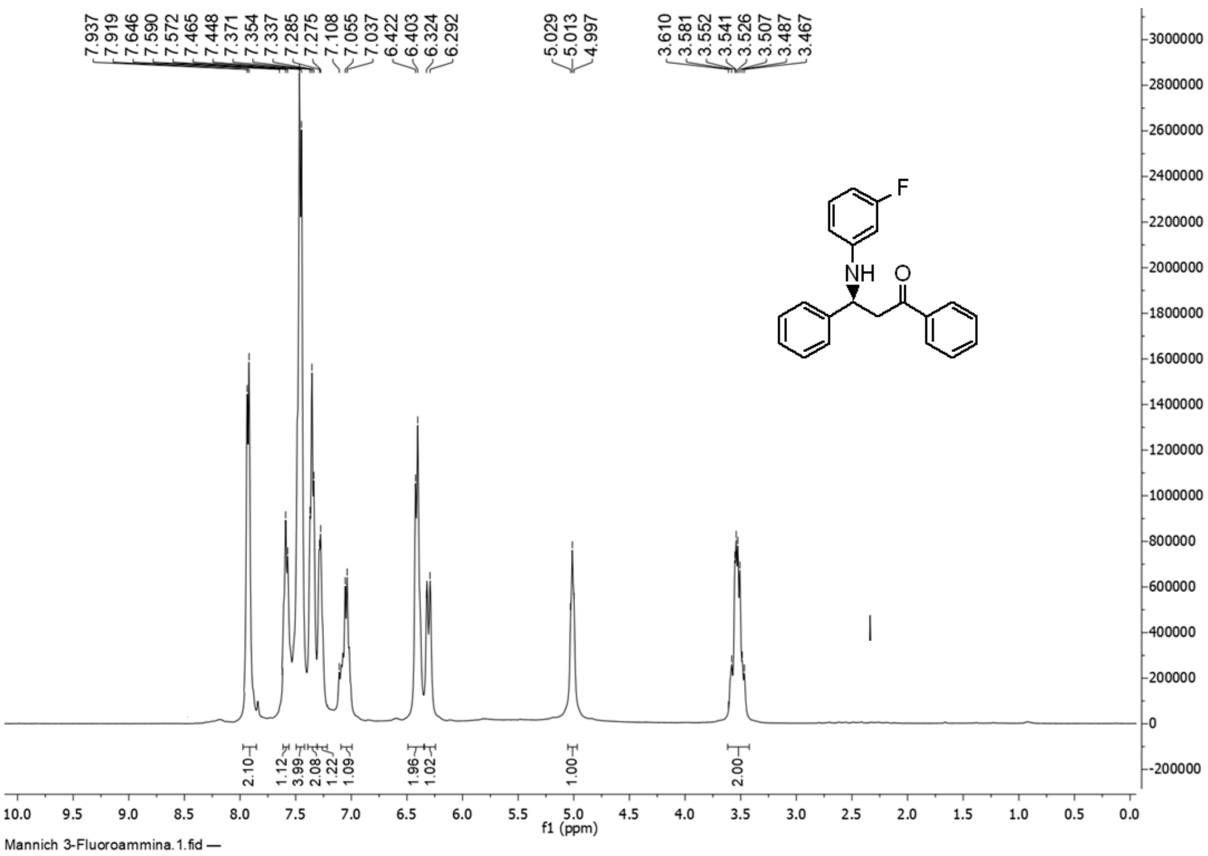

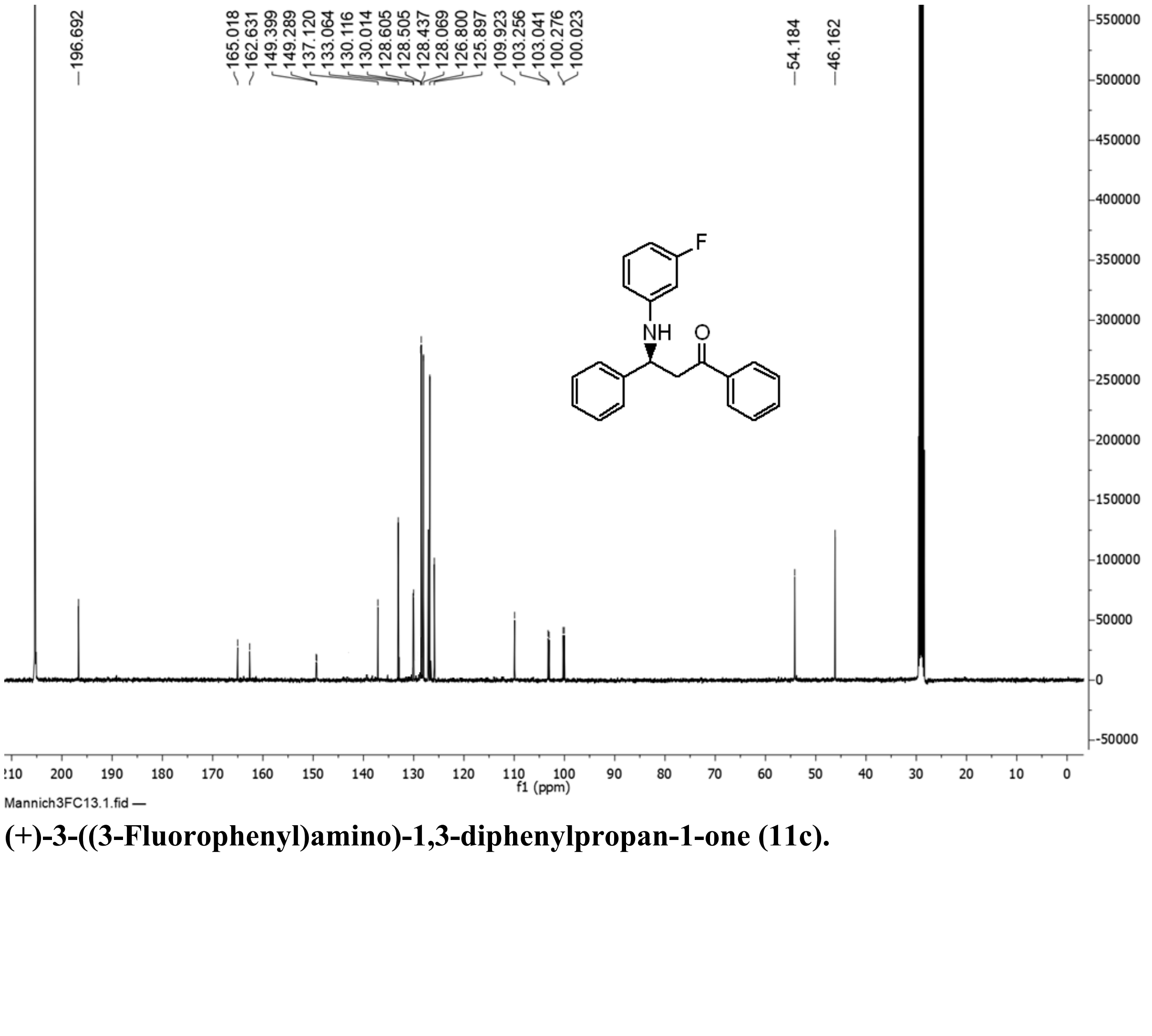

(+)-3-((3-Fluorophenyl)amino)-1,3-diphenylpropan-1-one (11c).

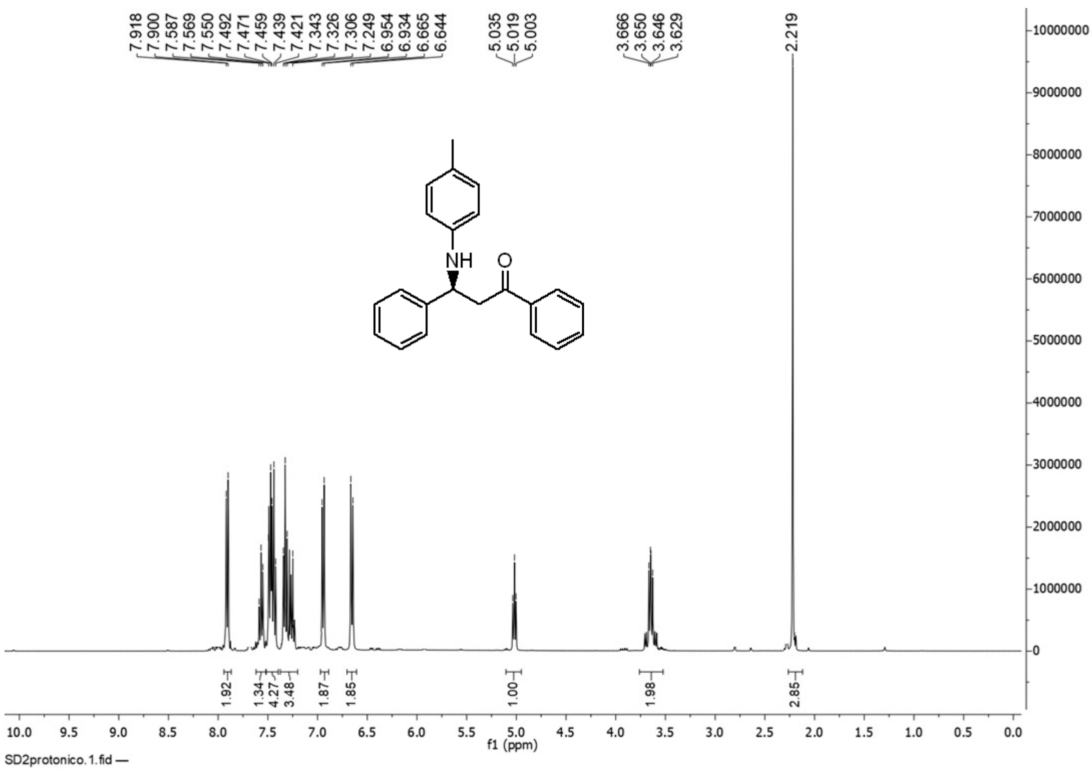

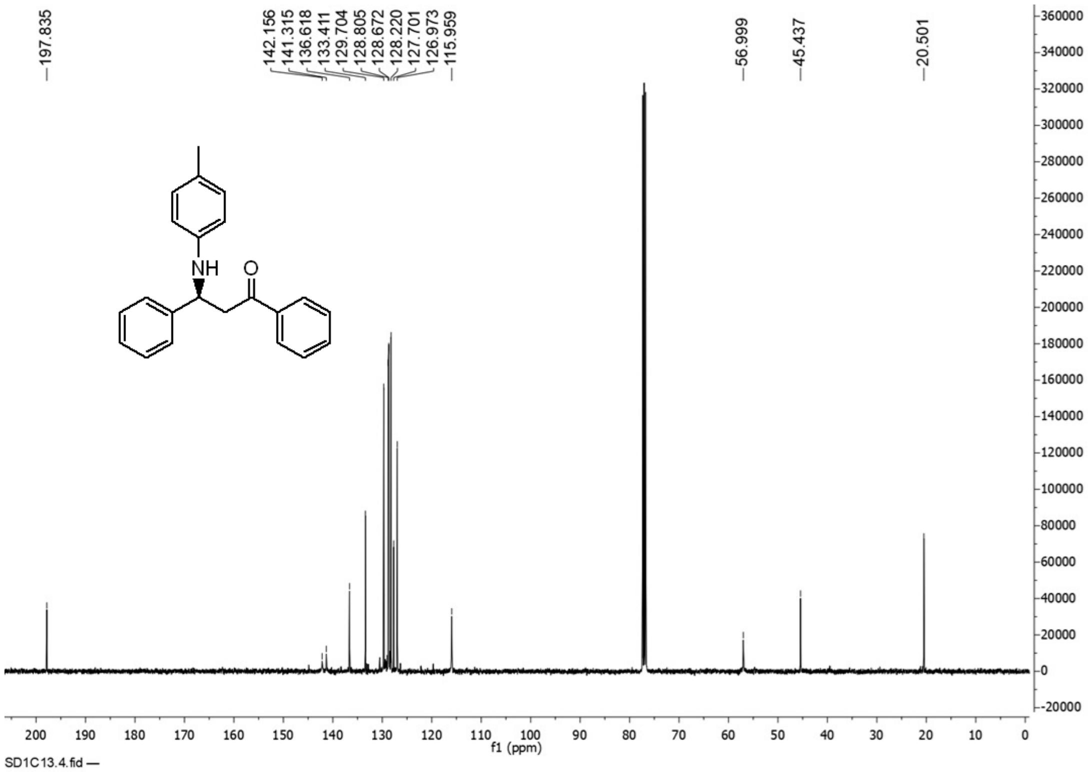

(+)-1,3-Diphenyl-3-(p-tolylamino)propan-1-one (11d).

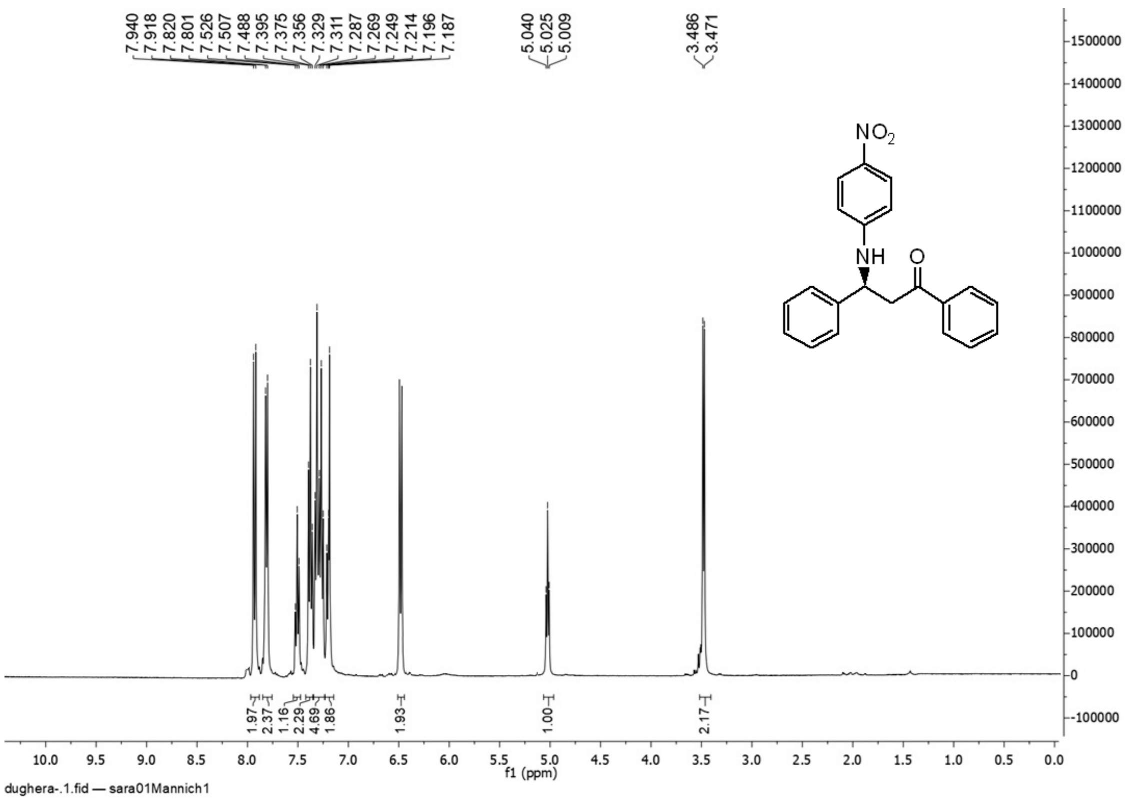

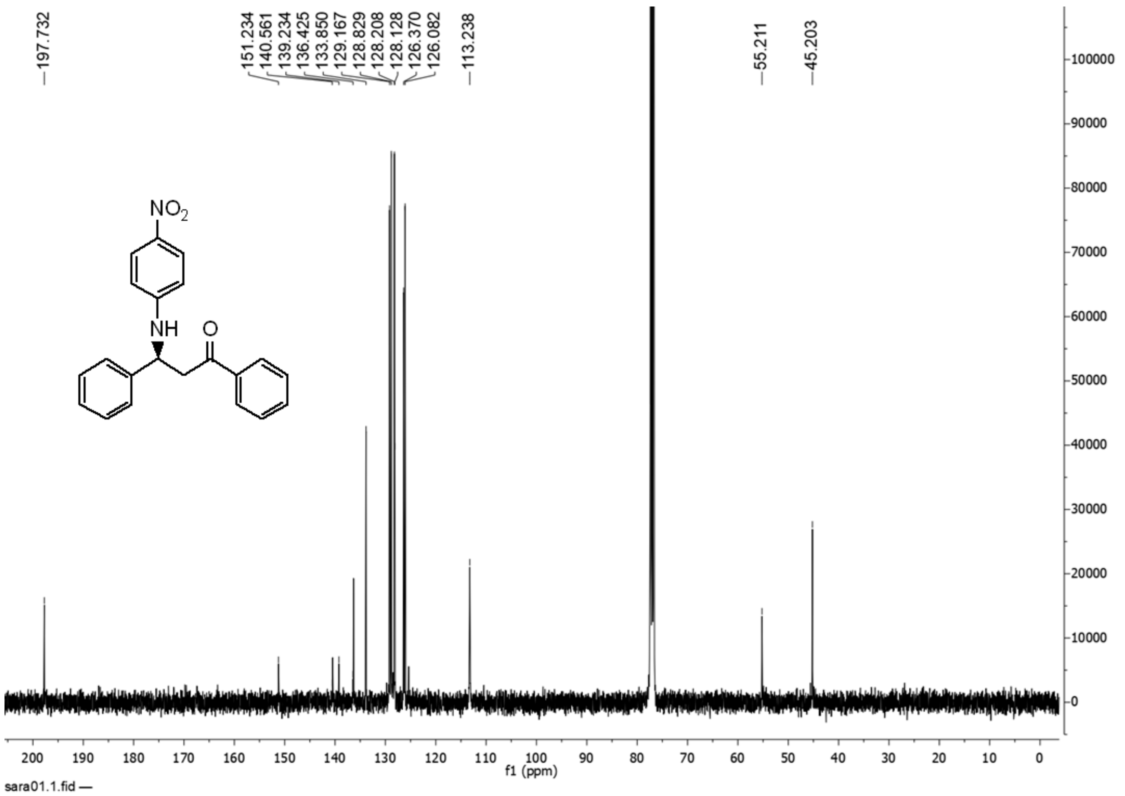

(+)-3-((4-Nitrophenyl)amino)-1,3-diphenylpropan-1-one (11e).

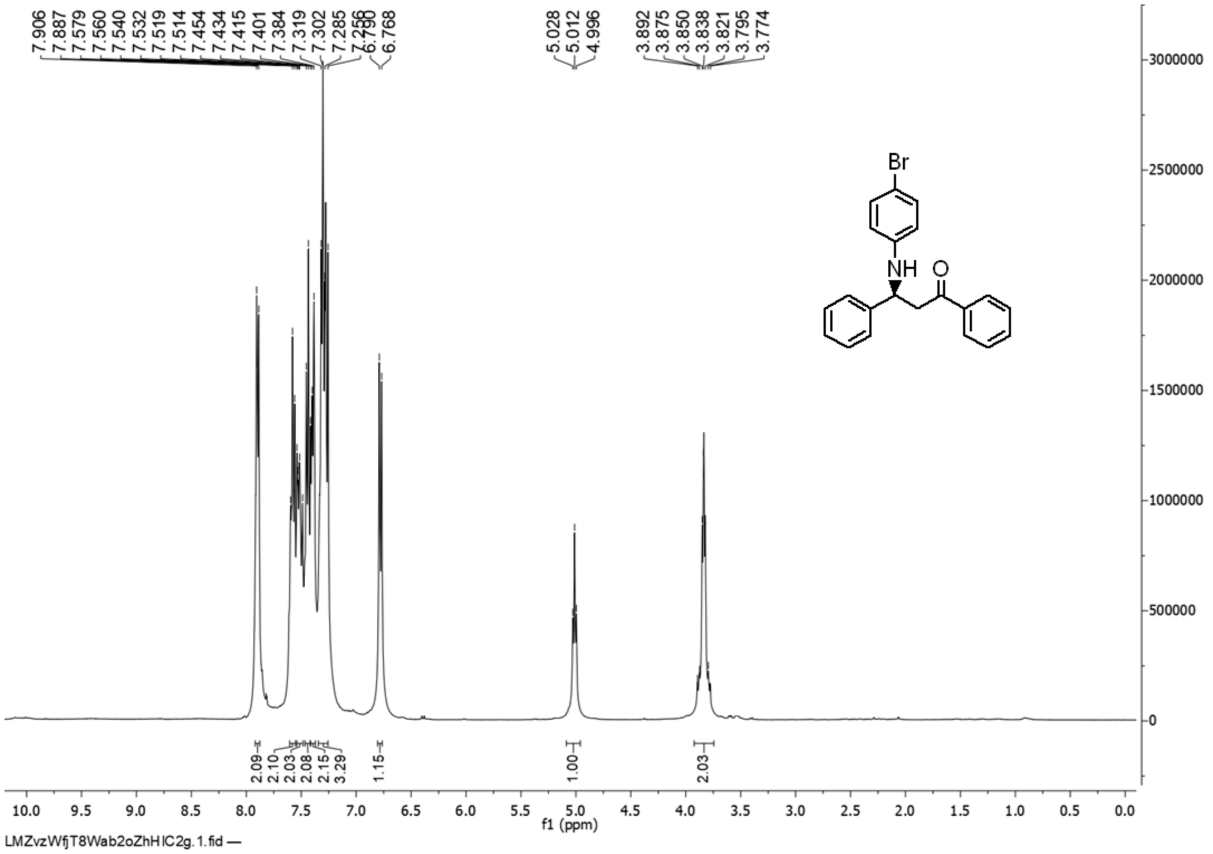

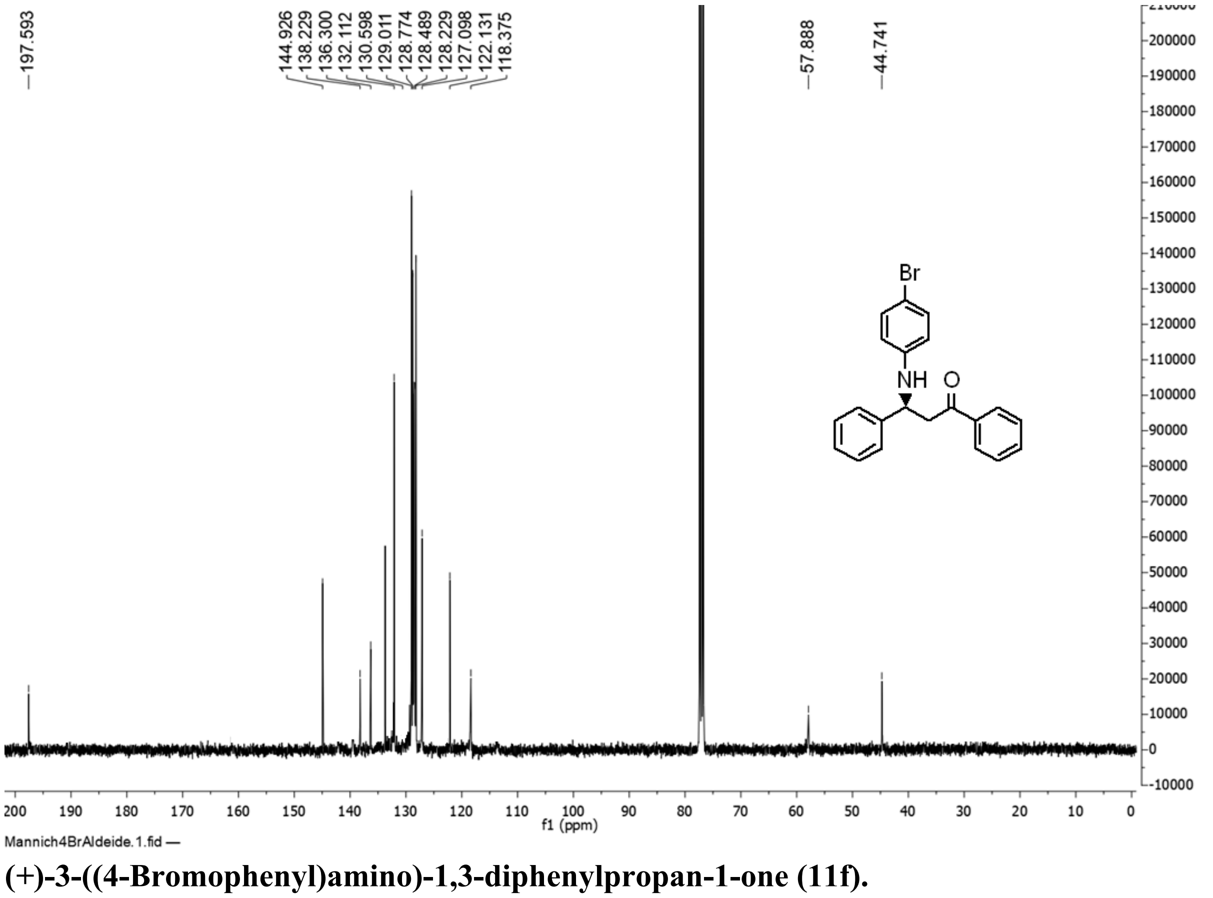

(+)-3-((4-Bromophenyl)amino)-1,3-diphenylpropan-1-one (11f).

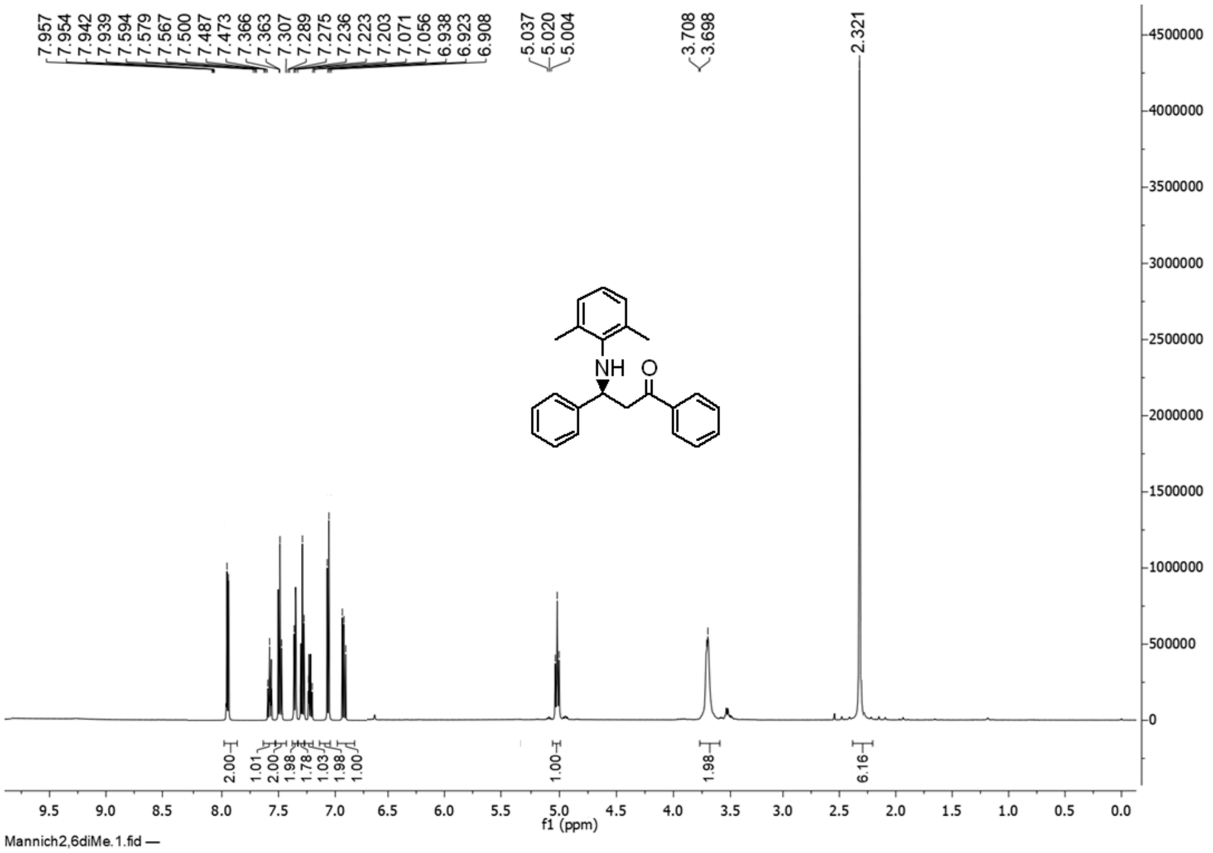

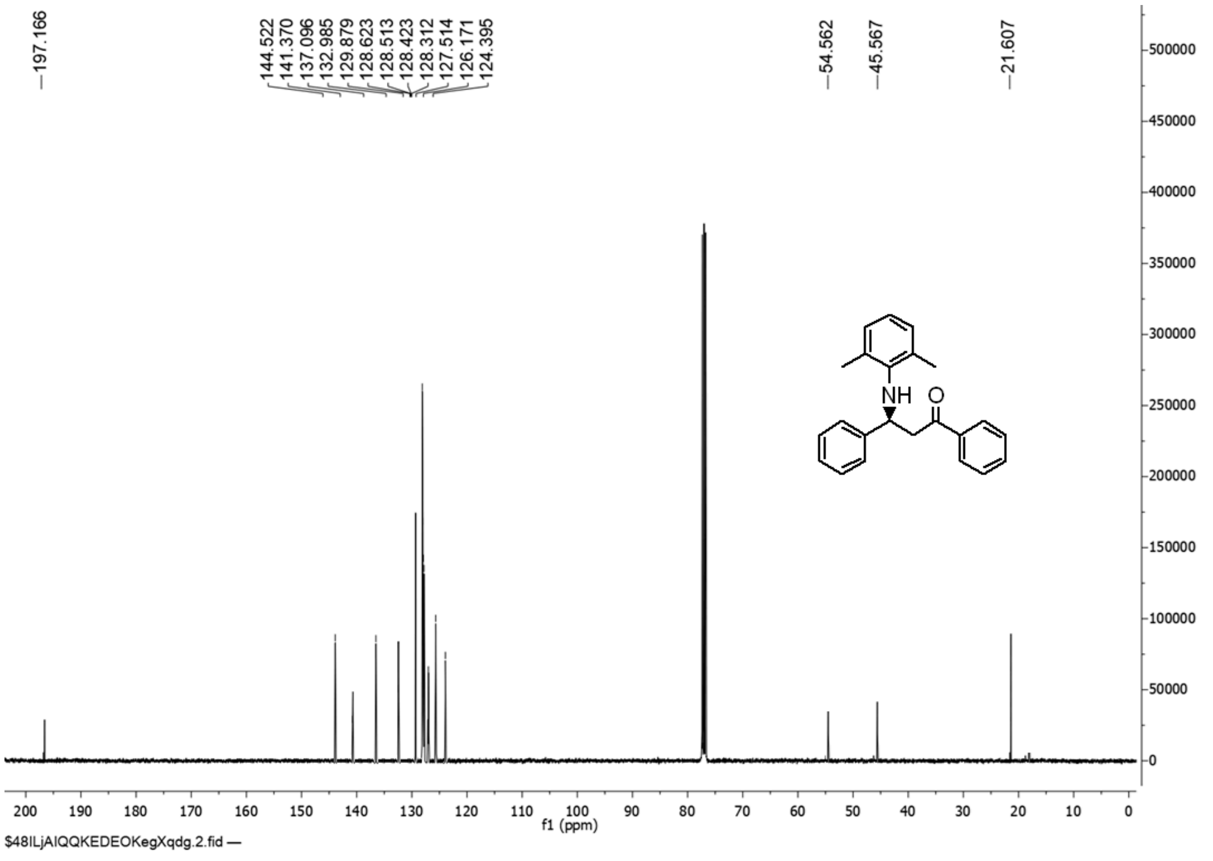

(+) 3-((2,6-Dimethylphenyl)amino)-1,3-diphenylpropan-1-one (11g).

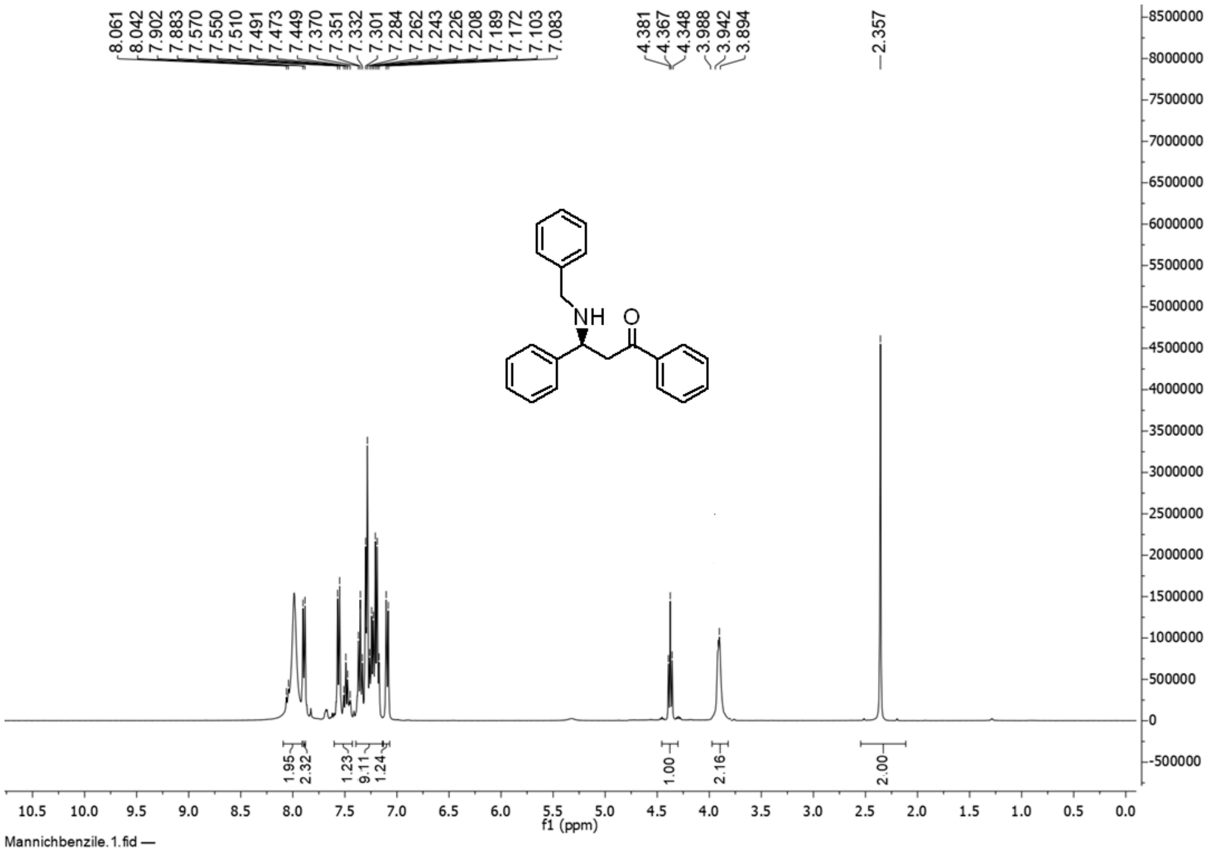

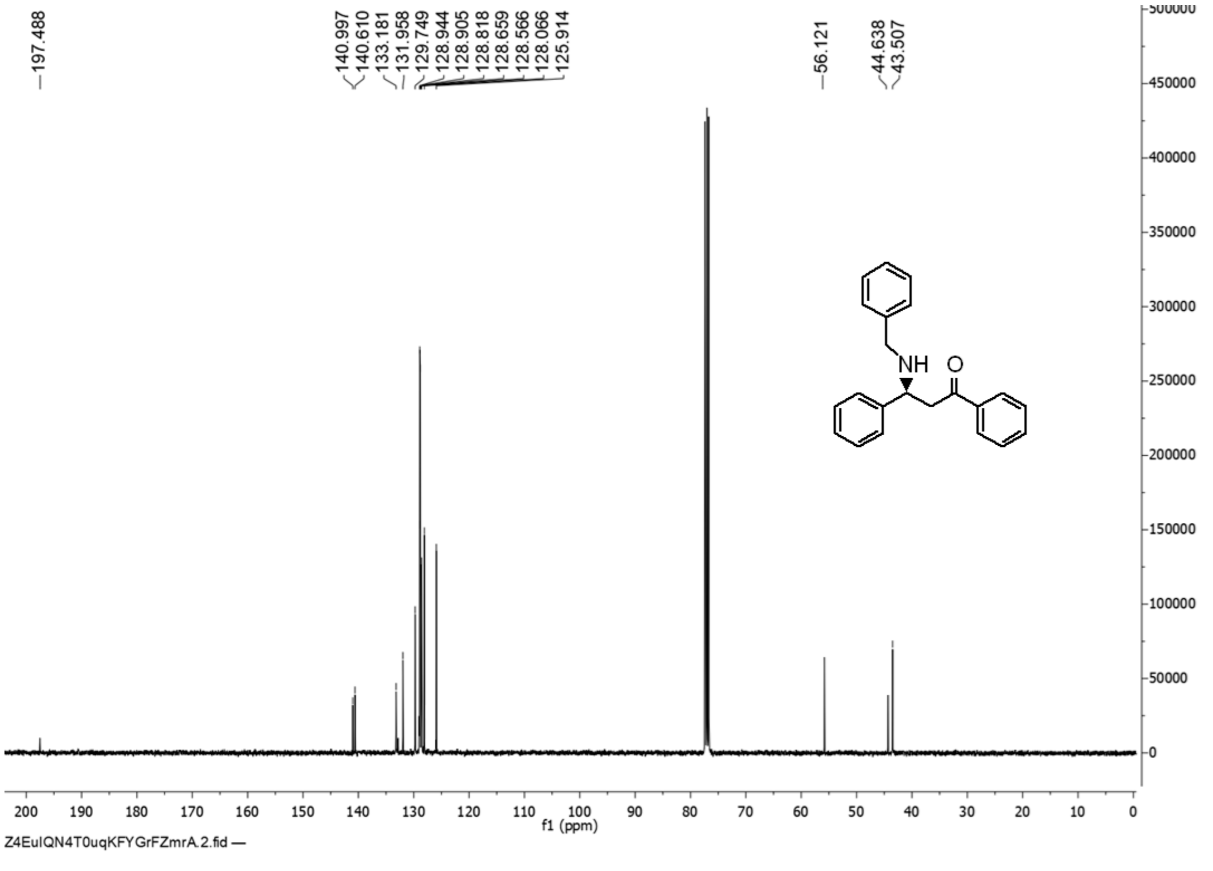

(+)-3-(Benzylamino)-1,3-diphenylpropan-1-one (11h).

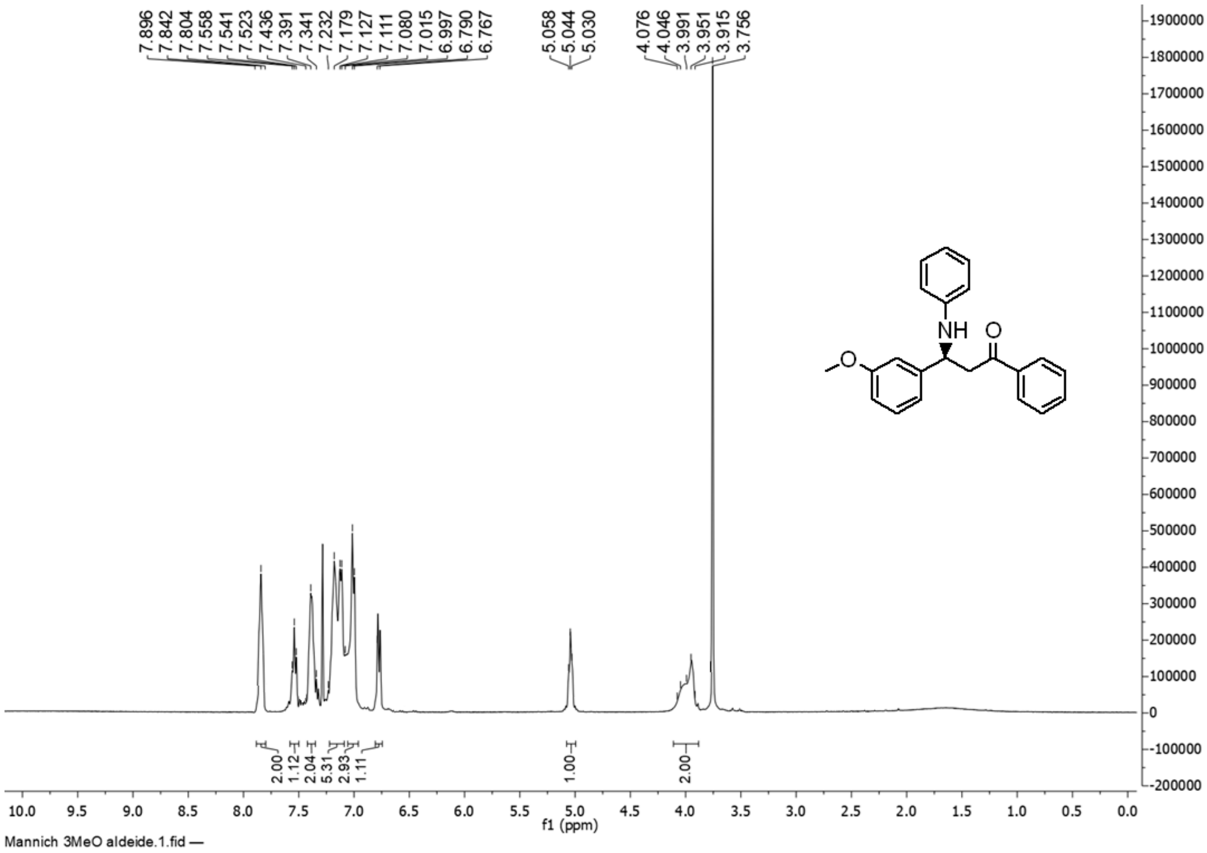

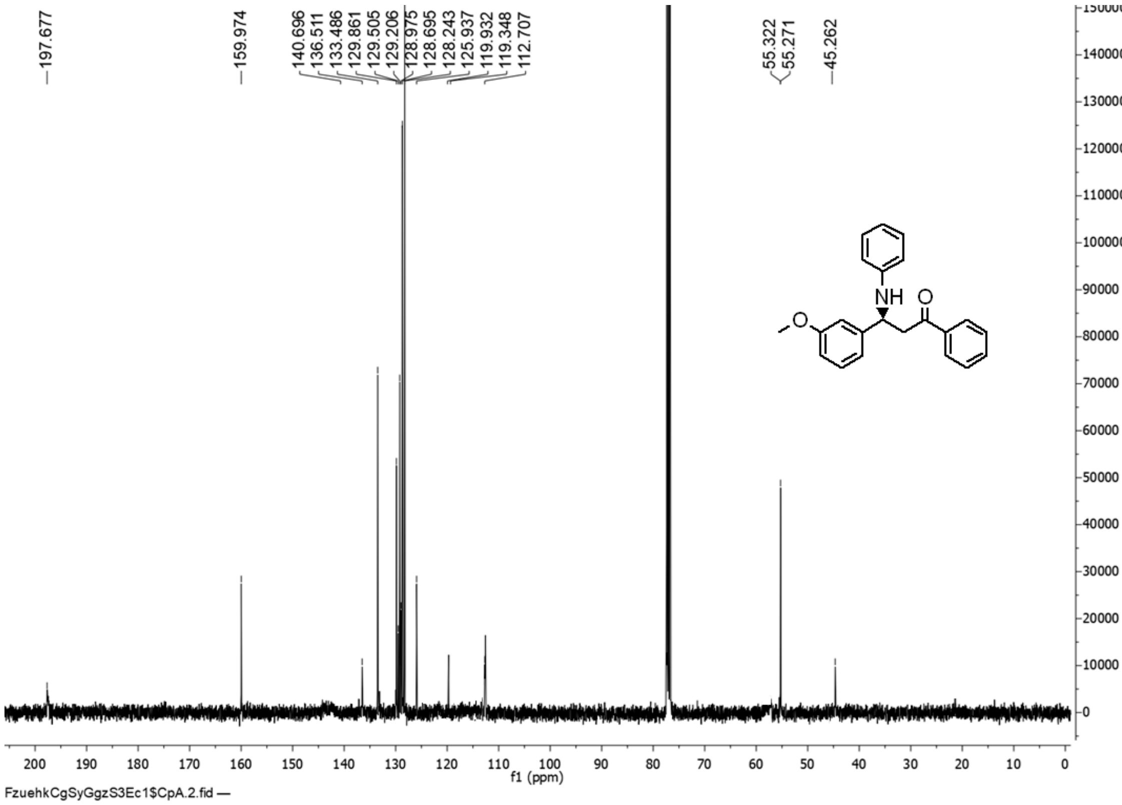

(+)-3-(3-Methoxyphenyl)-1-phenyl-3-(phenylamino)propan-1-one (11i).

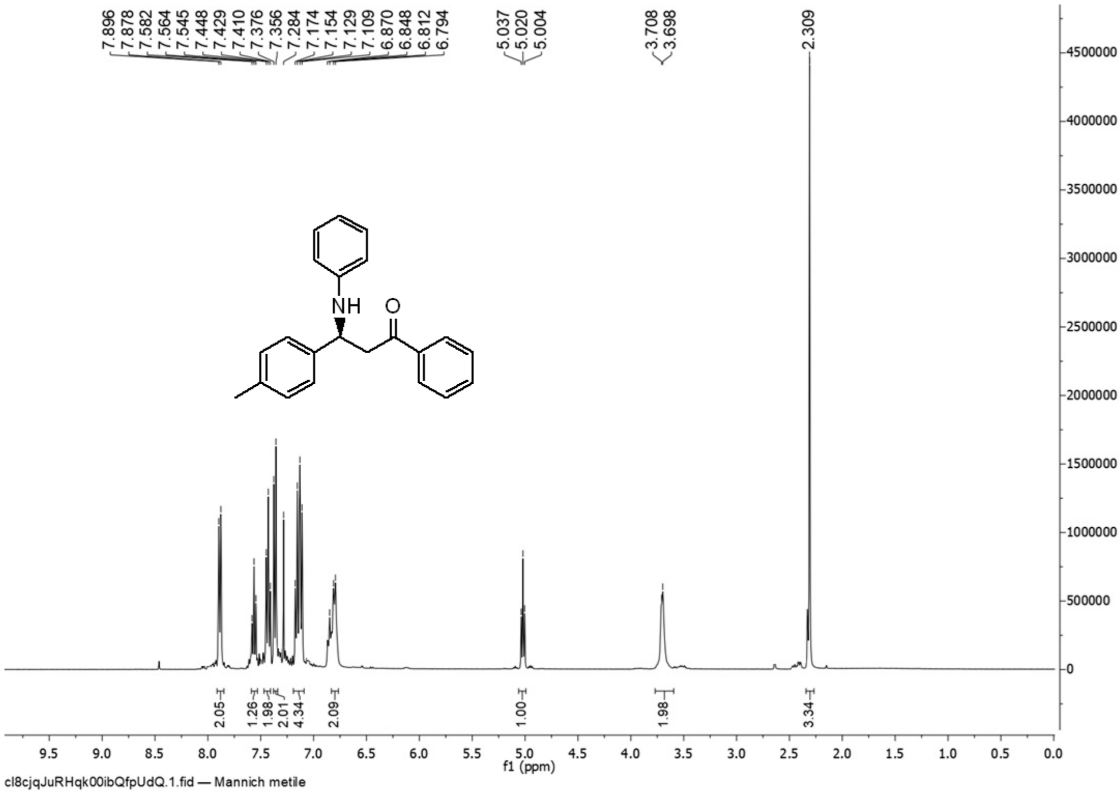

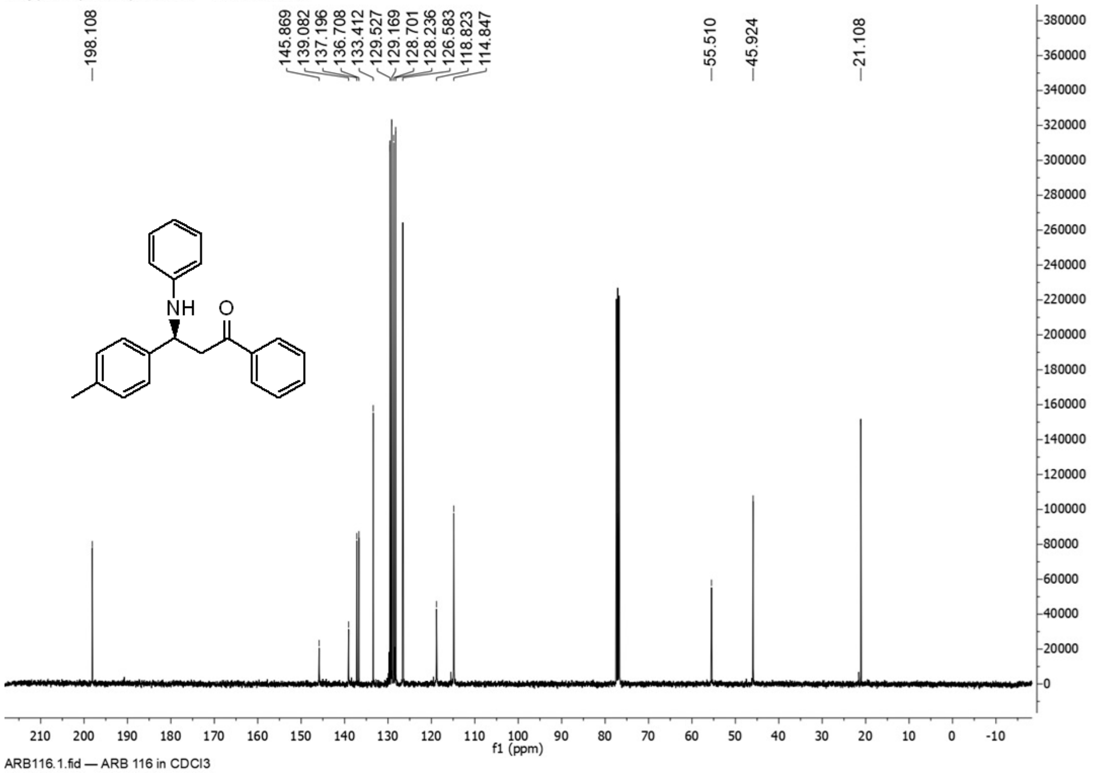

(+)-1-Phenyl-3-(phenylamino)-3-(*p*-tolyl)propan-1-one (11k)

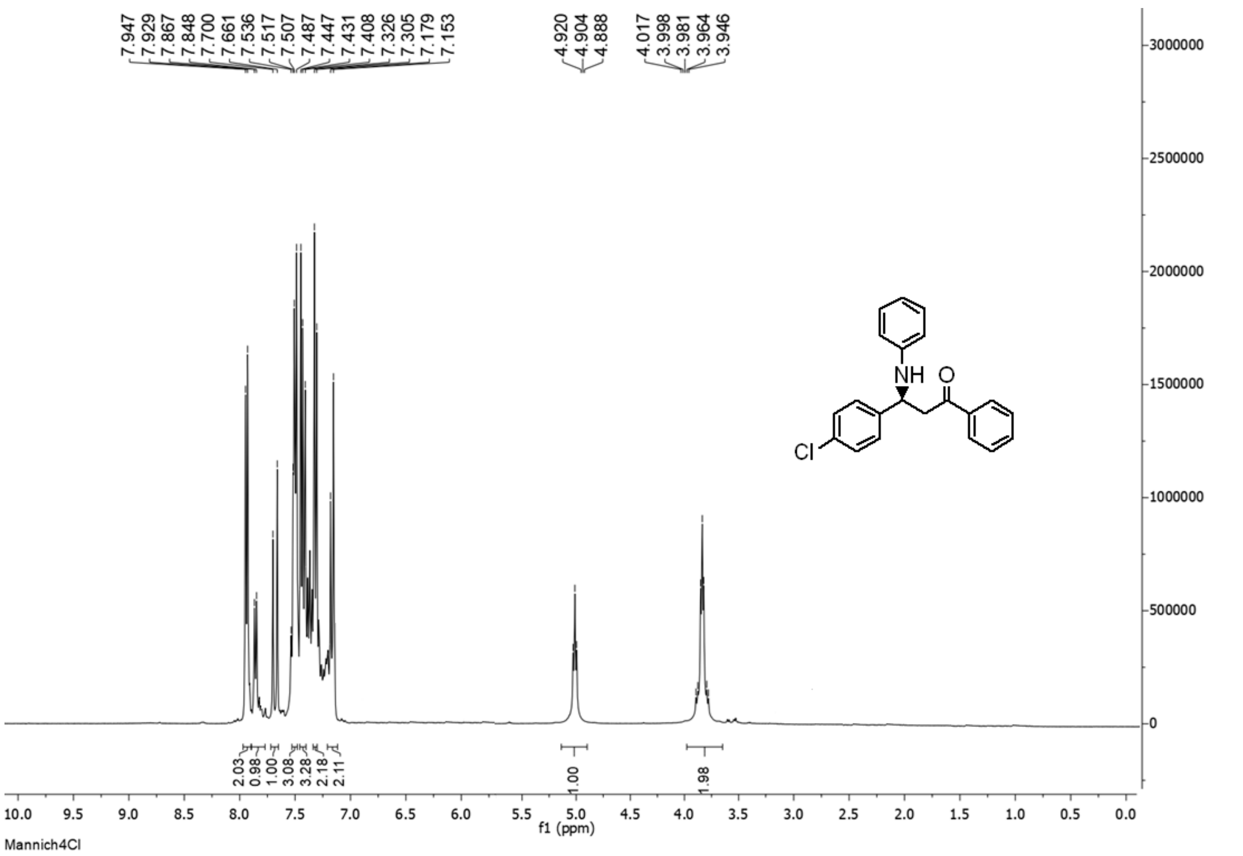

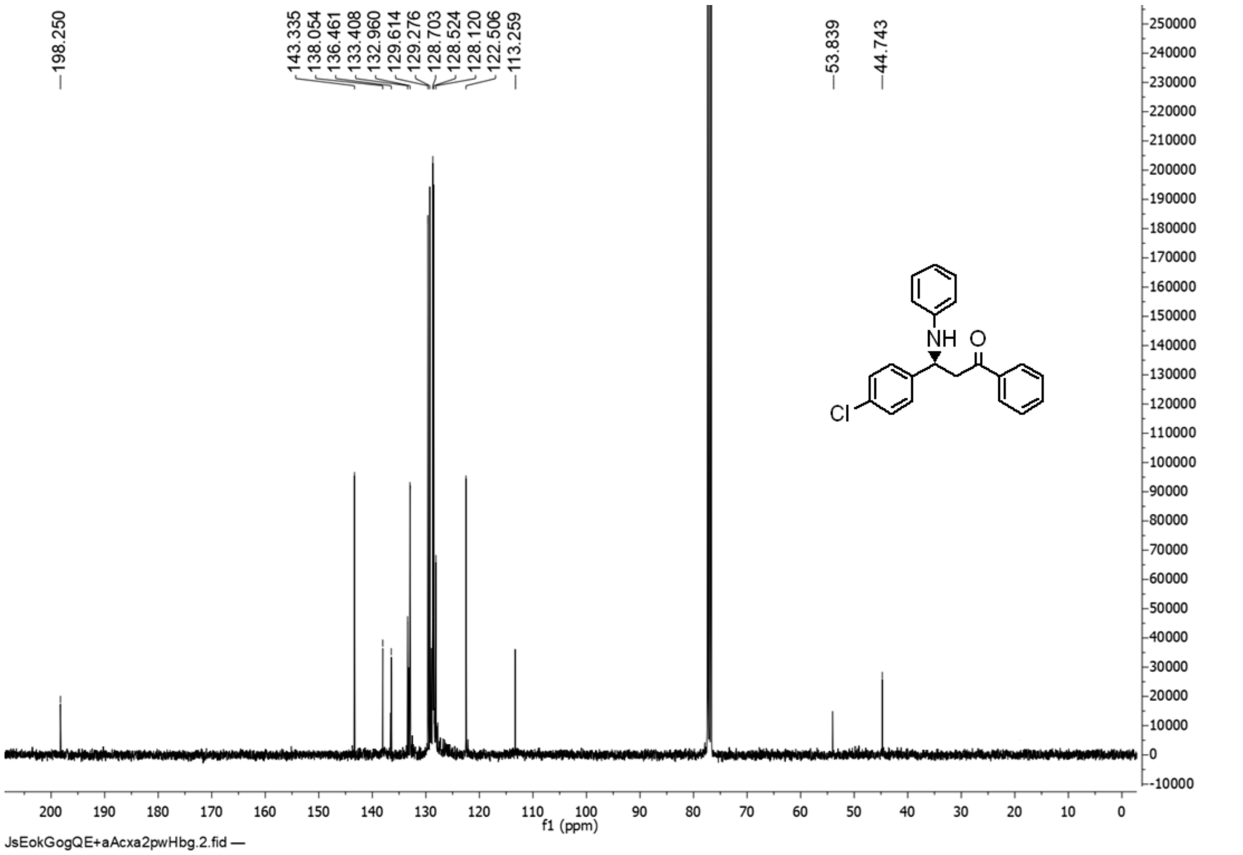

(+)-3-(4-Chlorophenyl)-1-phenyl-3-(phenylamino)propan-1-one (11l)

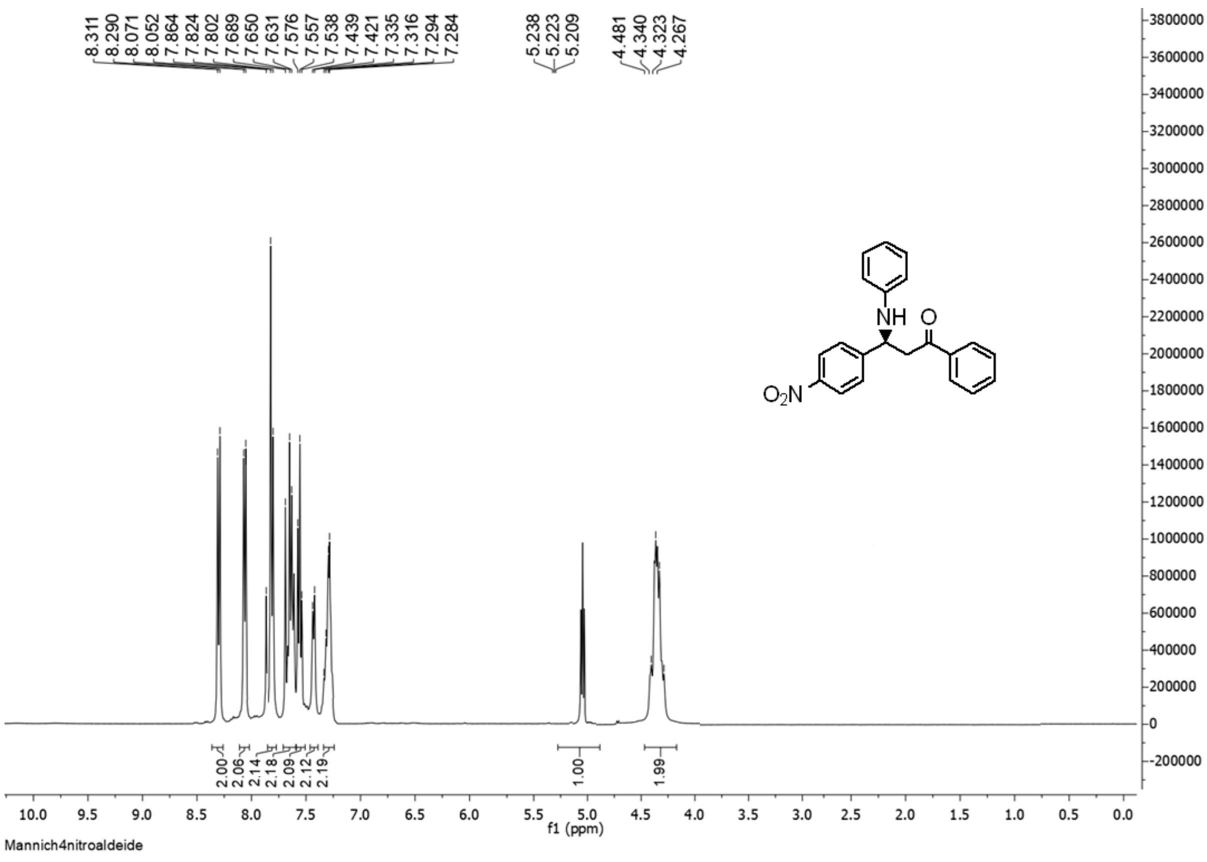

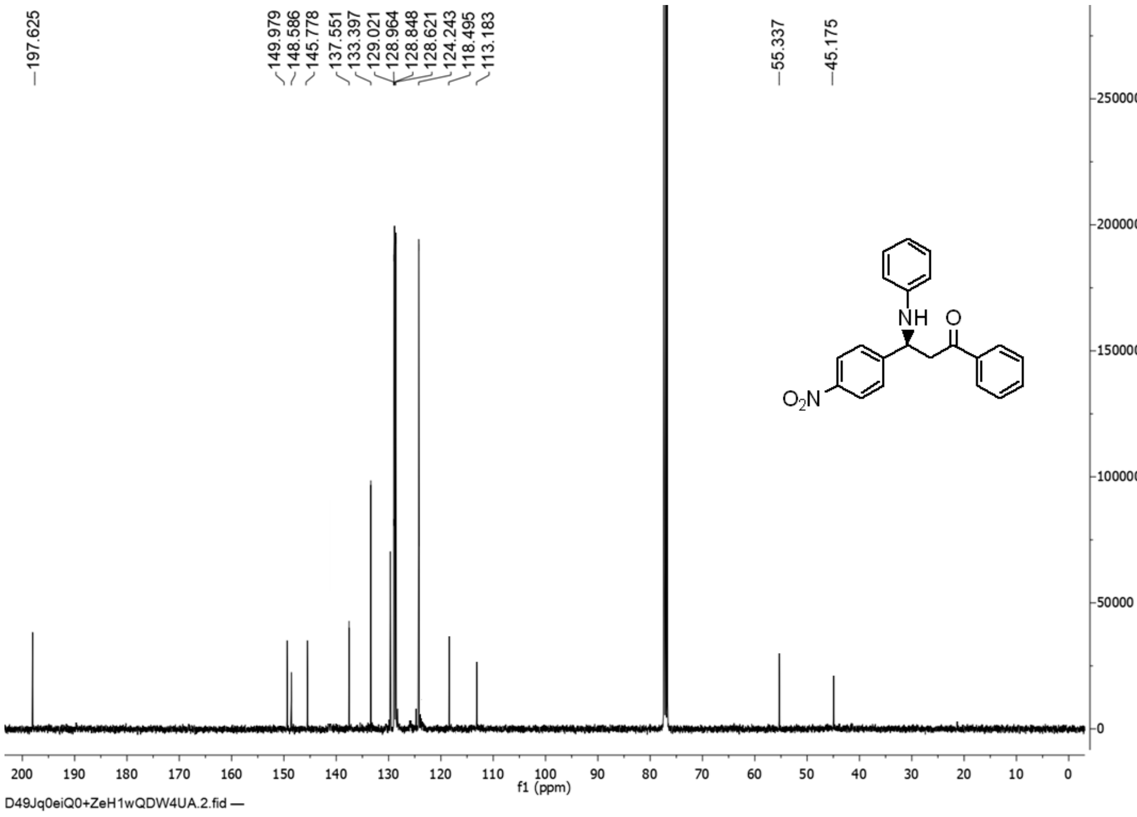

(+)-3-(4-Nitrophenyl)-1-phenyl-3-(phenylamino)propan-1-one (11m)

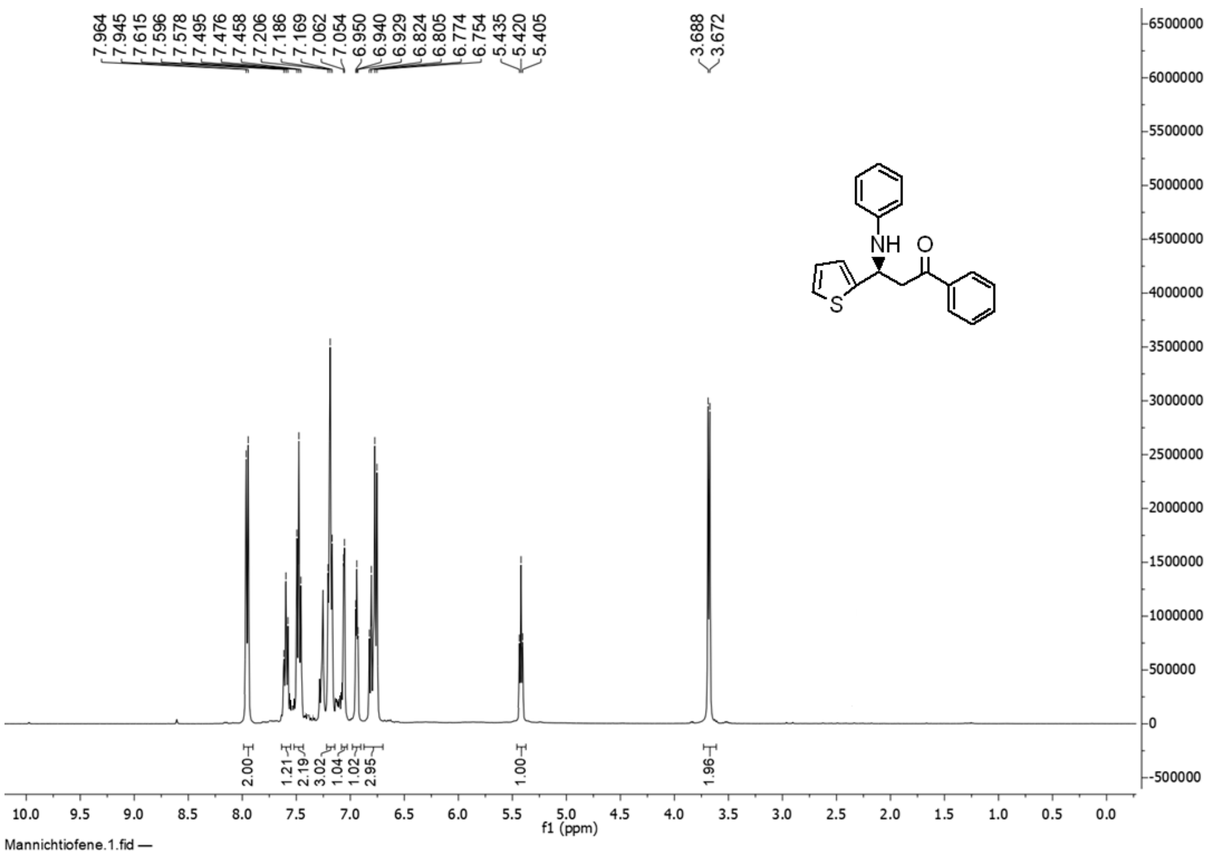

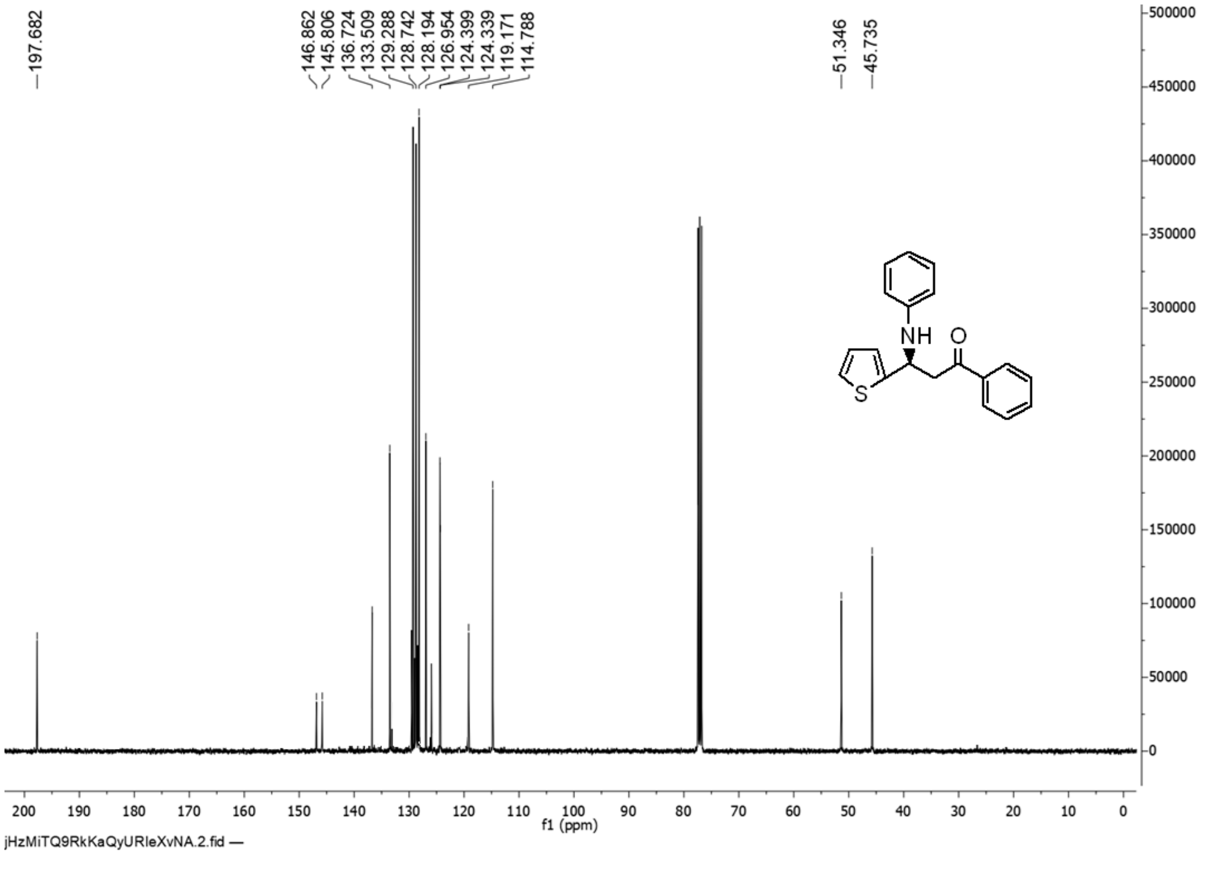

(+)-1-phenyl-3-(phenylamino)-3-(thiophen-2-yl)propan-1-one (11n)

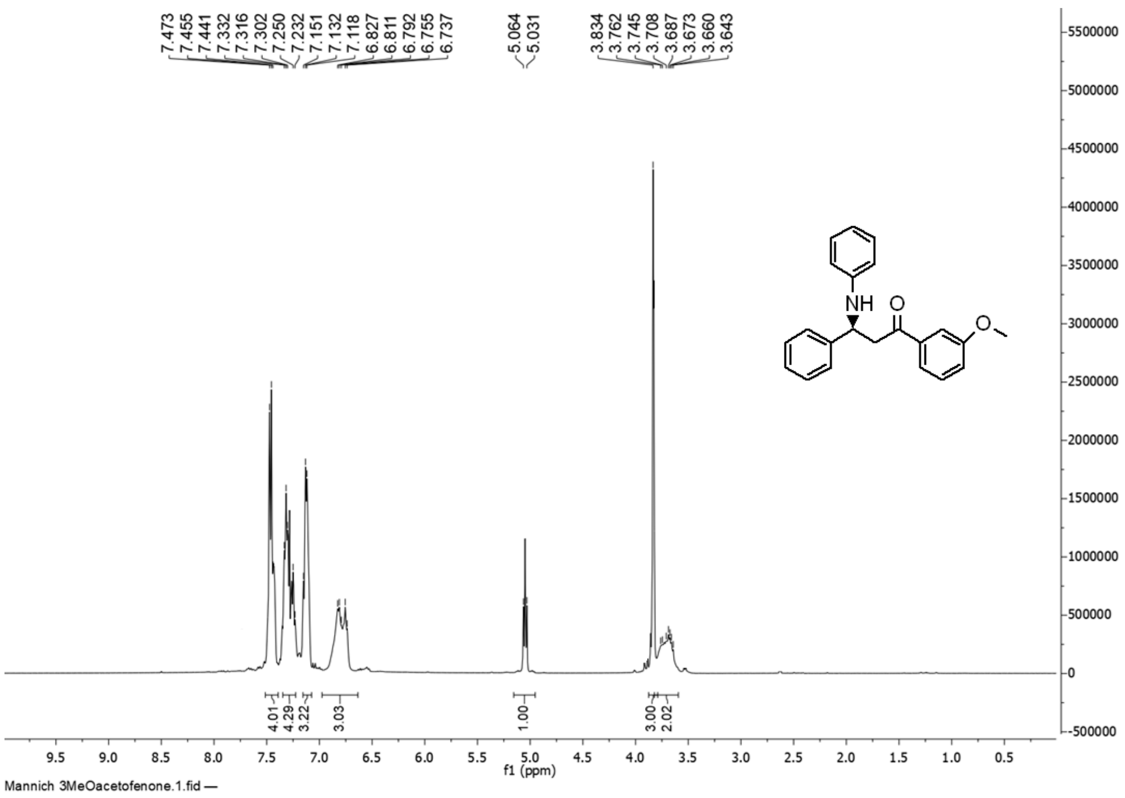

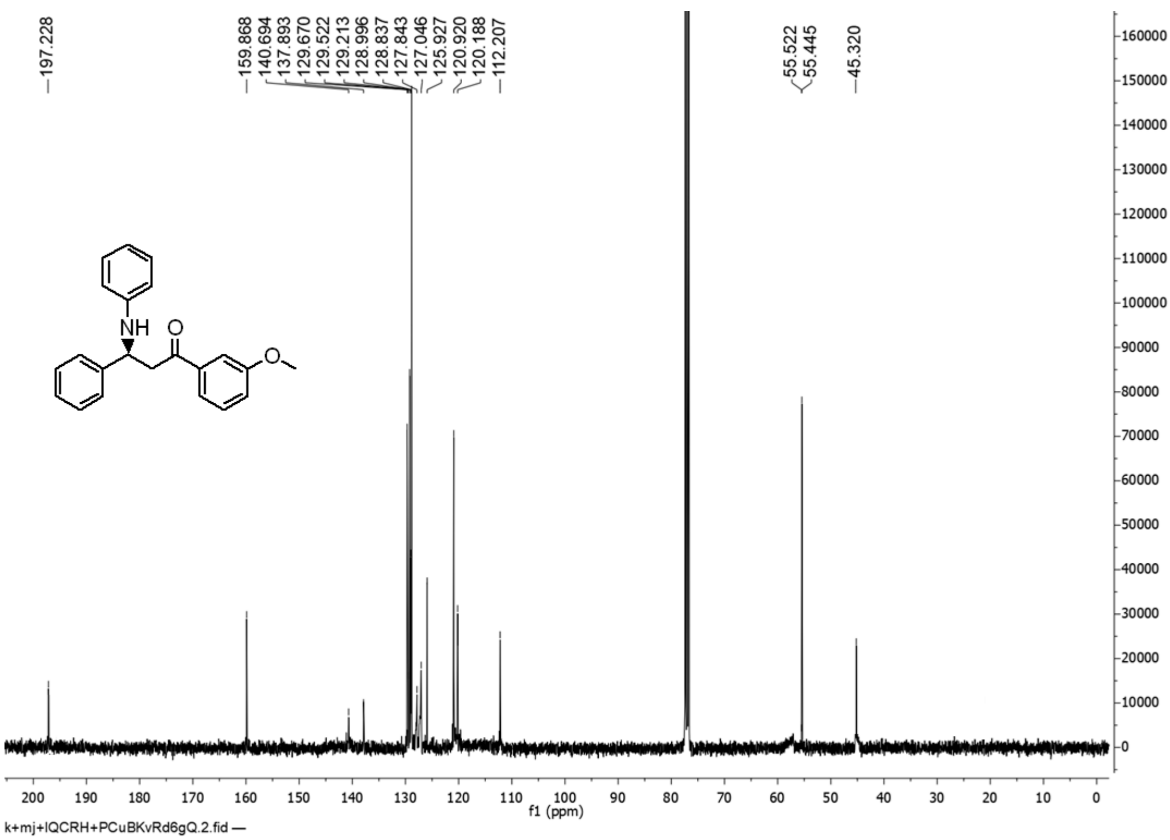

(+)-1-(3-Methoxyphenyl)-3-phenyl-3-(phenylamino)propan-1-one (11o)

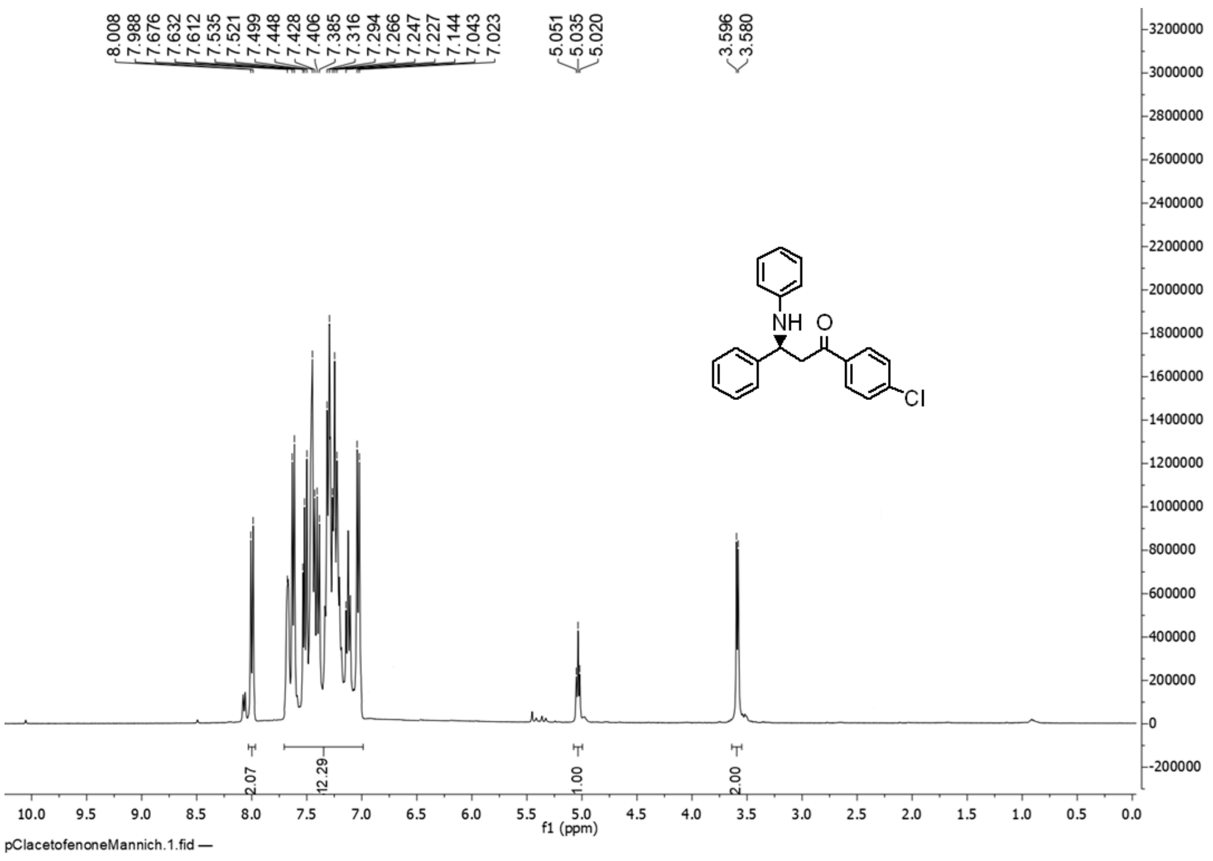

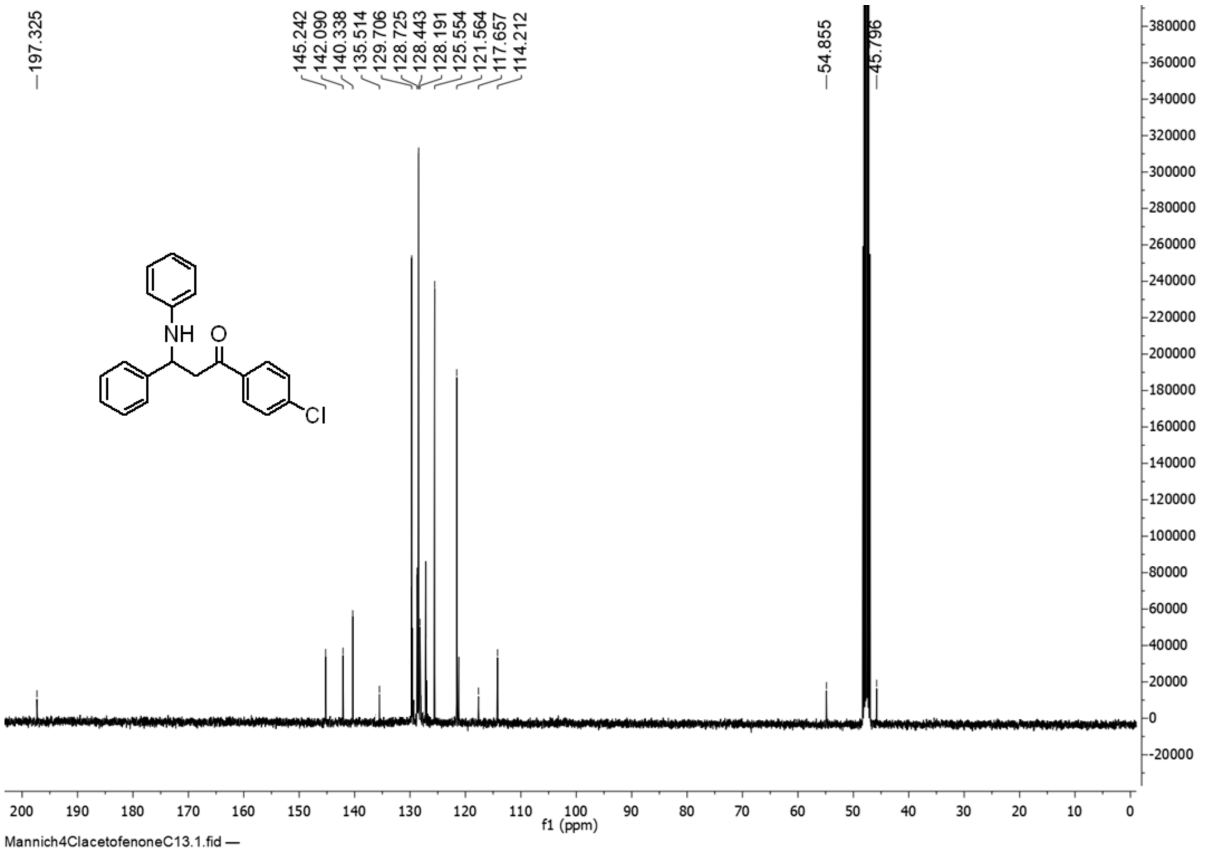

(+)-1-(4-Chlorophenyl)-3-phenyl-3-(phenylamino)propan-1-one (11q)

7. Chiral analyses of compounds 11.

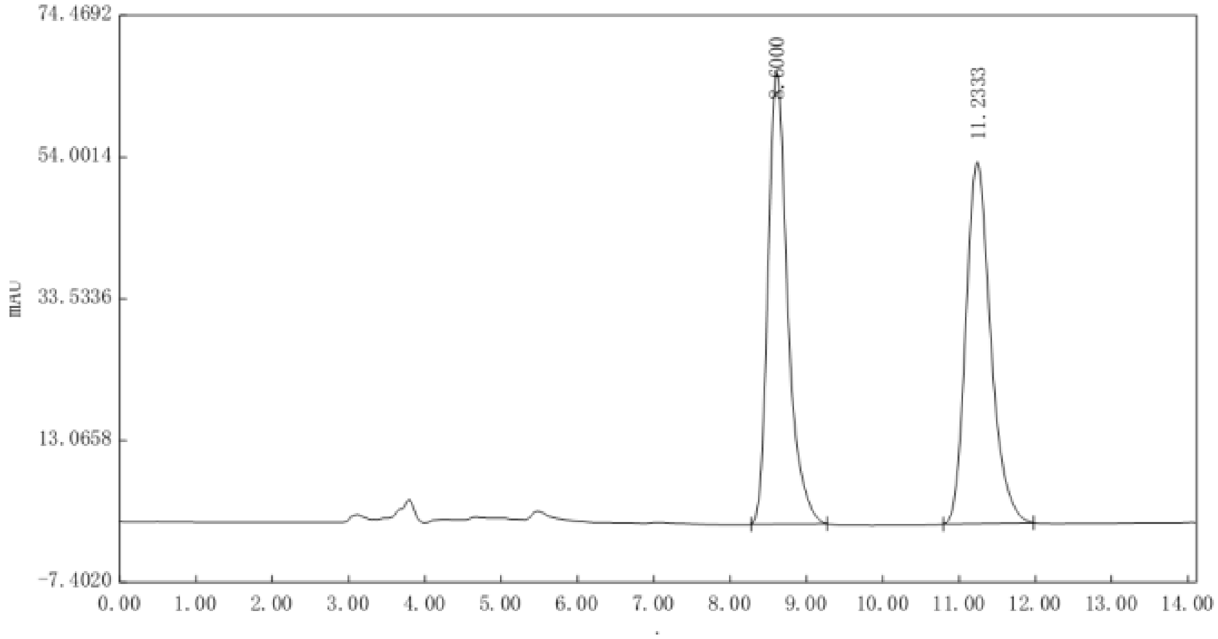

Peak	RT [min]	Height [mV]	Height %	Area[mV*s]	Area%
1	8.6000	68.0210	55.5556	1213.0498	49.6744
2	11.2333	54.4168	44.4444	1228.9500	50.3256

(rac) 1,3-Diphenyl-3-(phenylamino)propan-1-one (11a).

Daicel Chiralpak AD-H *n*-hexane/*i*-propanol 4:1; flow rate = 1.0mL/min, λ = 254 nm

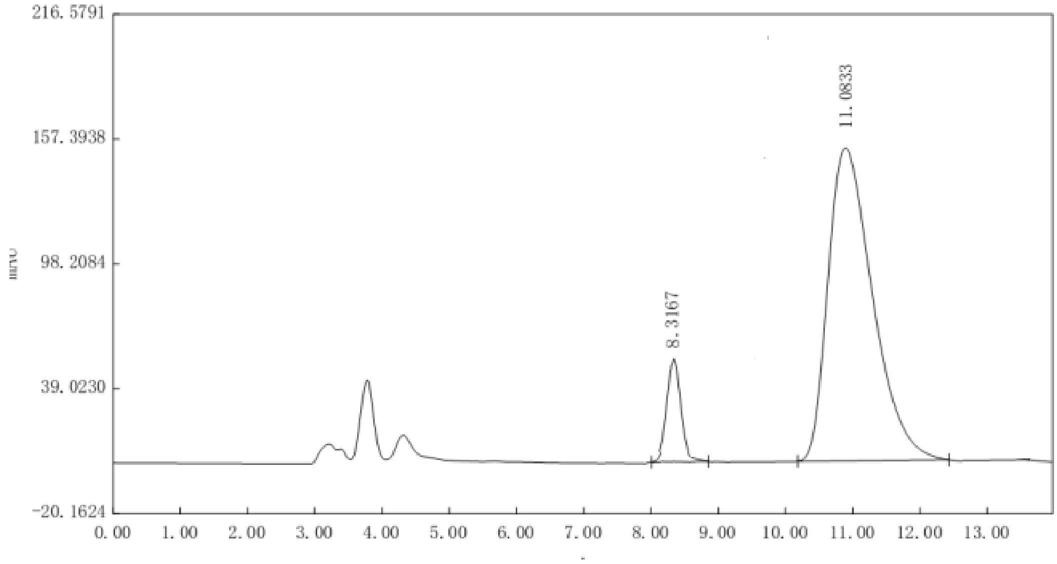

Peak	RT [min]	Height [mV]	Height %	Area[mV*s]	Area%
1	8.3167	67.4168	29.7872	417.0498	25.3372
2	11.0833	158.9167	70.2128	1228.9500	74.6628

(+) 1,3-Diphenyl-3-(phenylamino)propan-1-one (11a).

Daicel Chiralpak AD-H *n*-hexane/*i*-propanol 4:1; flow rate = 1.0mL/min, λ = 254 nm

Reaction with commercial Phencyphos (Table 1; entry 4).

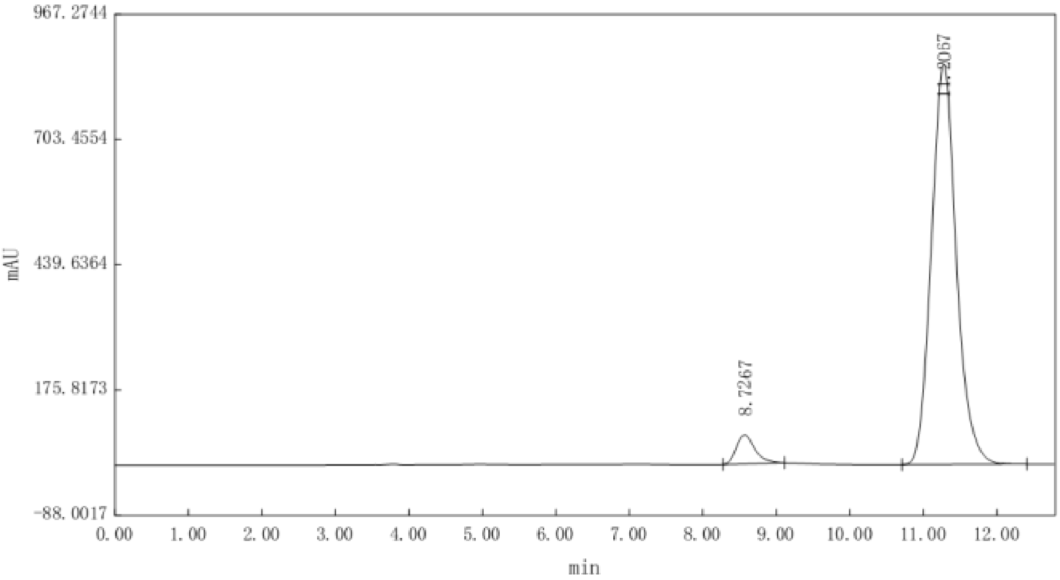

Peak	RT [min]	Height [mV]	Height %	Area[mV*s]	Area%
1	8.7267	63.1522	6.7014	1029.3335	4.8330
2	11.2067	879.2208	93.2986	20268.5504	95.1670

(+) 1,3-Diphenyl-3-(phenylamino)propan-1-one (11a).

Daicel Chiralpak AD-H *n*-hexane/*i*-propanol 4:1; flow rate = 1.0mL/min, λ = 254 nm

Reaction with catalyst 7 (Table 1; entry 6).

(rac)-3-((2-Nitrophenyl)amino)-1,3-diphenylpropan-1-one (11b).

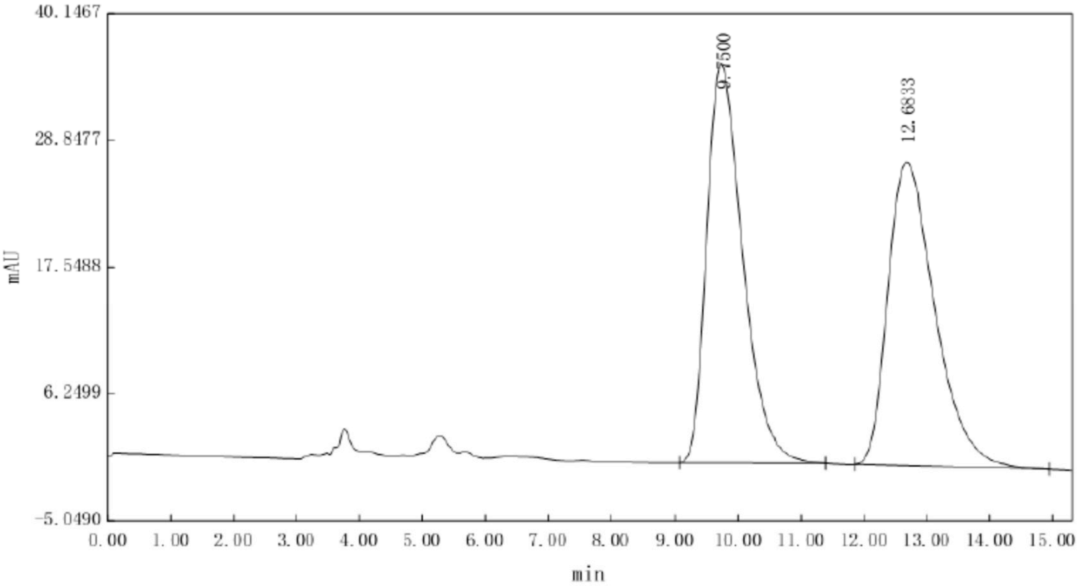

Peak	RT [min]	Height [mV]	Height %	Area[mV*s]	Area%
1	9.7500	36.7690	56.6673	1471.6510	50.2333
2	12.6833	28.1168	43.3327	1457.9825	49.7667

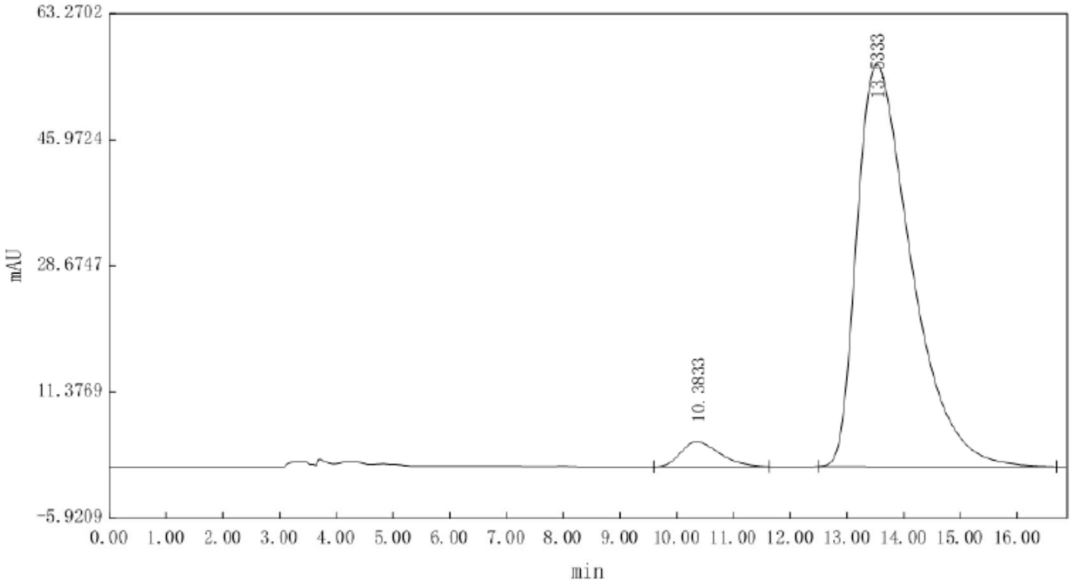

Peak	RT [min]	Height [mV]	Height %	Area[mV*s]	Area%
1	10.3833	3.5858	5.8759	169.6351	4.2551
2	13.5333	57.4399	94.1241	3816.9671	95.7449

(+)-3-((2-Nitrophenyl)amino)-1,3-diphenylpropan-1-one (11b).

Daicel Chiralpak AD-H *n*-hexane/*i*-propanol 1:1; flow rate = 1.5mL/min, λ = 254 nm

(rac)-3-((3-Fluorophenyl)amino)-1,3-diphenylpropan-1-one (11c).

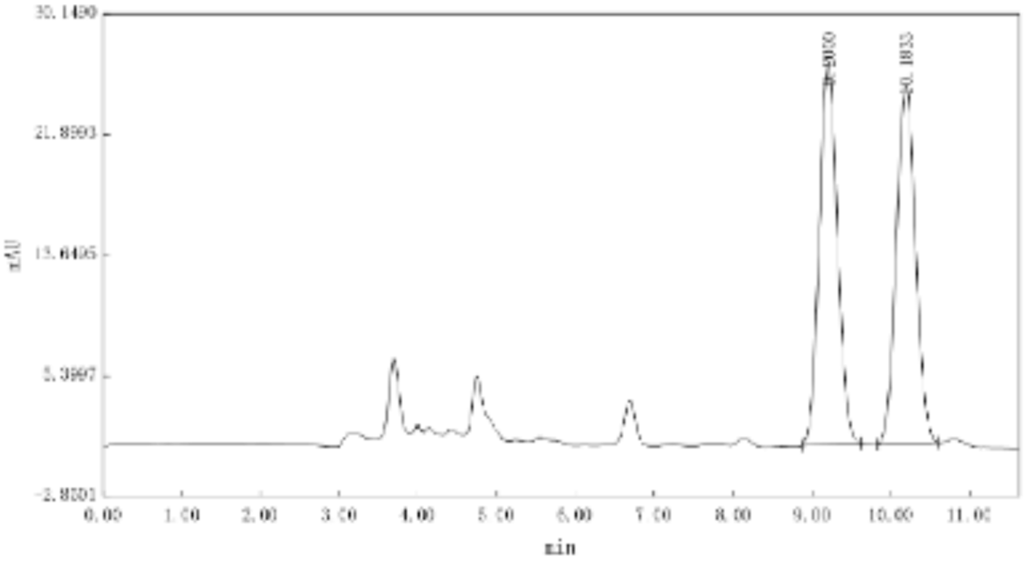

Peak	RT [min]	Height [mV]	Height %	Area[mV*s]	Area%
1	9.2000	27.2108	51.6309	432.3443	49.8400
2	10.1833	25.4918	48.3691	435.1195	50.1600

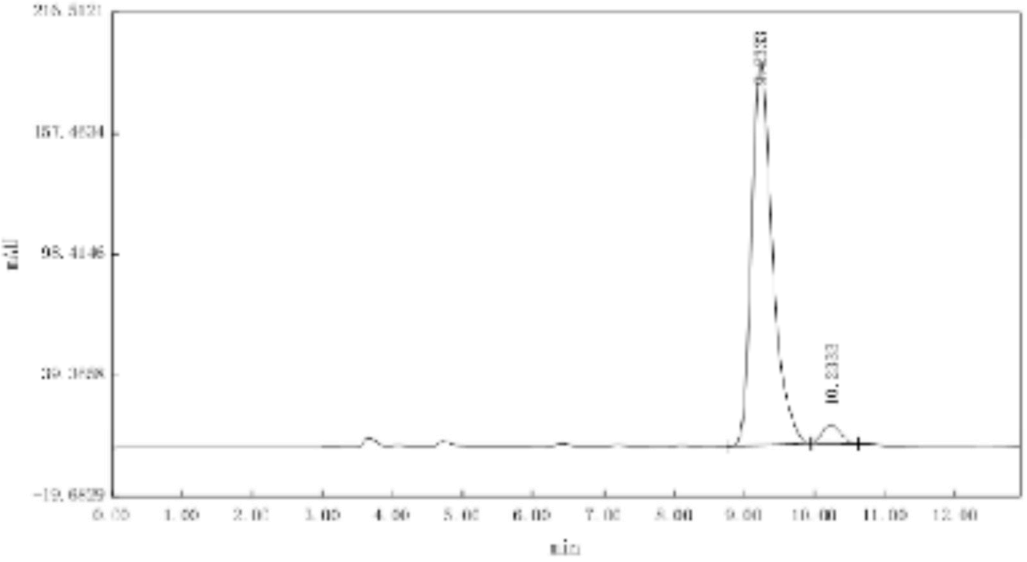

Peak	RT [min]	Height [mV]	Height %	Area[mV*s]	Area%
1	9.2333	195.2795	95.2872	4031.0603	95.8635
2	10.2333	9.6584	4.7128	173.9410	4.1365

(+)-3-((3-Fluorophenyl)amino)-1,3-diphenylpropan-1-one (11c).

Daicel Chiralpak AD-H *n*-hexane/*i*-propanol 4:1; flow rate = 1.0mL/min, λ = 254 nm

(rac)-1,3-Diphenyl-3-(p-tolylamino)propan-1-one (11d).

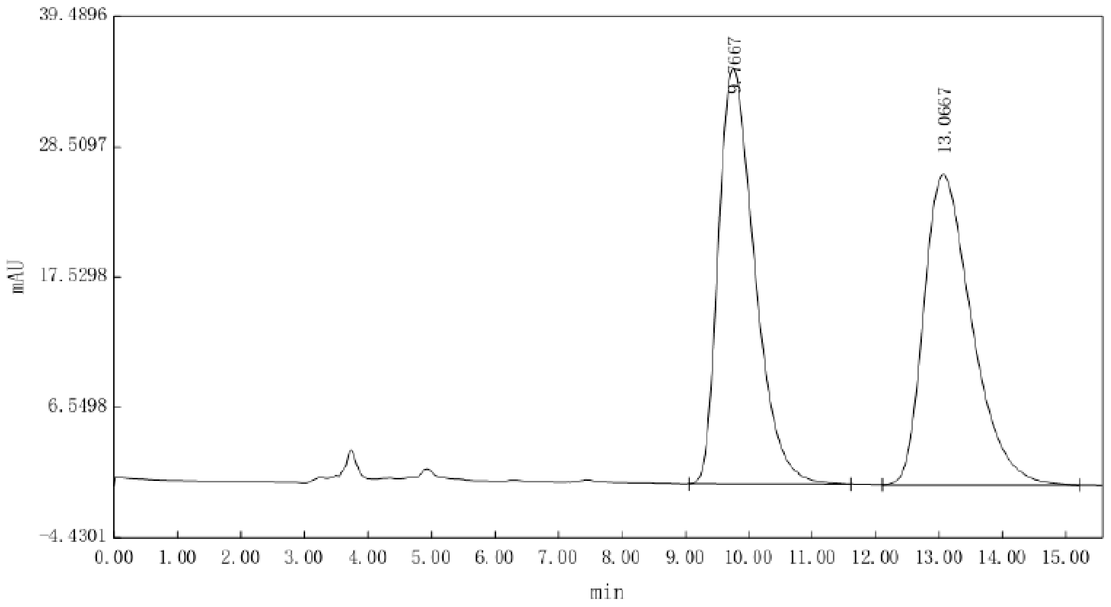

Peak	RT [min]	Height [mV]	Height %	Area[mV*s]	Area%
1	9.7667	36.3255	57.1536	1449.1967	50.2473
2	13.0667	27.2322	42.8464	1434.9345	49.7527

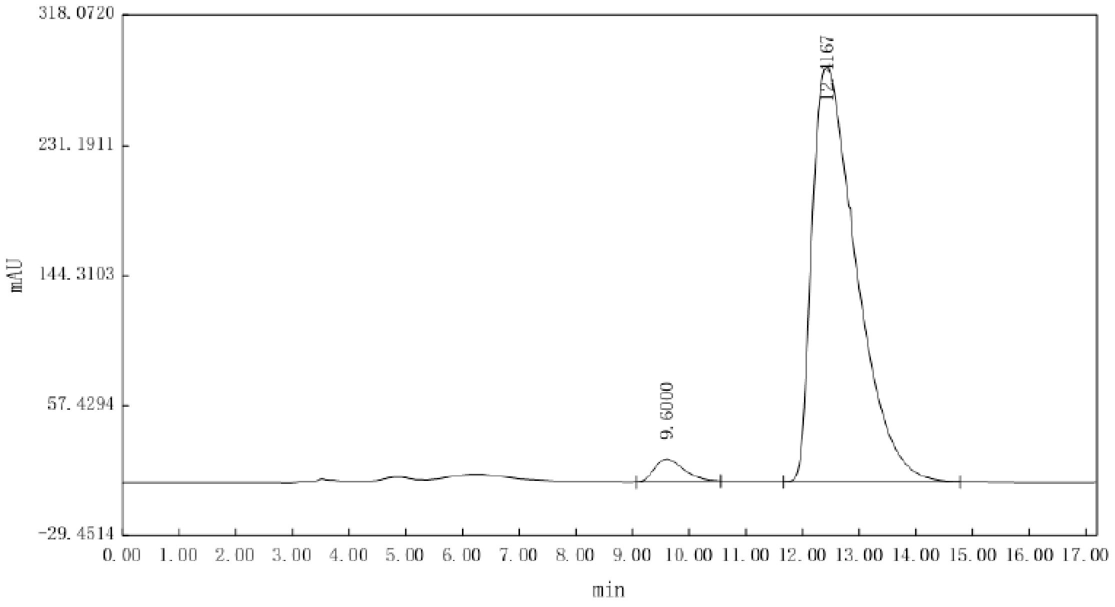

Peak	RT [min]	Height [mV]	Height %	Area[mV*s]	Area%
1	9.6000	15.7857	5.1901	574.1440	3.5308
2	12.4167	288.3678	94.8099	15686.9186	96.4692

(+)-1,3-Diphenyl-3-(p-tolylamino)propan-1-one (11d).

Daicel Chiralpak AD-H *n*-hexane/*i*-propanol 4:1; flow rate = 1.0mL/min, λ = 254 nm

(rac)-3-((4-Nitrophenyl)amino)-1,3-diphenylpropan-1-one (11e).

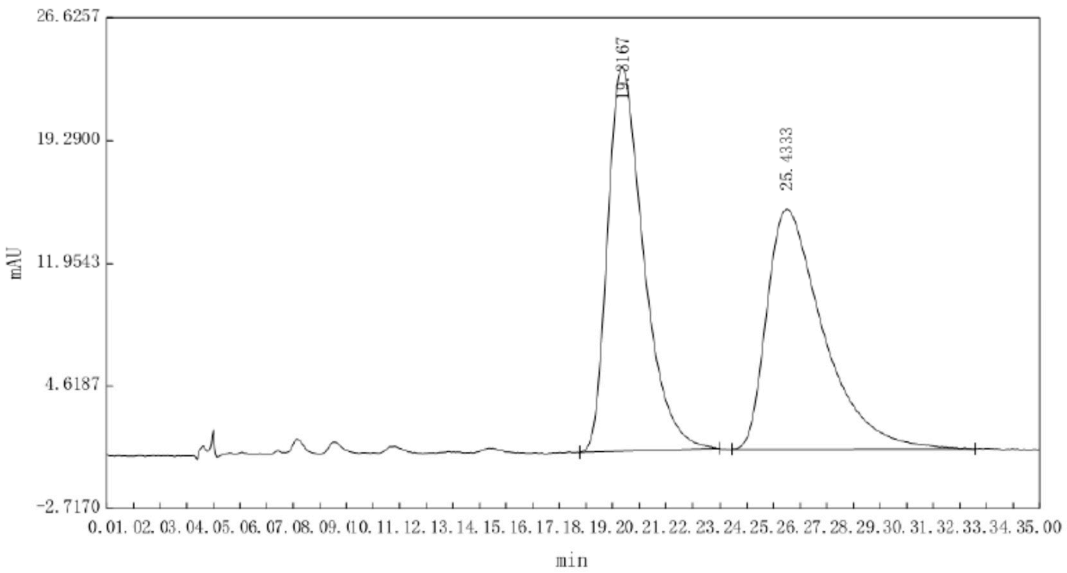

Peak	RT [min]	Height [mV]	Height %	Area[mV*s]	Area%
1	19.3167	23.9301	61.5088	2238.5092	50.3429
2	25.4333	14.9751	38.4912	2208.0130	49.6571

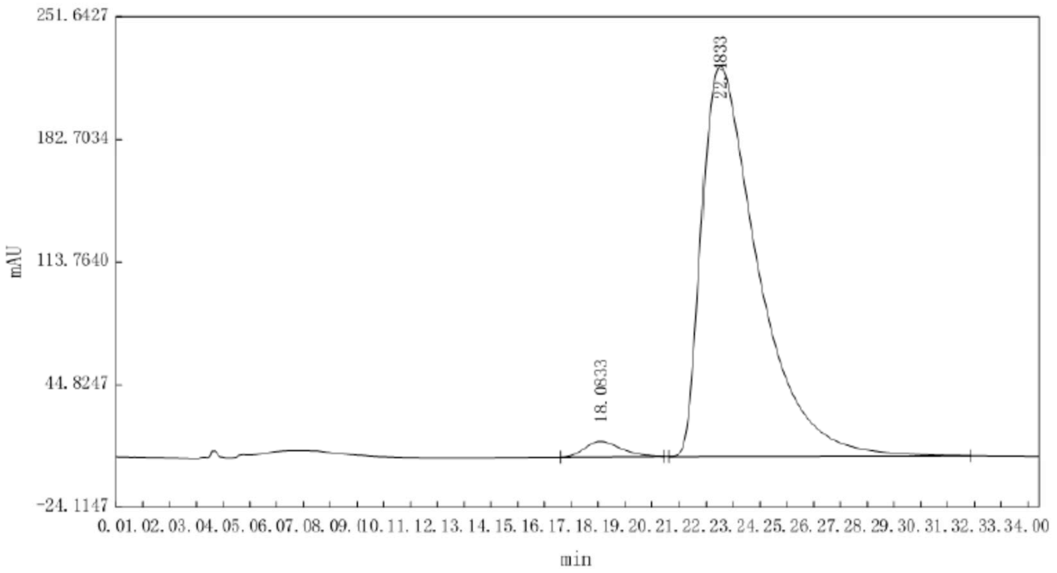

Peak	RT [min]	Height [mV]	Height %	Area[mV*s]	Area%
1	18.0833	9.0194	3.7963	845.6914	2.4995
2	22.4833	228.5652	96.2037	32988.1487	97.5005

(+)-3-((4-Nitrophenyl)amino)-1,3-diphenylpropan-1-one (11e).

Daicel Chiralpak AD-H *n*-hexane/*i*-propanol 4:1; flow rate = 1.0mL/min, λ = 254 nm

(rac)-3-((4-Bromophenyl)amino)-1,3-diphenylpropan-1-one (11f)

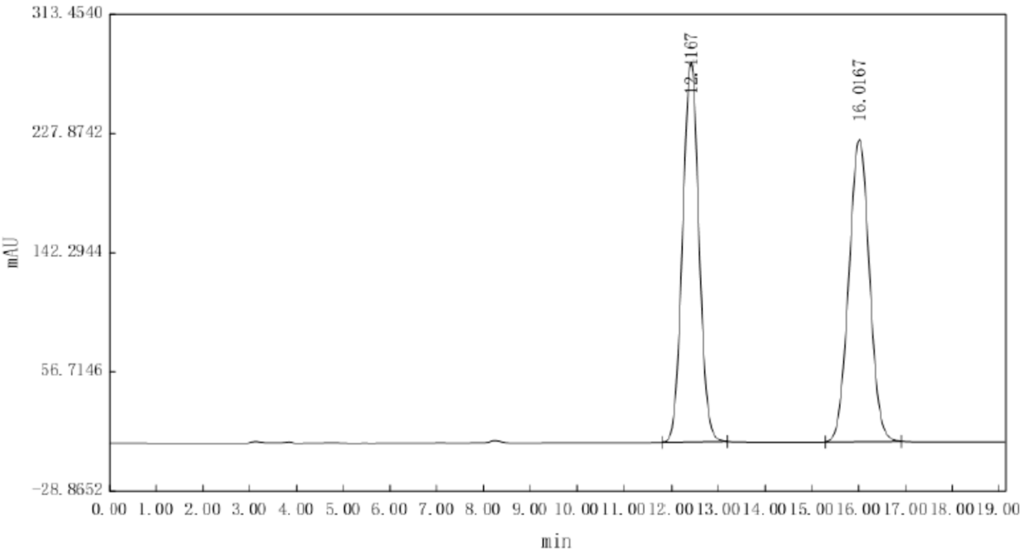

Peak	RT [min]	Height [mV]	Height %	Area[mV*s]	Area%
1	12.4167	284.1740	55.6770	6947.2159	50.0935
2	16.0167	226.2239	44.3230	6921.2712	49.9065

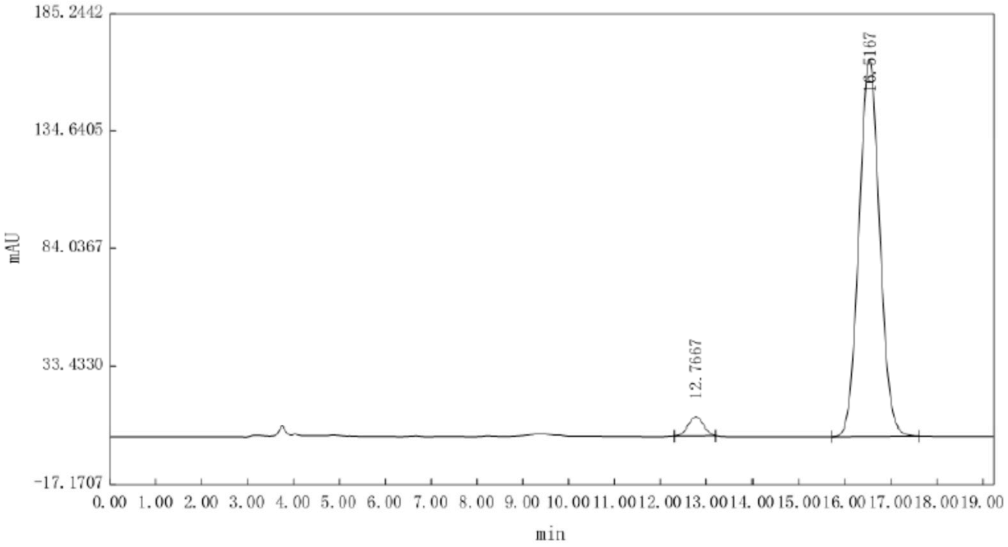

Peak	RT [min]	Height [mV]	Height %	Area[mV*s]	Area%
1	12.7667	8.0776	4.5817	187.7192	3.4488
2	16.5167	168.2261	95.4183	5255.3039	96.5512

(+)-3-((4-Bromophenyl)amino)-1,3-diphenylpropan-1-one (11f)

Daicel Chiralpak AD-H *n*-hexane/*i*-propanol 4:1; flow rate = 1.0mL/min, λ = 254 nm

(rac) 3-((2,6-Dimethylphenyl)amino)-1,3-diphenylpropan-1-one (11g)

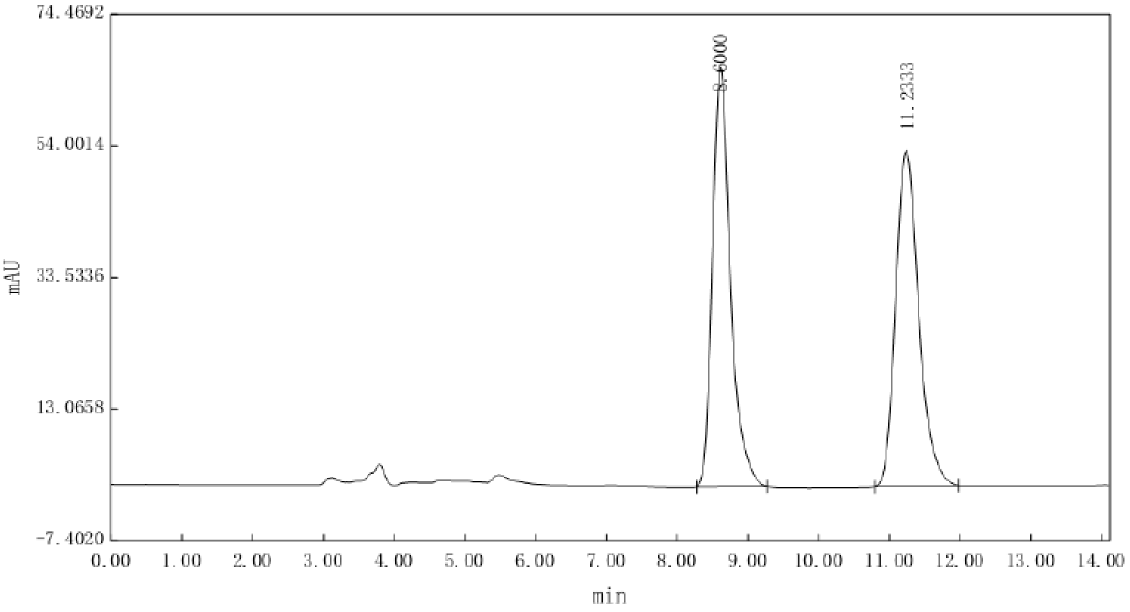

Peak	RT [min]	Height [mV]	Height %	Area[mV*s]	Area%
1	8.6000	68.0210	55.5556	1213.0498	49.6744
2	11.2333	54.4168	44.4444	1228.9500	50.3256

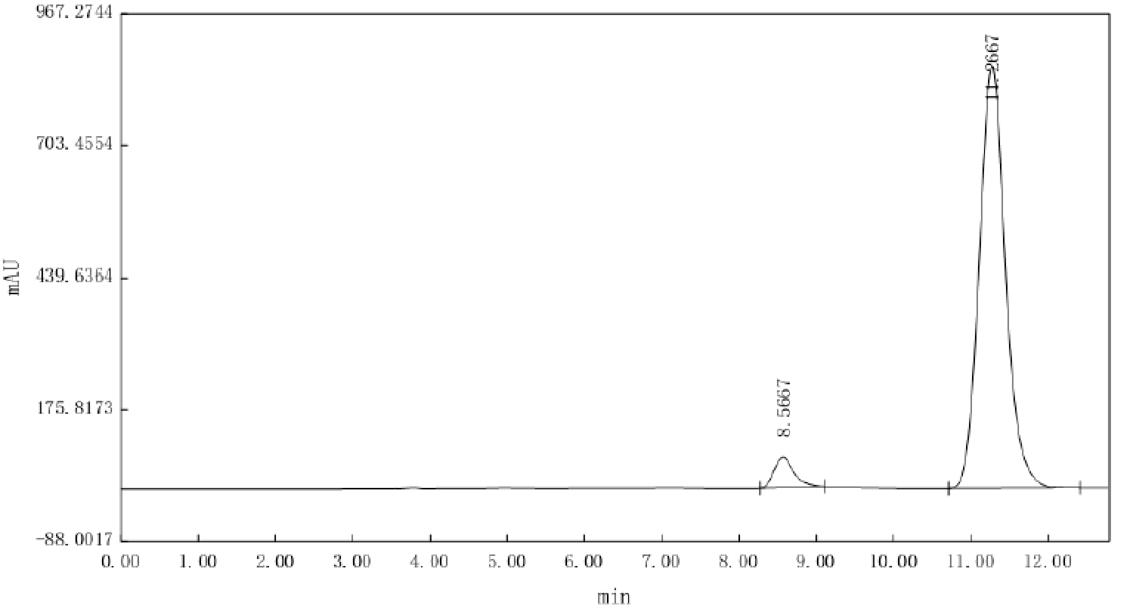

Peak	RT [min]	Height [mV]	Height %	Area[mV*s]	Area%
1	8.5667	64.0322	6.8000	1126.8375	5.1606
2	11.2667	877.6205	93.2000	20708.5671	94.8394

(+) 3-((2,6-Dimethylphenyl)amino)-1,3-diphenylpropan-1-one (11g)

Daicel Chiralpak AD-H *n*-hexane/*i*-propanol 4:1; flow rate = 1.0mL/min, λ = 254 nm

(rac)-3-(Benzylamino)-1,3-diphenylpropan-1-one (11h)

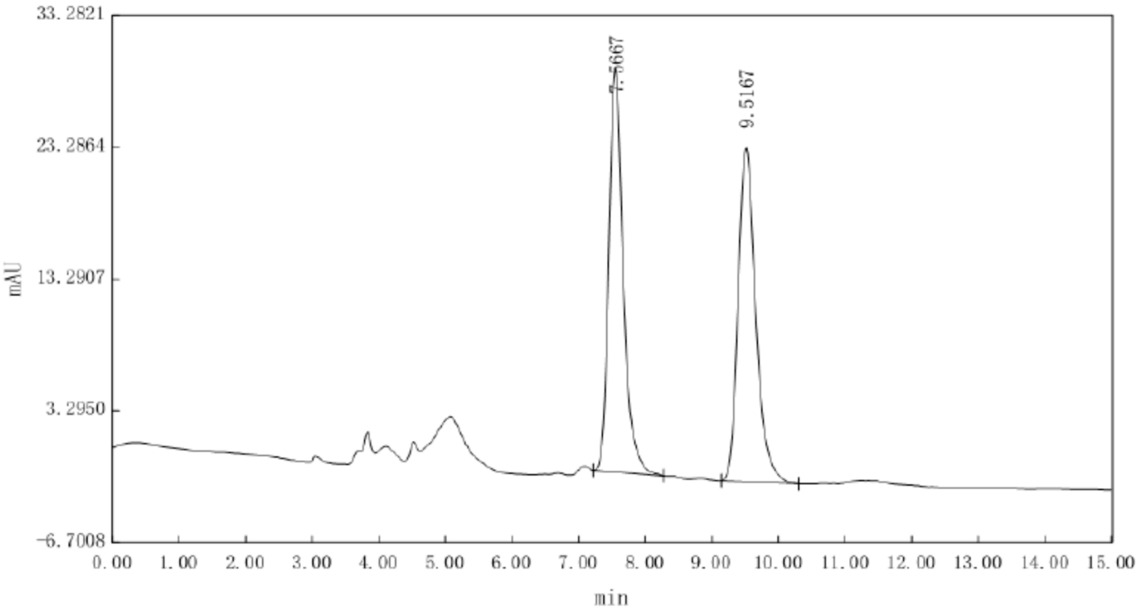

Peak	RT [min]	Height [mV]	Height %	Area[mV*s]	Area%
1	7.5667	30.9158	53.9595	478.9362	49.7702
2	9.5167	26.3787	46.0405	483.3588	50.2298

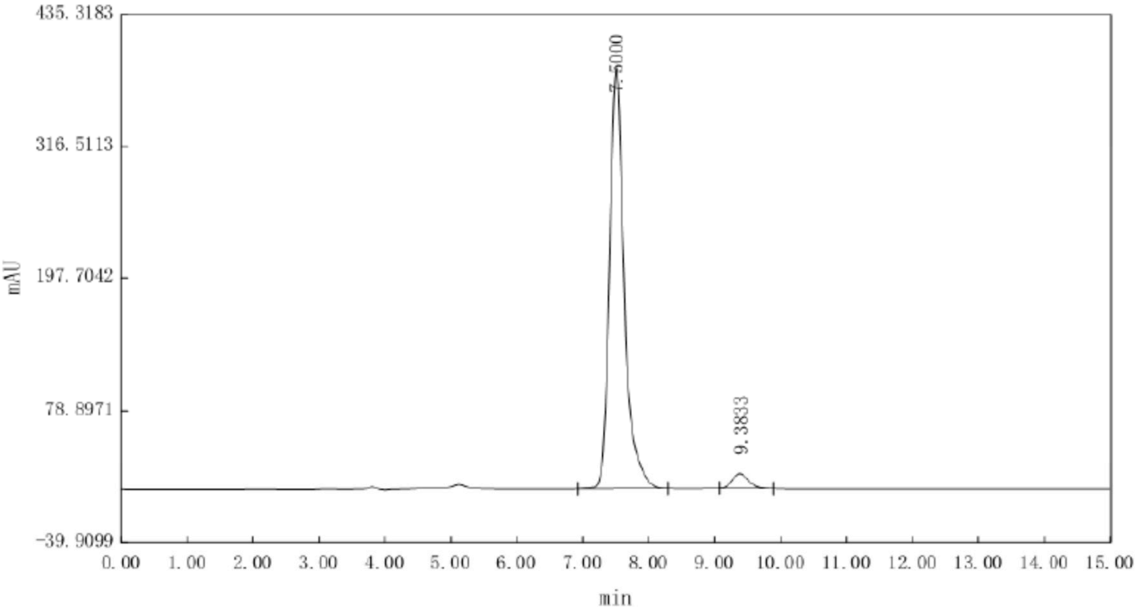

Peak	RT [min]	Height [mV]	Height %	Area[mV*s]	Area%
1	7.5000	394.8219	96.7024	6173.8823	96.3013
2	9.3833	13.4635	3.2976	237.1220	3.6987

(+)-3-(Benzylamino)-1,3-diphenylpropan-1-one (11h)

Daicel Chiralpak AD-H *n*-hexane/*i*-propanol 4:1; flow rate = 1.0mL/min, λ = 254 nm

(rac)-3-(3-Methoxyphenyl)-1-phenyl-3-(phenylamino)propan-1-one (11i).

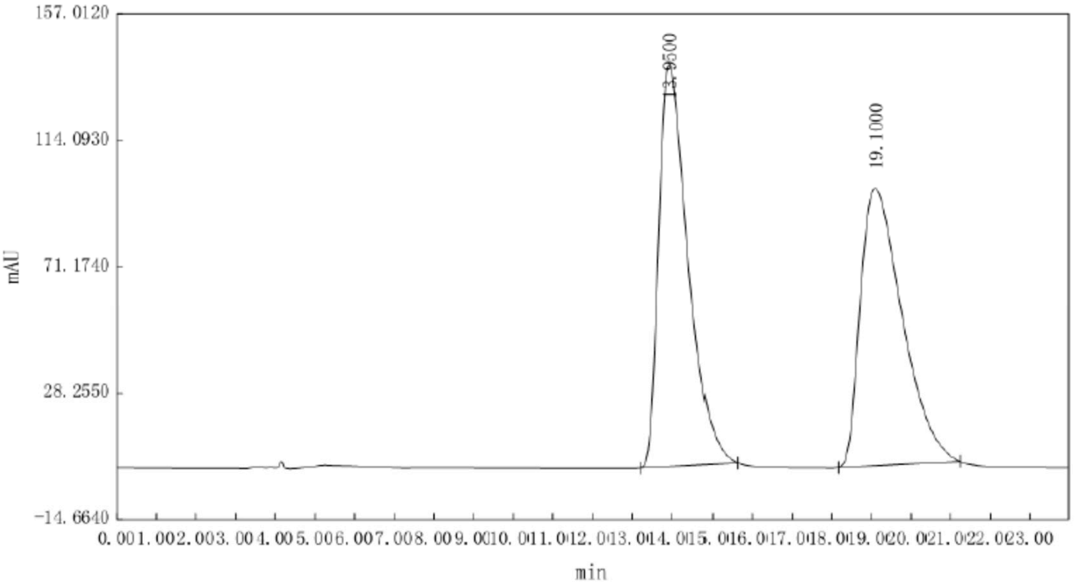

Peak	RT [min]	Height [mV]	Height %	Area[mV*s]	Area%
1	13.9500	142.1550	59.1584	7238.2040	50.1658
2	19.1000	98.1404	40.8416	7190.3509	49.8342

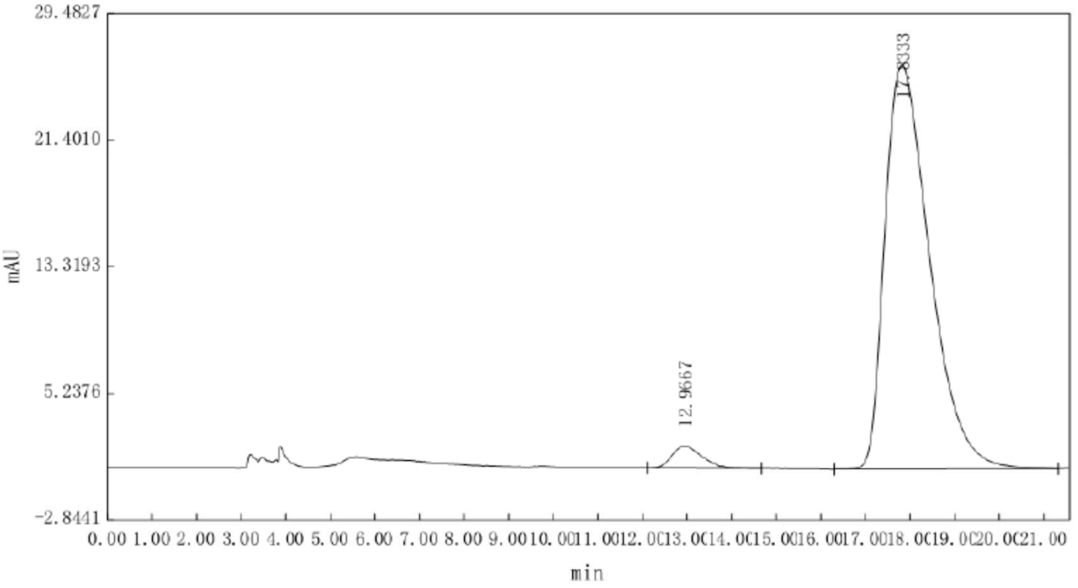

Peak	RT [min]	Height [mV]	Height %	Area[mV*s]	Area%
1	12.9667	1.4591	5.1480	68.8697	3.5291
2	17.8333	26.8841	94.8520	1882.5925	96.4709

(+)-3-(3-Methoxyphenyl)-1-phenyl-3-(phenylamino)propan-1-one (11i).

Daicel Chiralpak AD-H *n*-hexane/*i*-propanol 4:1; flow rate = 1.0mL/min, λ = 254 nm

(rac)-1-Phenyl-3-(phenylamino)-3-(*p*-tolyl)propan-1-one (11k)

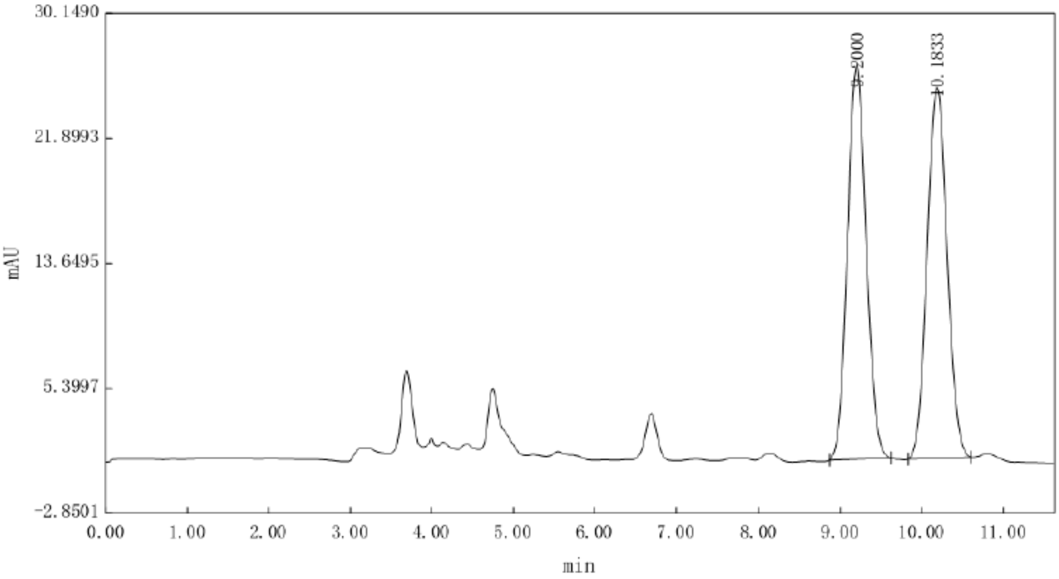

Peak	RT [min]	Height [mV]	Height %	Area[mV*s]	Area%
1	9.2000	27.2108	51.6309	432.3443	49.8400
2	10.1833	25.4918	48.3691	435.1195	50.1600

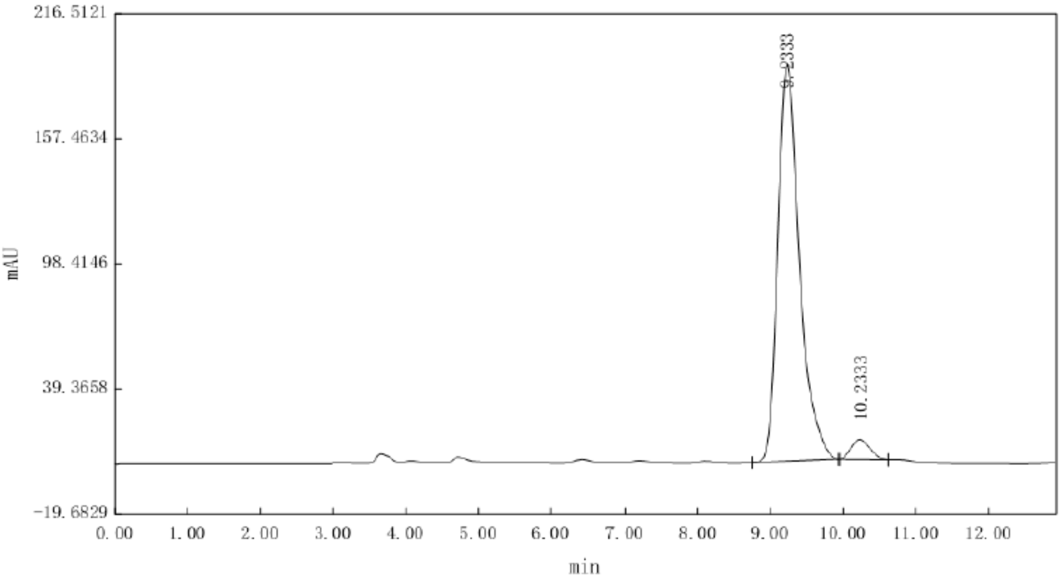

Peak	RT [min]	Height [mV]	Height %	Area[mV*s]	Area%
1	9.2333	195.2795	95.2872	4031.0603	95.8635
2	10.2333	9.6584	4.7128	173.9410	4.1365

(+)-1-Phenyl-3-(phenylamino)-3-(*p*-tolyl)propan-1-one (11k)

Daicel Chiralpak AD-H *n*-hexane/*i*-propanol 4:1; flow rate = 1.0mL/min, λ = 254 nm

(rac)-3-(4-Chlorophenyl)-1-phenyl-3-(phenylamino)propan-1-one (11l)

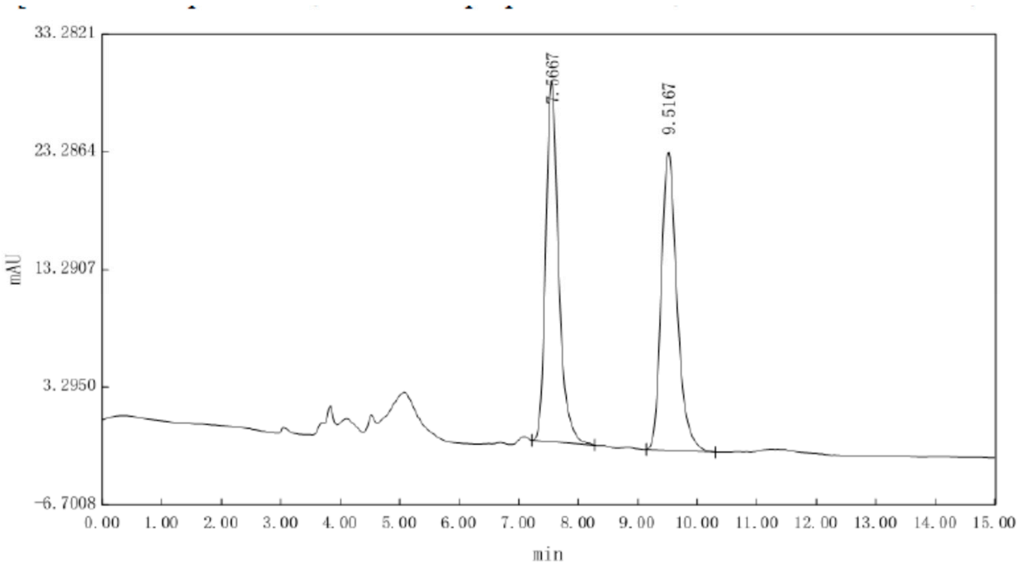

Peak	RT [min]	Height [mV]	Height %	Area[mV*s]	Area%
1	7.5667	30.9158	53.9595	478.9362	49.7702
2	9.5167	26.3787	46.0405	483.3588	50.2298

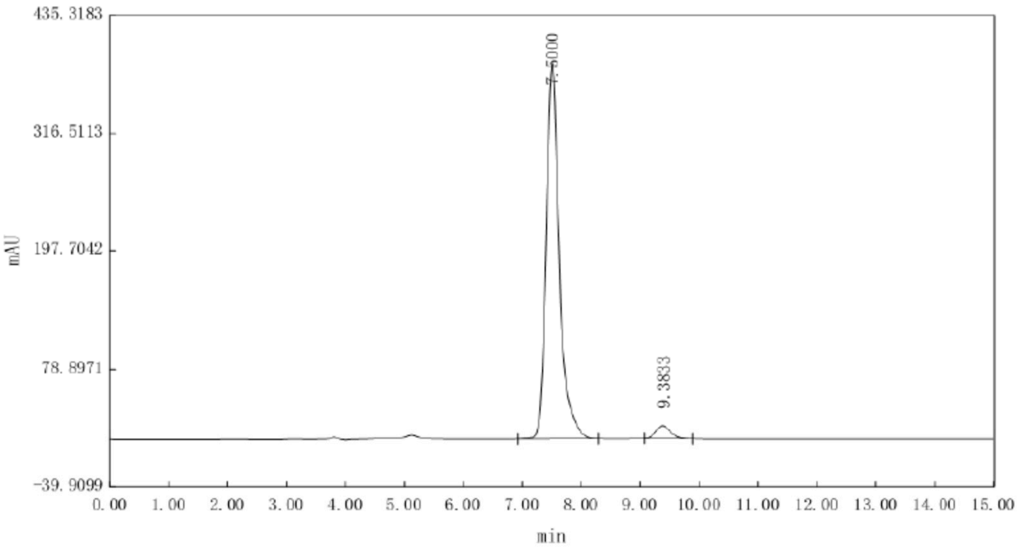

Peak	RT [min]	Height [mV]	Height %	Area[mV*s]	Area%
1	7.5000	394.8219	96.7024	6173.8823	96.3013
2	9.3833	13.4635	3.2976	237.1220	3.6987

(+)-3-(4-Chlorophenyl)-1-phenyl-3-(phenylamino)propan-1-one (11l)

Daicel Chiralpak AD-H *n*-hexane/*i*-propanol 3:2; flow rate = 1.0mL/min, λ = 254 nm

(rac)-3-(4-Nitrophenyl)-1-phenyl-3-(phenylamino)propan-1-one (11m)

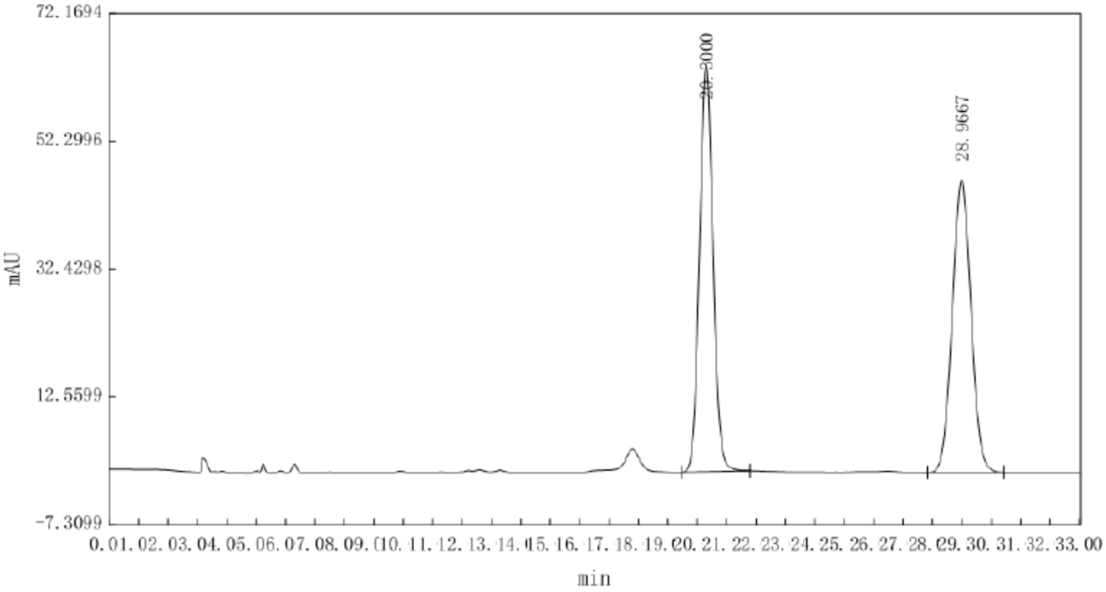

Peak	RT [min]	Height [mV]	Height %	Area[mV*s]	Area%
1	20.3000	65.5795	58.0871	2106.1484	50.0277
2	28.9667	47.3190	41.9129	2103.8191	49.9723

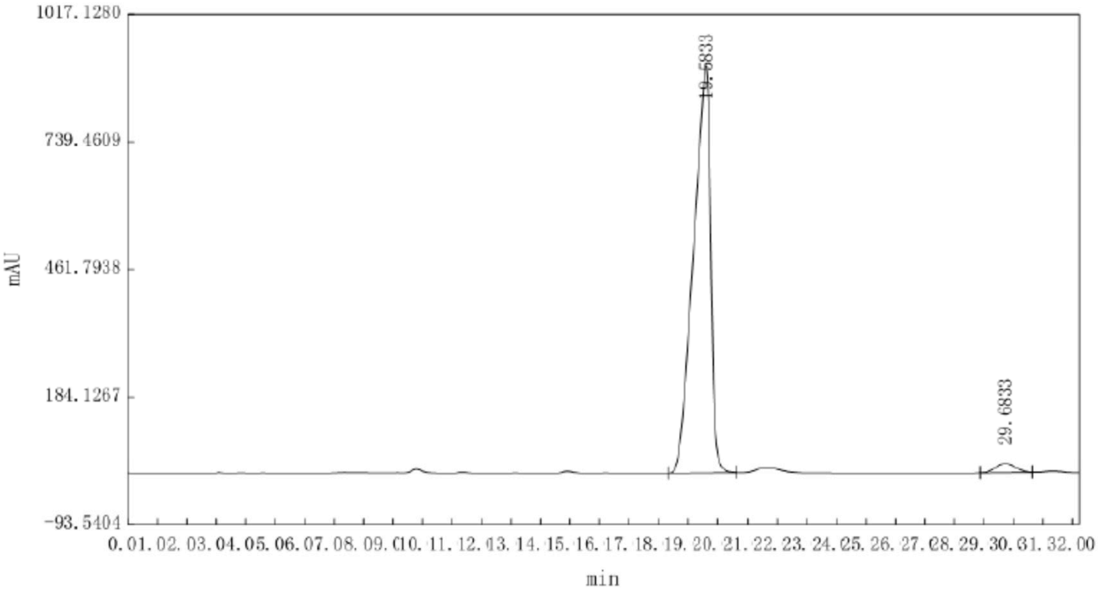

Peak	RT [min]	Height [mV]	Height %	Area[mV*s]	Area%
1	19.5833	924.2743	97.9251	37351.3815	97.5707
2	29.6833	19.5837	2.0749	929.9749	2.4293

(+)-3-(4-Nitrophenyl)-1-phenyl-3-(phenylamino)propan-1-one (11m)

Daicel Chiralpak AD-H *n*-hexane/*i*-propanol 4:1; flow rate = 1.0mL/min, λ = 254 nm

(rac)-1-phenyl-3-(phenylamino)-3-(thiophen-2-yl)propan-1-one (11n)

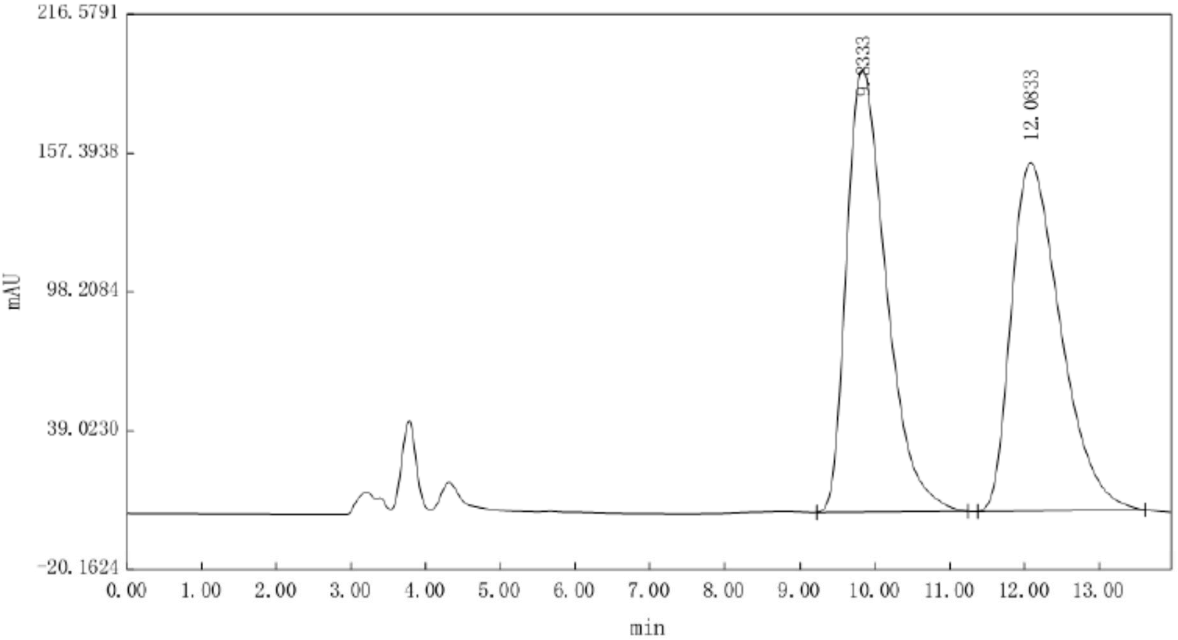

Peak	RT [min]	Height [mV]	Height %	Area[mV*s]	Area%
1	9.8333	196.3071	55.9480	7262.1460	50.5382
2	12.0833	154.5671	44.0520	7107.4741	49.4618

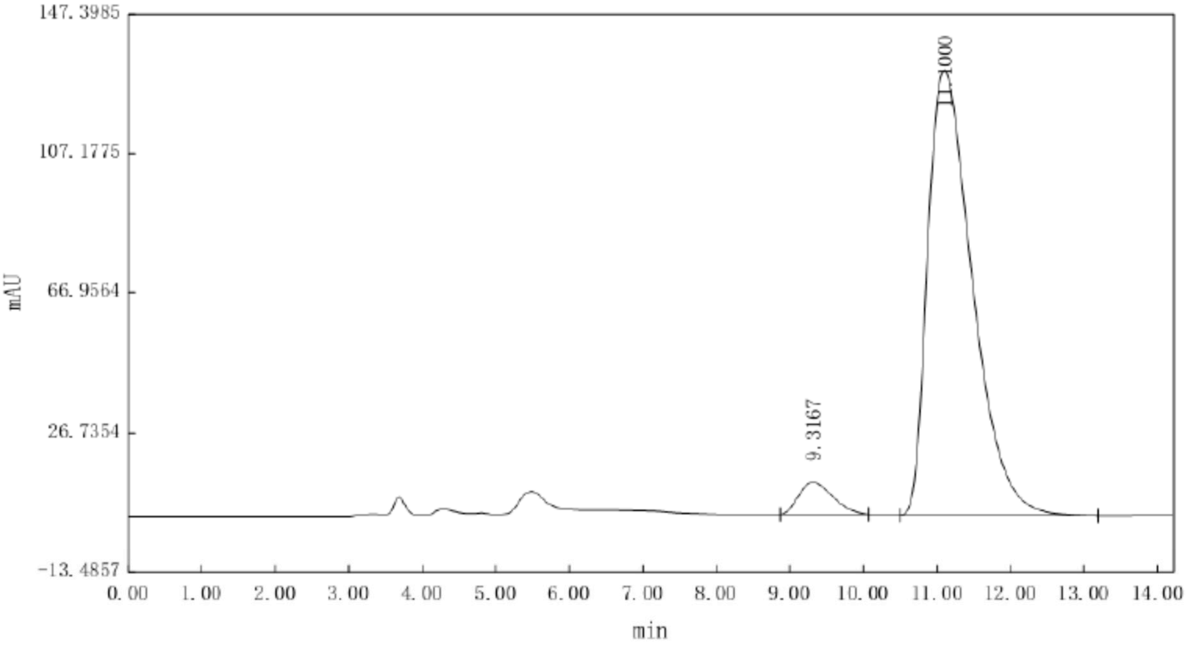

Peak	RT [min]	Height [mV]	Height %	Area[mV*s]	Area%
1	9.3167	9.7227	6.7861	310.1283	5.2068
2	11.1000	133.5504	93.2139	5646.0989	94.7932

(+)-1-phenyl-3-(phenylamino)-3-(thiophen-2-yl)propan-1-one (11n)

Daicel Chiralpak AD-H *n*-hexane/*i*-propanol 4:1; flow rate = 1.0mL/min, λ = 254 nm

(rac)-1-(3-Methoxyphenyl)-3-phenyl-3-(phenylamino)propan-1-one (11o)

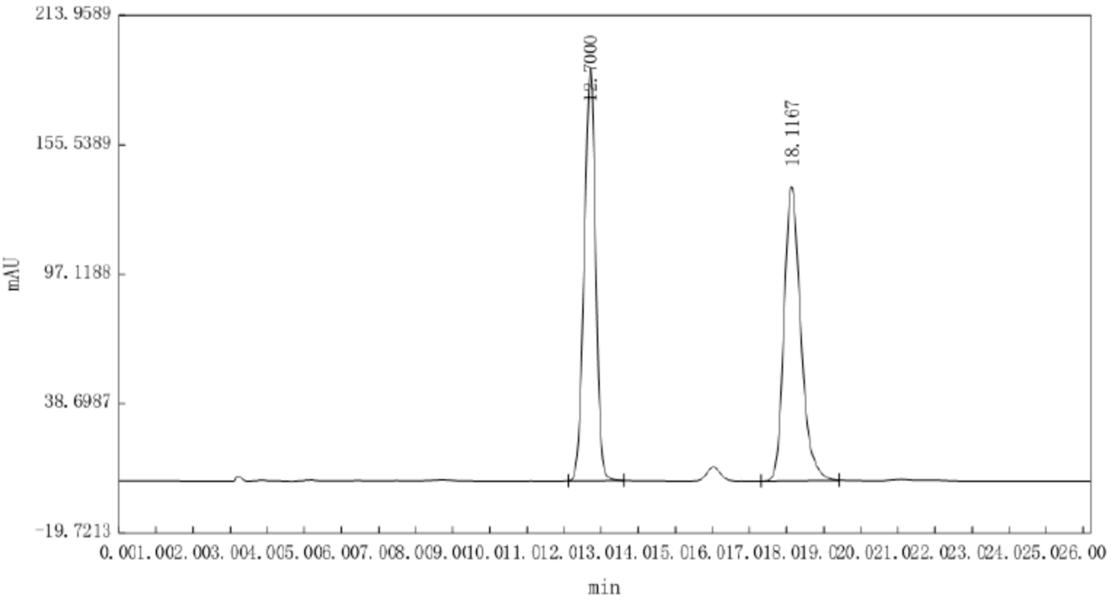

Peak	RT [min]	Height [mV]	Height %	Area[mV*s]	Area%
1	12.7000	194.4641	58.4261	4162.8226	49.1025
2	18.1167	138.3736	41.5739	4314.9983	50.8975

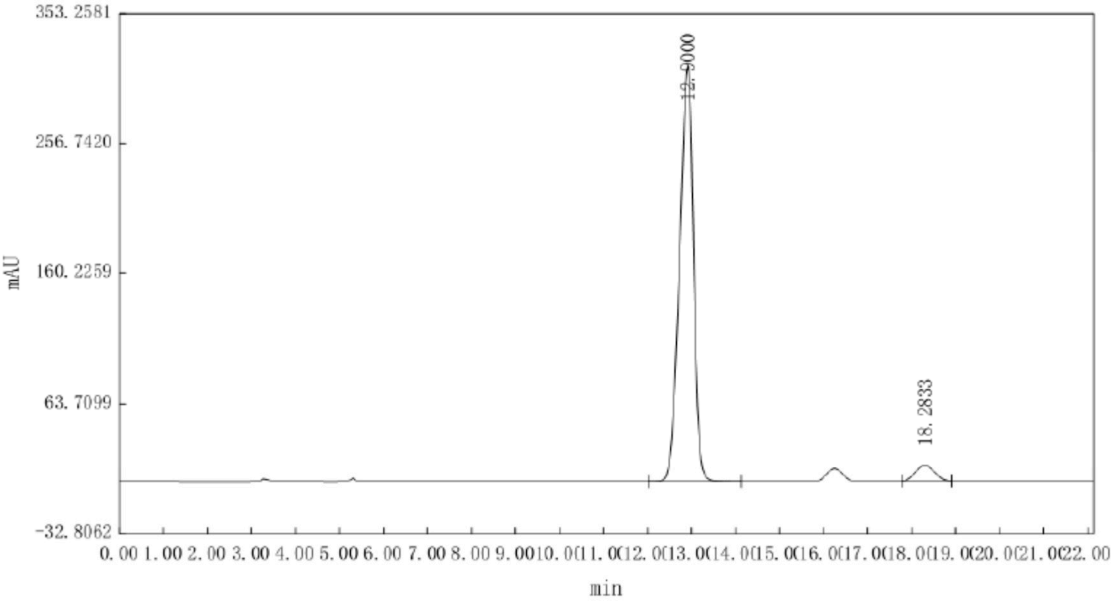

Peak	RT [min]	Height [mV]	Height %	Area[mV*s]	Area%
1	12.9000	321.4127	96.2619	7063.7528	95.0760
2	18.2833	12.4812	3.7381	365.8349	4.9240

(+)-1-(3-Methoxyphenyl)-3-phenyl-3-(phenylamino)propan-1-one (11o)

Daicel Chiralpak AD-H *n*-hexane/*i*-propanol 4:1; flow rate = 1.0mL/min, λ = 254 nm

(rac)-1-(4-Chlorophenyl)-3-phenyl-3-(phenylamino)propan-1-one (11q)

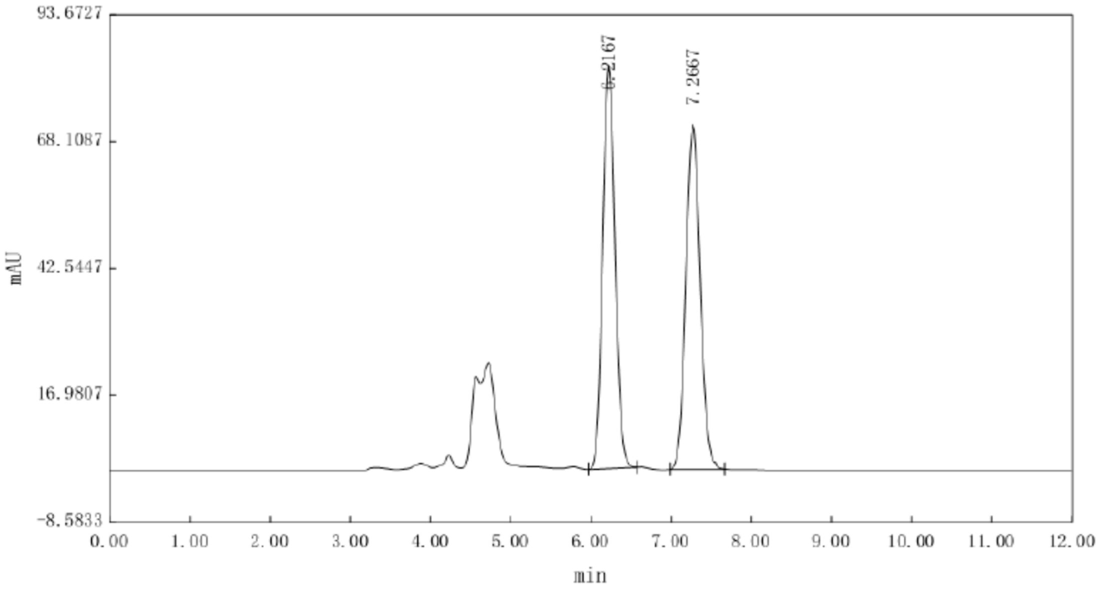

Peak	RT [min]	Height [mV]	Height %	Area[mV*s]	Area%
1	6.2167	84.5720	53.8998	899.3452	49.9153
2	7.2667	72.3340	46.1002	902.3970	50.0847

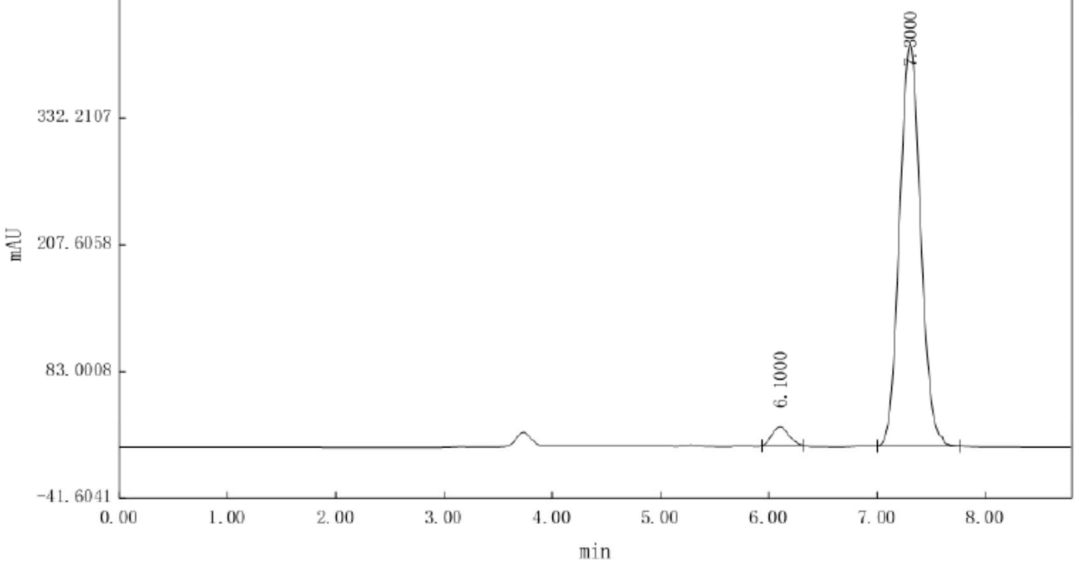

Peak	RT [min]	Height [mV]	Height %	Area[mV*s]	Area%
1	6.1000	19.0258	4.3951	206.5114	3.5745
2	7.3000	413.8644	95.6049	5570.8300	96.4255

(rac)-1-(4-Chlorophenyl)-3-phenyl-3-(phenylamino)propan-1-one (11q)

Daicel Chiralpak AD-H *n*-hexane/*i*-propanol 1:1; flow rate = 1.0mL/min, λ = 254 nm
